# Losartan attenuates sex-dependent hypertension, neuroinflammation, and cognitive impairment in the aging male sprague–dawley rat

**DOI:** 10.1007/s11357-024-01409-4

**Published:** 2024-12-03

**Authors:** Kayla M. Nist, Hannah Bard, Brannon McBride, Angela L. Capriglione, Jesse D. Moreira, David H. Farb, Richard D. Wainford

**Affiliations:** 1 Department of Anatomy and Neurobiology, Boston University Chobanian and Avedisian School of Medicine, Boston, MA USA; 2 Whitaker Cardiovascular Institute, Department of Medicine, Boston University Chobanian and Avedisian School of Medicine, Boston, MA USA; 3 Department of Pharmacology & Experimental Therapeutics, Boston University Chobanian and Avedisian School of Medicine, Boston, MA USA; 4 Department of Health Sciences, Sargent College, Boston University, Boston, MA USA; 5 Center for Systems Neuroscience, Boston University, Boston, MA USA; 6 Department of Medicine, Division of Cardiology, Emory University School of Medicine, 1750 Haygood Drive, Atlanta, GA N220 USA

**Keywords:** Aging, Hypertension, Neuroinflammation, Paraventricular nucleus of the hypothalamus, Cognitive impairment, Angiotensin II type 1 receptor

## Abstract

The prevalence of hypertension increases with age and is the leading modifiable risk factor for cognitive impairment and dementia. At present, the neural mechanisms promoting hypertension across the lifespan are incompletely understood. Using the Sprague–Dawley (SD) rat as a model of normal aging, we hypothesized (1) blood brain barrier (BBB) disruption and neuroinflammation in the paraventricular nucleus (PVN) of the hypothalamus enhances sympathetic tone and contributes to age-dependent hypertension, (2) age-dependent hypertension is associated with cognitive impairment, and (3) lowering blood pressure in aged rats with established hypertension improves cognitive function. We found male, but not female, rats develop age-dependent hypertension with enhanced sympathetic tone, BBB disruption, and neuroinflammation in the PVN. Aged hypertensive male rats also showed impairments in recognition and spatial memory. Utilizing pharmacological interventions, blood pressure was lowered in male rats with established hypertension using either losartan (LOS) or hydrochlorothiazide. However, only losartan improved recognition memory. Further, LOS reduced BBB disruption, microglial activation, astrocyte reactivity, and proinflammatory cytokine expression in the PVN which we speculate contributes to a decrease in blood pressure. These data show SD rats develop age-dependent hypertension and cognitive impairment in a sex-dependent manner. However, not all antihypertensive agents improve cognitive function equally as only losartan, an angiotensin II type 1 receptor antagonist (AT1R) improved recognition memory. Thus, AT1R antagonists represent a potential therapeutic approach for treating cognitive decline in the aging population.

**Supplementary Information:**

The online version contains supplementary material available at 10.1007/s11357-024-01409-4.

## Introduction

Hypertension is the leading modifiable risk factor for stroke, myocardial infarction, and chronic kidney disease [1]. Further, hypertension is of particular importance in the aging population as it impacts three in four US adults above the age of 65 [1]. While hypertension rates are lower in premenopausal women compared to age-matched men, there is a dramatic increase in the incidence of hypertension in postmenopausal women [2]. Hypertension has been identified as a key contributor to cognitive impairment and dementia—a major health issue in the aging population [3]. Moreover, it is suggested that nearly 40% of dementia cases can be prevented or delayed by treating underlying risk factors, such as hypertension [4]. Thus, improving our understanding of the relationship between hypertension and cognitive impairment is critical to potentially treat and prevent cognitive decline, particularly in the context of aging.

Male Sprague–Dawley (SD) rats develop age-dependent hypertension with enhanced sympathetic outflow [5], suggesting a similar pathology to human hypertension [6]; however, the neural mechanisms underlying age-dependent hypertension remain unknown. There are multiple central nervous system mechanisms that may be modulating sympathetic outflow leading to elevations in blood pressure, particularly those involving the paraventricular nucleus of the hypothalamus (PVN)—a cardio-regulatory brain nucleus central to the sympathetic control of blood pressure [7]. Recent data suggests when the PVN blood brain barrier (BBB), which provides protection to the brain from the peripheral environment, is disrupted in rats with hypertension, circulating factors can gain access to the parenchyma and cause neuroinflammation contributing to excess sympathetic outflow and hypertension [8]. Moreover, PVN neuroinflammation mediated by microglia and proinflammatory cytokines can drive elevations in sympathetic tone and the development of hypertension in young rodents [9–11]. However, this remains unaddressed as a potential mechanism underlying the development and maintenance of age-dependent hypertension.

Multiple human studies have reported a significant relationship between hypertension and cognitive impairment, [12, 13] with the magnitude of hypertension positively correlating with impairments in cognition. Critically, human studies identified midlife hypertension as a risk factor for the development of late life cognitive impairment [14–16]. While studies investigating a relationship between hypertension and cognitive function in rodents have been conducted, they have been limited to young animals. Studies in young angiotensin II infused (ANG II) mice found blood brain barrier disruption drives impairments in cognition [17]. Similarly, studies in young Dahl Salt Sensitive rats suggest neuroinflammation contributes to cognitive impairment which was prevented by angiotensin II type 1 receptor antagonist (AT1R) treatment [18]. However, the potential impact of blood pressure lowering treatments on the cognitive consequences of hypertension in aged rodents has yet to be addressed.

We hypothesized (1) blood brain barrier disruption and neuroinflammation in PVN enhances sympathetic tone and contributes to age-dependent hypertension, (2) age-dependent hypertension is associated with cognitive impairment, and (3) lowering blood pressure in aged rats with established hypertension improves cognitive function. To test this, we used male and female SD rats at 3, 8, and 16 months old to model normal aging. We assessed blood pressure, sympathetic tone, BBB permeability, neuroinflammation, and recognition and spatial memory. We then lowered blood pressure in aged male rats with established hypertension and cognitive impairment using either losartan (LOS), an AT1R antagonist, or hydrochlorothiazide (HCTZ), a sodium chloride co-transporter antagonist, and reassessed these parameters. Collectively, these studies provide new mechanistic insight into the sex-dependent development of age-dependent hypertension in SD rats and suggest a potential therapeutic approach for the treatment of cognitive impairment in the aging population.

## Methods

### Animals

Male and female SD rats aged 3, 8, and 16 months old (MO) (equivalent to ~ 20 (young adult), 45 (midlife), and 60 (aged) human years of age, respectively) were acquired from Envigo (Indianapolis, IN, USA) as previously published by our laboratory [5]. Rats were pair housed until surgical intervention or body weight requirements were exceeded in a 12:12-h light–dark cycle, temperature (20–26 °C) and humidity-controlled (30–70%) environment. Rats were allotted tap water and standard irradiated rodent diet (Envigo Teklab, WI, Teklab Global Diet #2918, 18% protein, 5% crude fat, 5% fiber, total NaCl content 0.6% [174 mEq Na + /kg]) ad libitum. All animal protocols used were approved by the Institutional Animal Care and Use Committee at Boston University Chobanian and Avedisian School of Medicine in agreement with guidelines set by the university and the National Institutes of Health *Guide for the Care and Use of Laboratory Animals*. Every possible step was taken to minimize pain and suffering. Euthanasia was performed as per the above guidelines, in accordance with the approved IACUC protocol number PROTO201800201 at Boston University.

### Surgical procedures

#### Acute femoral artery and femoral vein cannulation

Male and female SD rats (*N* = 5–6/group) were anesthetized using sodium methohexital (20 mg/kg intraperitoneally (i.p.), with 10 mg/kg administered intravenously (i.v.) as needed). Cannulation of the femoral artery and vein was performed as previously described [5, 19]. An incision was made in the left femoral triangle and the femoral artery and vein were dissected from the adjacent tissue. Using a cannula made from PE-50 tubing, the cannula was inserted into the femoral vein to allow for administration of i.v. anesthesia, isotonic saline during recovery periods, as well as hexamethonium administration. A PE-50 cannula was placed in the left femoral artery to record heart rate and mean arterial pressure (MAP). Cannulas were tied in place using sutures and the incision was closed. Rats were positioned in a Plexiglas rat holder and the cannula for the femoral artery was attached to an external pressure transducer, while the cannula for the femoral vein was attached to an infusion pump. A two-hour recovery period was allowed, during which an i.v. infusion of isotonic saline (20 μL/min) was performed and rats returned to full consciousness, with stable cardiovascular and renal function. After the two-hour recovery period, baseline MAP was continuously recorded over a one-hour period in conscious rats through the femoral artery cannula using the computer-driven data acquisition software, MP150 and AcqKnowledge 3.8.2 (BIOPAC, CA).

#### Intravascular infusion of dextrans

Male and female SD rats (*N* = 6/group) were anesthetized (30 mg/kg ketamine i.p., 3 mg/kg xylazine i.p.) [20] and an incision was made into the left carotid triangle. Muscles were mobilized to expose the carotid sheath. The carotid artery was dissected from surrounding tissues and isolated. A PE-50 cannula was placed into the exposed left common carotid artery and secured [8]. Rats were infused with fluorescein isothiocyanate-Dextran (FITC) at 10 kDa (10 mg/mL, FD10S, Sigma-Aldrich) and rhodamine B isothiocyanate-Dextran at 70 kDa (10 mg/mL, R9379, Sigma-Aldrich) over 60 s (~ 10 μL/1 s). Fluorescent dextrans were allowed to circulate for 20 min before animals were sacrificed via decapitation. An additional group of 3MO male rats (*N* = 6) were infused with hypertonic mannitol (1.4 mol/L, 2 mL per 200–250 g) 5 min before the dextran infusion as a positive control (Supplemental Fig. 1) [8]. After sacrifice, brain tissue was extracted and post-fixed in 4% paraformaldehyde (PFA) for 48–72 h, then immersed in 30% sucrose solution until sectioning.

#### Osmotic mini pump insertion

Male rats aged 16MO (*N* = 5–6/group) were treated with either losartan potassium (LOS; 3 mg/kg/day in sterile saline for 21 days; L0232, TCI) [19] or hydrochlorothiazide (HCTZ; 4 mg/kg/day in 50:50 DMSO:Saline for 14 days; H4759, Sigma) [5] using 2ML4 pumps (Alzet). In brief, rats were anesthetized using a ketamine/xylazine cocktail (30 mg/kg i.p. ketamine; 3 mg/kg i.p. xylazine), an incision was made in the subscapular region and a subcutaneous pocket was made. An osmotic mini pump was implanted, and the incision was closed with suture.

#### Transcardiac perfusion

Male and female SD rats were anesthetized with a ketamine/xylazine cocktail (30 mg/kg ketamine i.p., 3 mg/kg xylazine i.p.). Once anesthetic depth was reached and there was an absence of toe pinch response, the thoracic cavity was opened and a needle was placed in the heart attached to a perfusion pump. Rats were perfused with ice-cold 1X phosphate-buffered saline before perfusion with 4% PFA. Once fixation was achieved, perfusion was stopped and brains was harvested. Brains were post-fixed in PFA for 48–72 h before being moved to 30% sucrose for sectioning.

### In vivo studies

#### Assessment of vascular sympathetic tone

In animals that underwent acute femoral artery and vein cannulation, peak depressor response to ganglionic blockade (hexamethonium, 30 mg/kg, i.v., H2138, Sigma-Aldrich) was used to estimate sympathetic tone to the vasculature as previously described [5, 19]. Baseline blood pressure was defined as the average blood pressure that occurred during the ten minutes before the bolus of hexamethonium was administered. The lowest blood pressure measurement that occurred within five minutes of the administration of hexamethonium was used to calculate the difference compared with baseline blood pressure.

#### Novel object recognition task

For novel object recognition (NOR) testing [21], rats were placed in a 60 by 60 cm open topped behavioral testing arena with a video camera fixed above to record testing for manual analysis. Two different objects were placed in separate quadrants of the arena placed 6 inches from adjacent corners. For familiarization, rats were placed in the center of the arena and allowed to explore the environment and objects for 10 min. Following familiarization, rats were returned to their home cages for 2 h. Following the 2-h latency, rats were returned to the testing arena, this time with a novel object replacing one of the familiar objects. Rats were allowed to explore the environment and objects for 10 min to complete the test portion of the assessment. After testing rats were returned to their home cages. The testing arena and objects were thoroughly cleaned between each test with 10% ethanol.

#### Object location task

Object location (OLT) testing [22] was completed in the same open topped behavioral testing arena as described in the *Novel Object Recognition Task*. Familiarization was carried out in the same manner. Following the 2-h latency, rats were returned to the testing arena, this time with one of the familiar objects placed in the opposite quadrant from its original location. Following 10 min of exploration, testing was complete and rats were returned to their home cages and the arena was cleaned with 10% ethanol.

In 16MO male rats treated with losartan or hydrochlorothiazide, novel object recognition task and object location task were completed at baseline prior to the initiation of treatment and at the end of the treatment period in order to assess the impact of treatment on cognitive performance in a within subject design.

The time a rat spent investigating each object separately was measured in order to calculate time investigating the novel object or location and the total time spent investigating both objects. These measures were used to calculate the familiarization index, discrimination index, and the percent of time exploring the novel object/location.

Familiarization score was calculated as the absolute value of the time spent at one object subtracted by the time spent at the other object divided by the total time spent investigating the objects. This score was used to assess any preference a rat may have for one object over the other.

$$Familiarization=\frac{\left(time\, spent\, at\, right\, object\right)-\left(time\, spent\, at\, left\, object\right)}{time\, spent\, at\, both}$$

Discrimination index is a measure of sensitivity and is assessed as the time spent investigating the novel object/location minus the time spent investigating the familiar object/location divided by the total time spent investigating both objects/locations. Percent of time exploring the novel object/location was assessed time spent investigating the novel object/location divided by the total amount of time spent investigating.

$$Discrimination\, index=\frac{\left(time\, spent\, at\, novel\, object\, or\, location\right)-\left(time\, spent\, at\, familiar\, object\, or\, location\right)}{total\, time\, spent\, at\, both}$$

### Molecular techniques

#### Assessment of plasma norepinephrine content and plasma estradiol content

Plasma taken from male and female SD rats following conscious decapitation was used to measure plasma norepinephrine (NE) via ELISA (IB89552, IBL America) according to the manufacturer’s directions as previously performed by our laboratory [5, 11]. To measure plasma estradiol, plasma taken from female rats following conscious decapitation was used to measure 17β estradiol via ELISA (IB79329, IBL America) according to the manufacturer’s directions as previously performed by our laboratory [5].

#### Assessment of plasma progesterone content

Plasma progesterone was measured at the University of Mississippi Medical Center Mass Spectrometry Core using LC–MS/MS analysis. All reagents used were high throughput liquid chromatography grade and purchased from Millipore Sigma including water, methanol, acetonitrile, progesterone materials, and isotope-labeled internal standard for progesterone (Progesterone-D9). Cold-induced phase separation was used to extract progesterone from plasma. Briefly, 100 µL of plasma and 10 µL of Internal standard solution (100 ng/mL isotype-labeled internal standards) were added to 200 µL cold acetonitrile and vortexed before an additional 100 µL HPLC water was added. The samples were centrifuged (10,000 g, 2 min, 4℃) and stored at − 20℃ for 30 min to induce phase separation. After the first incubation, the upper layer (100 µL) was transferred to a separate tube containing 100 µL NaHCO3 and then incubated 10 min at 65℃. The samples were then placed back into − 20℃ for 45 min for phase separation. After 45 min, the upper phase (50 µL) was added to 50 µl HPLC water and used for LC–MS/MS analysis. Equipment for detection consisted of an Eksigent M5 microLC with autosampler coupled to a Sciex Qtrap 7500 mass spectrometer. For LC separation, Formic acid in water (0.1% v/v) and formic acid in 75% acetonitrile (0.1% v/v) were used as mobile phases A and B, respectively. The gradient was set as 0–1 min 20–40% B, 1–4.5 min 40–95% B, 4.5–5 min 95% B, 5–5.5 min 95–2%B, and 5.5–8 min 2% B. A Phenomenex Luna 5 µm C18 100 Å (150 × 0.3 mm) LC column was used. The flow rate and column temperature are set at 10 µL/min and 30℃ with an injection volume of 5 µL. The electrospray ionization parameters are as follows: curtain gas (40.0 psi), CAD gas (medium), ionspray voltage (4000 V), source temperature (350 °C), ion source gas 1 (30.0 psi), ion source gas 2 (70.0 psi), and Q0 dissociation (40 V). The multiple reaction monitoring transitions used for quantification were as follows: progesterone, 315.2 → 97.0, and progesterone-D9, 324.2 → 99.9. All peaks were automatically integrated via SCIEX OS AutoPeak integration algorithm and analyzed in Sciex OS v.3.3.

#### Vascular integrity of the BBB

Following intravascular infusions of dextrans, post-fixed tissue was sectioned at 40 µm and cryoprotected. PVN tissue was selected between bregma − 1.6 and − 2.16 mm, washed in phosphate-buffered saline (PBS) and mounted on gelatin-subbed slides. Coverslips were secured using Prolong Diamond Antifade Mountant with DAPI (P36971, Invitrogen). Analysis is described below.

#### Immunohistochemistry

Immunohistochemistry was performed as previously described by our laboratory [5, 11]. Brain tissue from perfused rats was sectioned at 40 μm on a cryostat and cryoprotected. Multiple sections sampling all levels of the PVN were selected using Paxinos & Watson’s *The Rat Brain* atlas, between bregma − 1.6 mm and bregma − 2.16. For immunohistochemistry, sections were washed in 0.1 M PBS 3 times for 10 min each and then incubated in 0.3% hydrogen peroxide for 30 min. Sections were then blocked and permeabilized in PBS-diluent (0.01 M PBS with 3% normal horse serum and 0.25% Triton X-100) for 2 h before incubation in primary antibodies mouse OX-42/CD11b/c for microglia [11] or mouse glial fibrillary acidic protein (GFAP) for astrocytes [23] for 2 h at room temperature and then incubated overnight at 4℃. All antibody information can be found in Supplemental Table 1. Following incubation in primary antibodies, sections were washed in PBS then incubated in biotinylated goat anti-mouse IgG (1:100, BA-9200, Vector Laboratories). Sections were washed again in PBS and incubated in avidin and biotin. Sections were developed in 0.04% 3,3′-diaminobenzidene and 0.04% nickel ammonium sulfate in 0.1 M PBS, mounted on gelatin-subbed slides and dehydrated overnight. Coverslips were secured using Permount mounting medium. Negative controls were performed. One control was performed with the omission of the primary antibody and the inclusion of the secondary antibody, while another control was performed with the inclusion of the primary antibody and the omission of the secondary antibody (Supplemental Figs. 2–4).

#### Immunofluorescence

For immunofluorescence, PVN sections were selected as described above. Tissue was washed in PBS 3 times for 10 min each and blocked in PBS-diluent for 2 h (0.01 M PBS with 3% normal goat serum and 0.25% Triton X-100). Sections were then incubated in primary antibodies for mouse interleukin 6 (IL-6) [24] or mouse tumor necrosis factor-⍺ (TNF-⍺) [25] at room temperature for 2 h and then incubated overnight at 4℃. All antibody information can be found in Supplemental Table 1. Tissue was brought to room temperature and washed 3 times for 10 min each and incubated in appropriate secondary antibodies for goat anti-mouse AlexaFluor 594 (1:500, A-11005, Invitrogen) for 2 h. At the end of the incubation, sections were washed 3 times for 10 min each and mounted on gelatin-subbed slides and allowed to dry. Coverslips were secured using Prolong Diamond Antifade Mountant with DAPI counterstain. Negative controls were performed. One control was performed with the omission of the primary antibody and the inclusion of the secondary antibody, while another control was performed with the inclusion of the primary antibody and the omission of the secondary antibody (Supplemental Fig. 2–4).

#### Microscopy and image analysis

Sections were imaged on a Keyence BZ-9000 Fluorescence Microscope with brightfield capabilities. Images were captured at 10X, 20X, and 40X magnifications. Dichroic filters for Texas Red at 585 nm, GFP at 495 nm, and DAPI at 400 nm were used. A representative schematic of PVN neuroanatomy at 10X is included in Supplemental Fig. 5.

For assessment of BBB integrity, 20X images of FITC and Rhodamine were obtained and opened in FIJI/ImageJ. The images were converted to 16-bit, and the colocalization threshold tool was used to isolate matching signal in both images. Images were converted to binary, and the FITC image was subtracted from the colocalization image in order to expose FITC only signal. The percent area of FITC extravasation was calculated by using the threshold tool to select all areas positive for signal within the PVN.

For microglia analysis, images of the left and right PVN were taken at 40X magnification in order to assess morphology and perform Sholl analysis as previously described by our laboratory [11]. A 200 µm by 200 µm box was randomly selected and used for analysis. Microglia were deemed active if a majority of the process lengths were less than that of the diameter of the soma. The number of active microglia and the total number of active microglia were measured. For Sholl analysis, 8 individual microglia from each 40X image were selected at random and cropped for isolation using FIJI/ImageJ. The area of the individual microglia was selected using the threshold tool and the center of the specific randomly selected microglia was identified. Concentric rings 1 µm apart were placed on the image and the number of branching intersections for each randomly selected microglia were calculated (Supplemental Fig. 6).

For astrocytes (GFAP), IL-6, and TNF-⍺ analysis, 20X images of the left and right PVN were used to assess protein expression as total percent area. Images were converted to 8-bit, and positive areas were selected using the threshold tool, and particle analysis was performed.

#### Statistical analysis

All data are presented as mean ± standard deviation with *p* < 0.05 as significance. Pearson’s *r* correlation was performed to assess correlation with age and blood pressure as variables. A one-way ANOVA was used to assess group differences for all analyses with age as a set variable. Post-hoc analysis was performed using a Tukey test. For behavioral comparison between 16 MO male rats and LOS or HCTZ treatment, a repeated measures *t*-test was used. Prism 9 (GraphPad Software, CA) was used to carry out statistical analysis.

## Results

**Fig. 1:**
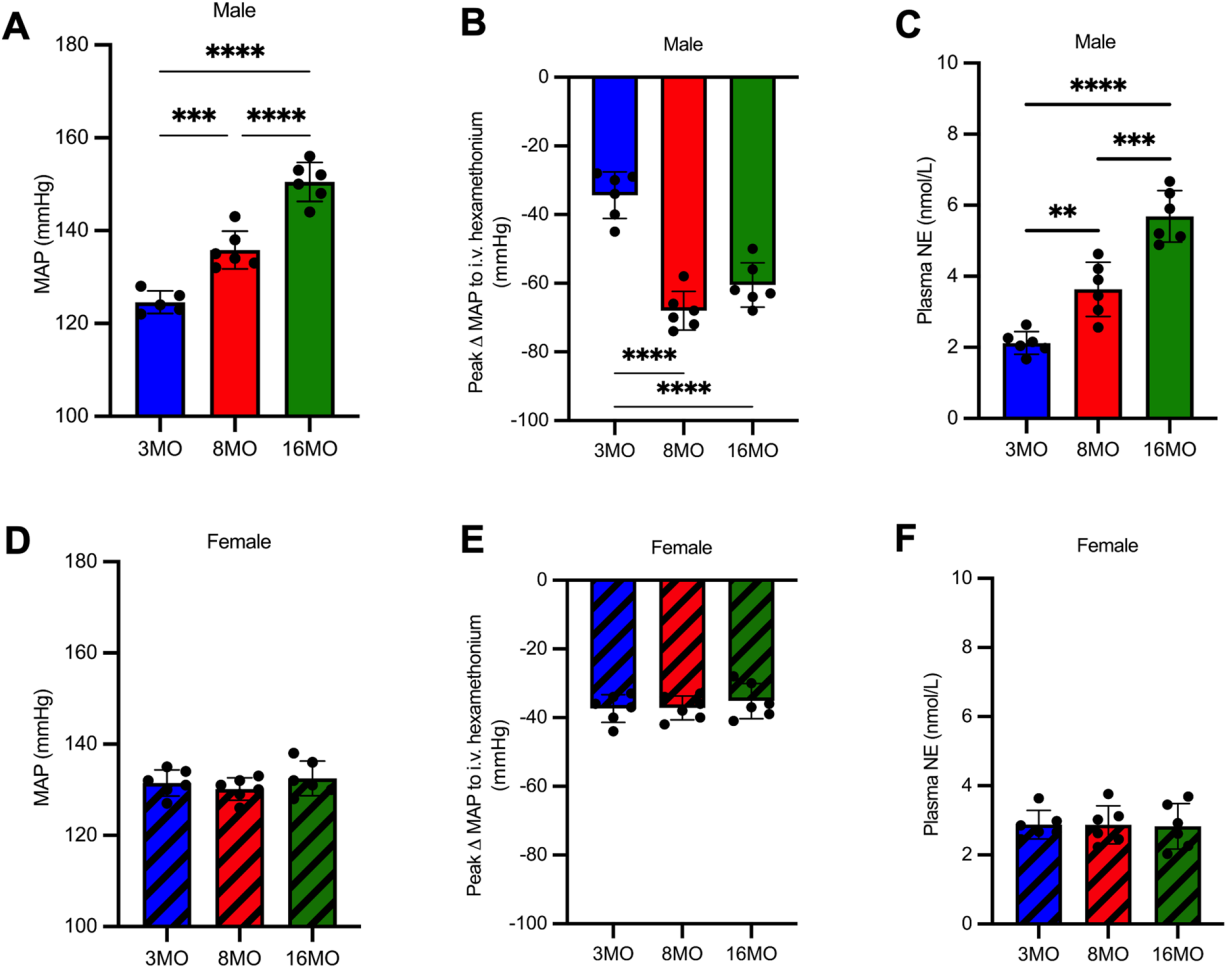
Male, but not female, Sprague-Dawley rats develop age-dependent hypertension and sympathoexcitation. (**A**) Mean arterial pressure (MAP; mmHg) measured via acute instrumentation in conscious 3, 8, and 16 month old (MO) male rats (*n* = 5–6/group). (**B**) Peak change in MAP to ganglionic blockade with hexamethonium (30 mg/kg I.V.) in male rats (*n* = 6/group). (**C**) Plasma norepinephrine (NE) content (nmol/L) in male rats (*n* = 6/group). (**D**) MAP measured via acute instrumentation in conscious 3, 8 and 16 month old female rats (*n* = 6/group). (**E**) Peak change in MAP to ganglionic blockade with hexamethonium in female rats (*n* = 6/group). (**F**) Plasma NE content (nmol/L) in female rats (*n* = 6/group). Statistical significance was determined using one-way ANOVA with a post-hoc Tukey test, **p < 0.01, ***p < 0.005, ****p < 0.0001

Male, but not female, SD rats develop age-dependent hypertension and sympathoexcitation. There was a progressive increase in MAP in aging male SD rats (Fig. 1A). Sympathetic tone to the vasculature increased to a similar magnitude in both 8 and 16MO male rats (Fig. 1B) and global sympathetic tone increased with age in male SD rats (Fig. 1C). Female rats showed no change in blood pressure or sympathetic tone with age (Fig. 1D–F). Elevations in blood pressure and global sympathetic tone positively correlate with increased age in male (Pearson’s *r* correlation; MAP: *r* = 0.9502, *p* < 0.0001; plasma NE: *r* = 0.09282, *p* < 0.0001), but not female rats (Pearson’s *r* correlation; MAP: *r* = 0.1739, *p* = 0.4902; plasma NE: *r* = -0.0402, *p* = 0.8741). In female rats, plasma 17β-estradiol increased at 8MO; however, there was no change between 3 and 16MO (Supplemental Fig. 7). Plasma progesterone did not change with age (Supplemental Fig. 7).

**Fig. 2:**
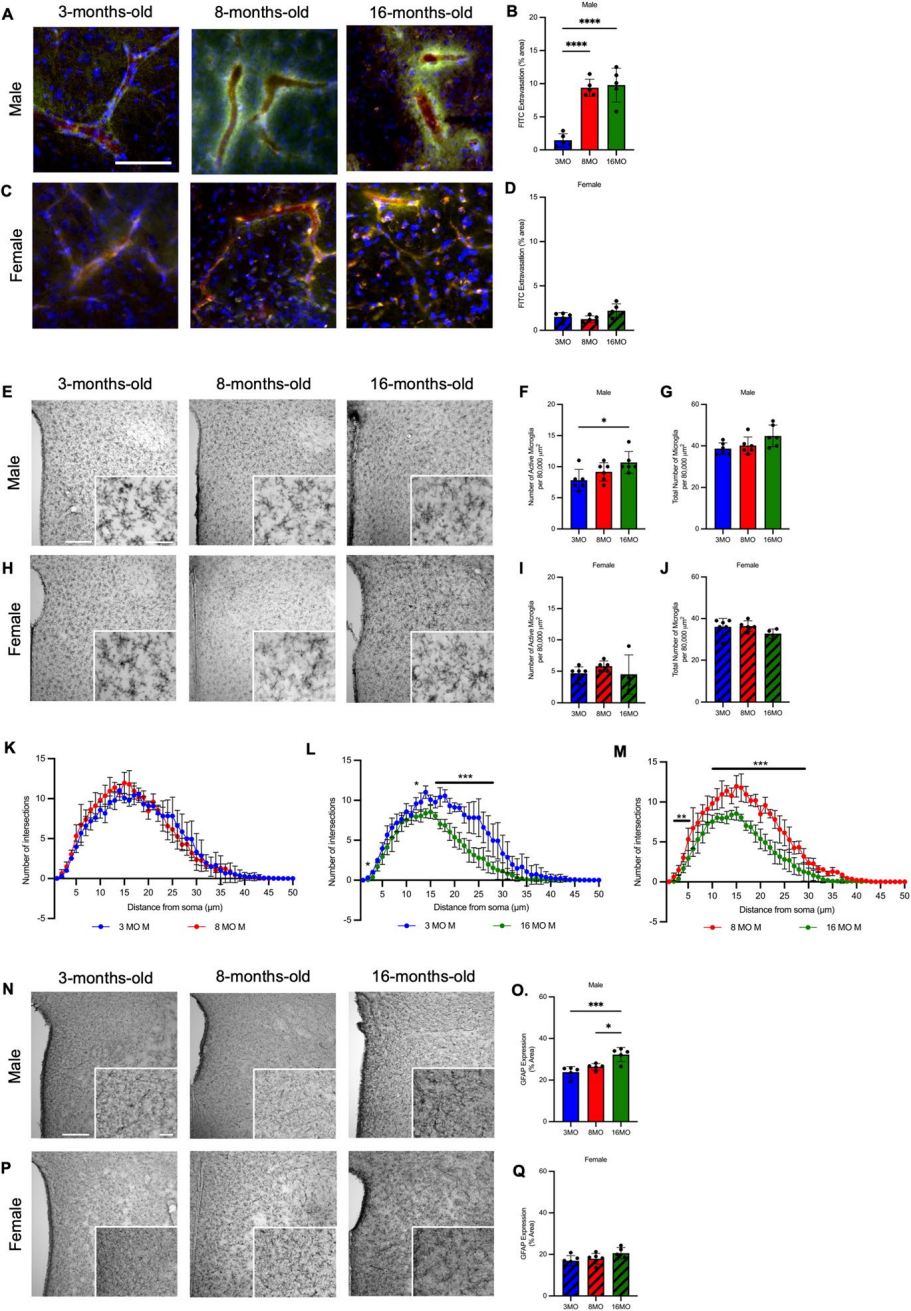
Male, but not female, Sprague–Dawley rats develop BBB disruption and neuroinflammation in the PVN with age. **A** Representative photomicrographs (captured at 10 × magnification) from level 2 of the PVN (bregma − 1.72 to − 2.04 mm) of FITC extravasation as a measure of BBB disruption in male rats at 3, 8, and 16 months old; scale bar = 200 μm. **B** FITC extravasation in level 2 of the PVN in aging male rats (*n* = 6/group). **C** Representative photomicrographs (captured at 10X magnification) from level 2 of the PVN (bregma − 1.72 to − 2.04) of FITC extravasation as a measure of blood brain barrier disruption in female rats at 3, 8, and 16 months old; Scale bar shown in A. **D** FITC extravasation in level 2 of the PVN in aging female rats (*n* = 6/group). **E** Representative photomicrographs (captured at 10X magnification with 40X magnification inset) from level 2 of the PVN (bregma − 1.72 to − 2.04 mm) of microglia (stained using OX-42/CD11b/c) in male rats at 3, 8, and 16 months old; scale bar (10X) = 200 μm; scale bar (40X) = 50 μm. **F** Number of active microglia per 80,000 μm^2^ in level 2 of the PVN in aging male rats (*n* = 6/group). **G** Total number of microglia per 80,000 μm^2^ in level 2 of the PVN in aging male rats (*n* = 6/group). **H** Representative photomicrographs (captured at 10X magnification with 40X magnification inset) from level 2 of the PVN (bregma − 1.72 to − 2.04) of microglia (stained using OX-42/CD11b/c) in female rats at 3, 8, and 16 months old; scale bars shown in E. **I** Number of active microglia per 80,000 μm^2^ in level 2 of the PVN in aging female rats (*n* = 6/group). **J** Total number of microglia per 80,000 μm.^2^ in level 2 of the PVN in aging female rats (*n* = 6/group). **K** Sholl analysis expressed as number of intersections as a function of distance from the soma (μm) in 8 microglia per rat from level 2 of the PVN in 3 vs. 8 month old male (M) rats (*n* = 6/group). **L** Sholl analysis expressed as number of intersections as a function of distance from the soma (μm) in 8 microglia per rat from level 2 of the PVN in 3 vs. 16 month old male (M) rats (*n* = 6/group). **M** Sholl analysis expressed as number of intersections as a function of distance from the soma (μm) in 8 microglia per rat from level 2 of the PVN in 8 vs 16 month old male (M) rats (*n* = 6/group). **N** Representative photomicrographs (captured at 10X magnification with 20X magnification inset) from level 2 of the PVN (bregma − 1.72 to − 2.04 mm) of astrocytes (stained using glial fibrillary acidic protein (GFAP)) in male rats at 3, 8, and 16 months old; scale bar (10X) = 200 μm; scale bar (20X) = 50 μm. **O** GFAP expression presented as percent area of level 2 of the PVN in aging male rats (*n* = 6/group). **P** Representative photomicrographs (captured at 10X magnification with 20X magnification inset) from level 2 of the PVN (bregma − 1.72 to − 2.04) of astrocytes (stained using GFAP) in female rats at 3, 8, and 16 months old; scale bars shown in N. **Q** GFAP expression presented as percent area of level 2 of the PVN in aging female rats (*n* = 6/group). Statistical significance was determined using one-way ANOVA with a post-hoc Tukey test, *p < 0.05, ***p < 0.005, and ****p < 0.0001. For Sholl analysis (K–M), Student’s *t*-test was used to determine statistical significance, *p < 0.05, **p < 0.01, and ***p < 0.005

Male, but not female, SD rats have a disrupted BBB in the PVN. BBB disruption, as evidenced by significant FITC-dextrans (10 kDa) extravasation into the parenchyma of the PVN was present to the same extent in male rats at 8 and 16MO (Fig. 2A, B, Supplemental Fig. 8). In contrast, female rats did not exhibit FITC extravasation in the PVN with age (Fig. 2C, D, Supplemental Fig. 8). To validate dextrans extravasation as an assessment of BBB permeability, a separate group of 3MO male rats was infused with hyperosmolar mannitol, prior to the co-infusion of dextrans as a positive control. While untreated 3MO male rats maintained an intact PVN BBB, 3MO male rats treated with mannitol had increased FITC extravasation indicative of increased BBB permeability (Supplemental Fig. 1).

**Fig. 3:**
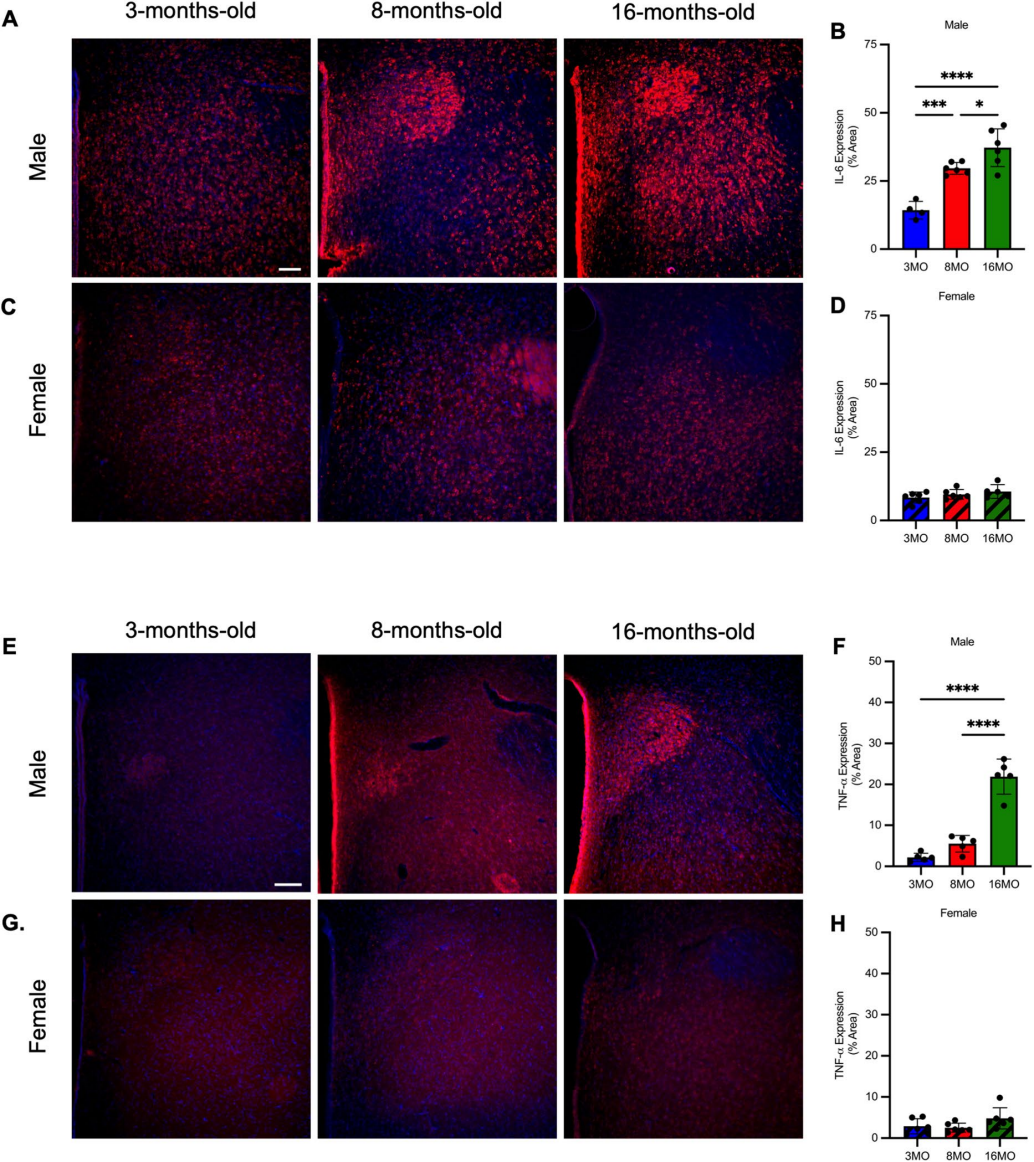
Male, but not female, Sprague–Dawley rats exhibit increased proinflammatory cytokine expression in the PVN with age. **A** Representative photomicrographs of interleukin-6 (IL-6) expression in level 2 of the PVN (bregma − 1.72to − 2.04 mm) of male rats at 3, 8, and 16 months old. **B** IL-6 expression as percent area of the PVN in male Sprague–Dawley rats (*N* = 5–6/group). **C** Representative photomicrographs of IL-6 expression in level 2 of the PVN (bregma − 1.72 to − 2.04 mm) of female rats at 3, 8, and 16 months old. **D** IL-6 expression as percent area of the PVN in female Sprague–Dawley rats (*N* = 5–6/group). **E** Representative photomicrographs of tumor necrosis factor-⍺ (TNF-⍺) expression in level 2 of the PVN (bregma − 1.72 to − 2.04 mm) of male rats at 3, 8, and 16 months old. **F** TNF-⍺ expression as percent area of the PVN in male Sprague–Dawley rats (*N* = 5–6/group). **G** Representative photomicrographs of TNF-⍺ expression in level 2 of the PVN (bregma − 1.72 to − 2.04 mm) of female rats at 3, 8, and 16 months old. **H** TNF-⍺ expression as percent area of the PVN in female Sprague–Dawley rats (*N* = 5–6/group). Scale bar = 200 μm. Statistical significance was determined using one-way ANOVA with a post-hoc Tukey test of significance, *p < 0.05, ***p < 0.005, and ****p < 0.0001

Neuroinflammation increases in the PVN of male, but not female, SD rats. Morphological assessment of microglia was performed to quantify the number of active and the total number of microglia in the PVN. The number of active (hypertrophic or ameboid) microglia in the PVN increased with age in male rats (Fig. 2E, F, Supplemental Fig. 9) while the total number of microglia had no change with age (Fig. 2E, G, Supplemental Fig. 9). Sholl analysis for branching complexity revealed that 16MO rats had a significant decrease in branching complexity compared to 3MO and 8MO rats, indicative of increased microglial activation (Fig. 2L, M). No difference detected between 3 and 8MO rats (Fig. 2K). Female rats had no change in the number of active or total number of microglia in the PVN (Fig. 2H–J, Supplemental Fig. 9). Astrocyte reactivity was assessed by tissue density of GFAP expression. Consistent with the increase in active microglia, GFAP expression increased in male rats (Fig. 2N, O, Supplemental Fig. 10), but not female rats with age (Fig. 2P, Q, Supplemental Fig. 10). Cytokine expression was analyzed as percent area of the PVN positive for IL-6 or TNF-⍺ staining. Male rats had a significant increase in both IL-6 and TNF-⍺ expression with age (Fig. 3A, B, E, F, Supplemental Figs. 11&12) while female rats showed no change in expression of either cytokine (Fig. 3C, D, G, H, Supplemental Figs. 11&12).

**Fig. 4:**
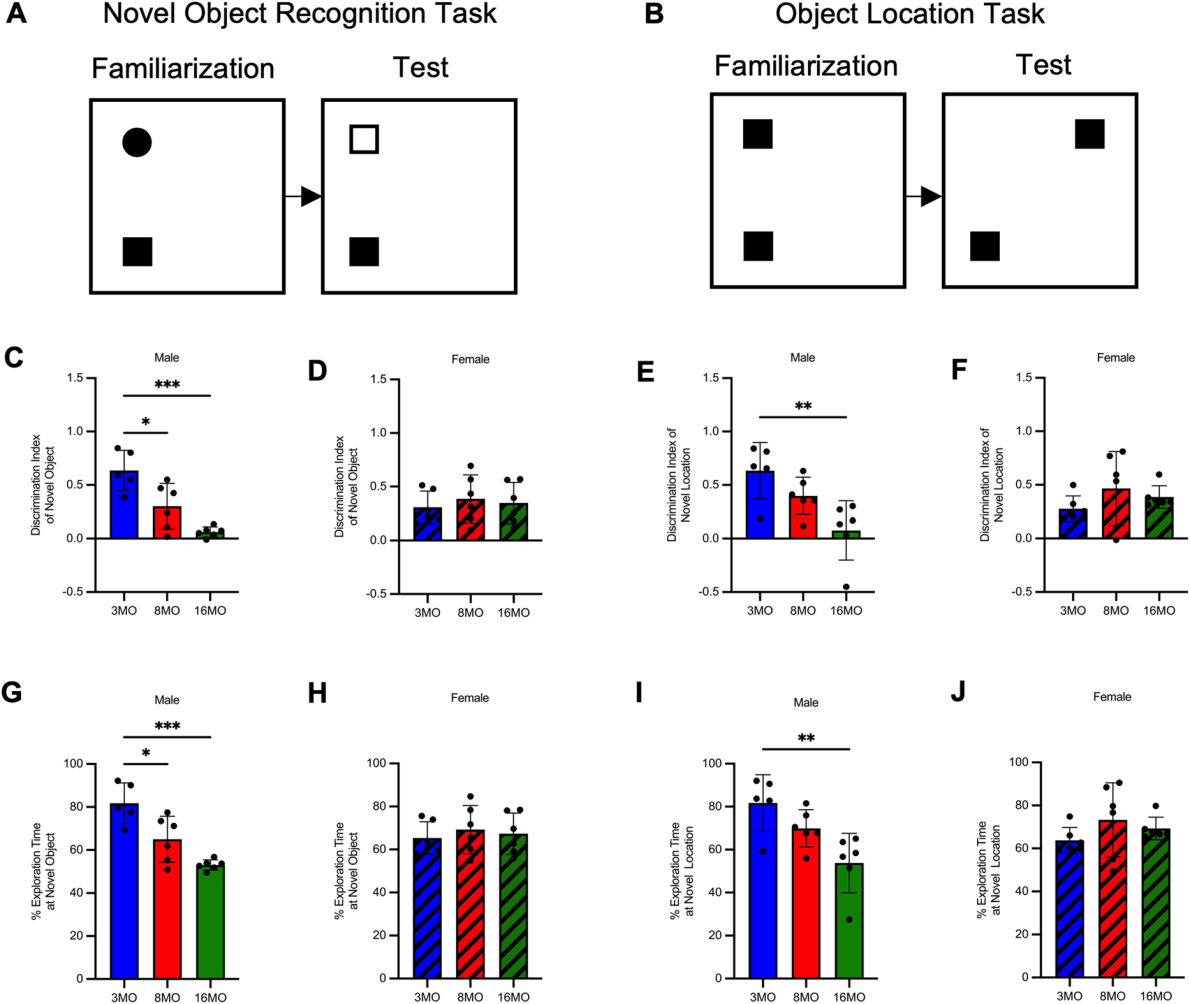
Male, but not female, Sprague–Dawley rats develop cognitive impairment. **A** Representative schematic of the novel object recognition task—a measure of recognition memory. **B** Representative schematic of the object location task—a measure of spatial memory. **C** Discrimination index of novel object in 3, 8 and 16 month old male rats (*N* = 5–6/group). **D** Discrimination index of novel object in 3, 8 and 16 month old female rats (*N* = 5–6/group). **E** Discrimination index of novel location in 3, 8 and 16 month old male rats (*N* = 5–6/group). **F** Discrimination index of novel location in 3, 8 and 16 month old female rats (*N* = 5–6/group). **G** Time spent at novel object (expressed as percent of total time) in 3, 8 and 16 month old male rats (*N* = 5–6/group). **H** Time spent at novel object (expressed as percent of total time) in 3, 8 and 16 month old female rats (*N* = 5–6/group). **I** Time spent at novel location (expressed as percent of total time) in 3, 8 and 16 month old male rats (*N* = 5–6/group). **J** Time spent at novel location (expressed as percent of total time) in 3, 8 and 16 month old female rats (*N* = 5–6/group). Statistical significance was determined using one-way ANOVA with a post-hoc Tukey test of significance, *p < 0.05, **p < 0.01, and ***p < 0.005

Recognition and spatial memory are impaired in hypertensive male, but not normotensive female, SD rats with age. On familiarization, male and female rats showed no preference in either task (Table 1). Aged male rats had decreased discrimination index on both tasks and spent less time at the novel object or location, indicative of impairments in recognition and spatial memory with age (Fig. 4C, E, G, I). Female rats showed no change in discrimination index or time spent at novel object or location with age, indicative of no age-related impairments in recognition or spatial memory (Fig. 4D, F, H, J).

**Table 1:** Familiarization from novel object recognition task and object location tasks in 3-, 8-, and 16-month-old male and female Sprague–Dawley rats

Age and sex	Novel object recognition task	Object location task
	Familiarization index	Familiarization index
3 MO male	0.038 ± 0.146	0.008 ± 0.085
8 MO male	0.092 ± 0.227	0.072 ± 0.329
16 MO male	0.033 ± 0.046	− 0.006 ± 0.059
3 MO female	0.013 ± 0.107	− 0.003 ± 0.127
8 MO female	0.300 ± 0.341	0.007 ± 0.175
16 MO female	0.035 ± 0.154	0.109 ± 0.226

Familiarization index, defined as time spent at left object minus time spent at right object divided by time spent at both objects, calculated for familiarization of the novel object recognition and object location tasks in 3, 8, and 16 month old (MO) male and female Sprague–Dawley rats. Data is presented as group mean ± standard deviation, *N* = 5–6/group

**Fig. 5:**
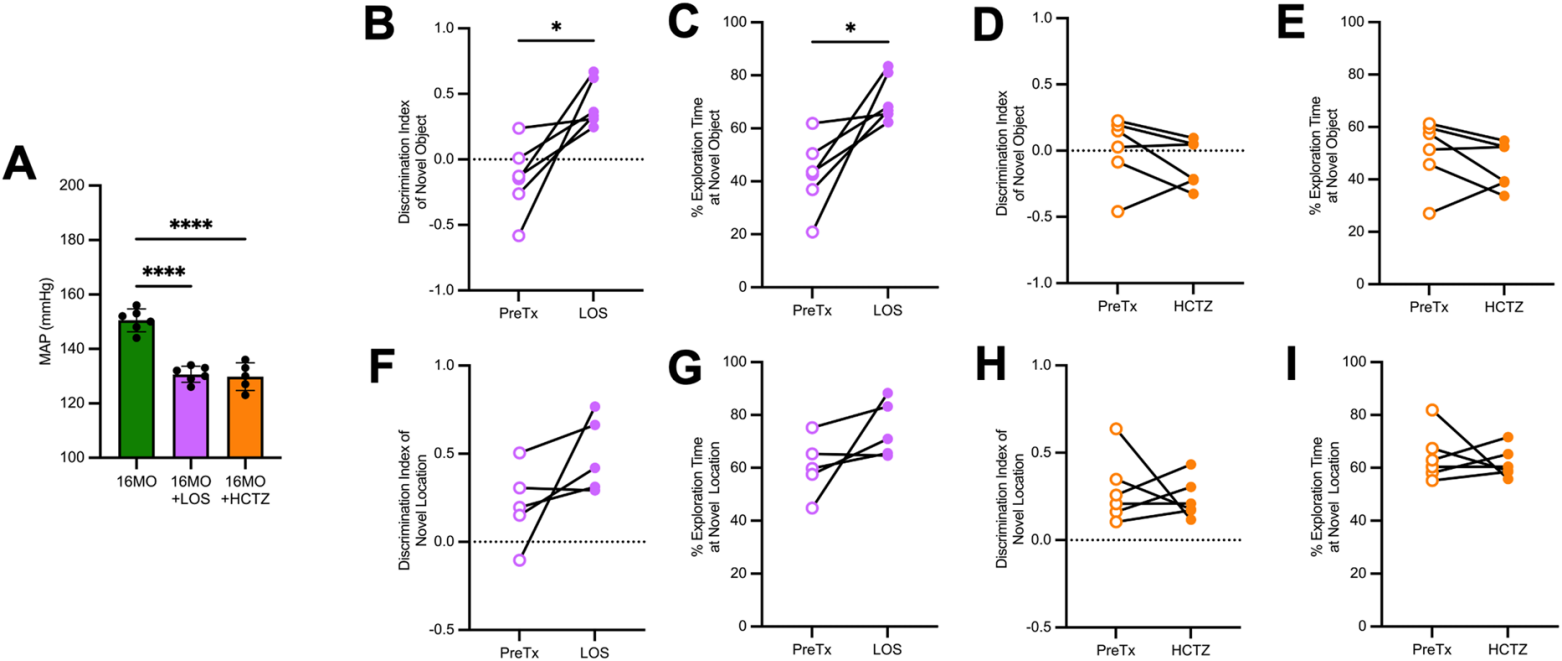
Losartan improves recognition memory in 16 month old male Sprague–Dawley rats with established hypertension. **A** Mean arterial pressure (MAP; mmHg) measured via acute instrumentation in male rats at 16 month old (MO) with no treatment, with losartan (LOS; 3 mg/kg/day s.c., 21-days), or with hydrochlorothiazide (HCTZ; 4 mg/kg/day s.c., 14-days); (*n* = 5–6/group), Data used for 16 month old male is also used in Fig. 1A. **B** Discrimination index of novel object recognition task performance in losartan treated rats before (PreTx) and after treatment (*n* = 6/group). **C** Percent of time spent at novel object in losartan treated rats before (PreTx) and after treatment (*n* = 6/group). **D** Discrimination index of novel object recognition task performance in hydrochlorothiazide treated rats before (PreTx) and after treatment (*n* = 6/group). **E** Percent of time spent at novel object in hydrochlorothiazide treated rats before (PreTx) and after treatment (*n* = 6/group). **F** Discrimination index of object location task performance in losartan treated rats before (PreTx) and after treatment (*n* = 6/group). **G** Percent of time spent at novel location in losartan treated rats before (PreTx) and after treatment (*n* = 6/group). **H** Discrimination index of object location task performance in hydrochlorothiazide treated rats before (PreTx) and after treatment (*n* = 6/group). **I** Percent of time spent at novel location in hydrochlorothiazide treated rats before (PreTx) and after treatment (*n* = 6/group). Statistical significance for A was determined using one-way ANOVA with a post-hoc Tukey test, ****p < 0.0001; statistical significance for B–I was determined using paired samples *t*-test, *p < 0.05

Losartan and hydrochlorothiazide lower blood pressure in aging male SD rats. To achieve blood pressure reduction, we administered 21-days of losartan or 14-days of hydrochlorothiazide. MAP was lowered to similar levels in both treatment groups (Fig. 5A). It should be noted that MAP data from 16-MO untreated male rats are the same values used in Fig. 1A.

Losartan improves recognition memory in aging male SD rats. Rats were tested on NOR task and OLT both prior to initiation of treatment and at the end of the treatment as a within subjects’ study design. On familiarization for both pre- and post-treatment, rats showed no preference toward either object on either the NOR or the OLT (Table 2). In rats treated with losartan, recognition memory significantly improved compared to pretreatment (Fig. 5B, C). However, losartan treatment did not improve spatial memory compared to pretreatment (Fig. 5F, G). Treatment with hydrochlorothiazide did not improve recognition memory or spatial memory compared to pretreatment (Fig. 5D, E, H, I).

**Table 2:** Familiarization from novel object recognition task and object location tasks in 16-month-old male Sprague–Dawley rats pre- and post-treatment with losartan or hydrochlorothiazide

Treatment		Novel object recognition task	Object location task
		Familiarization index	Familiarization index
Losartan	PreTx	0.181 ± 0.441	− 0.073 ± 0.054
	PostTx	0.207 ± 0.388	0.145 ± 0.176
Hydrochlorothiazide	PreTx	0.021 ± 0.119	0.064 ± 0.251
	PostTx	− 0.029 ± 0.220	− 0.061 ± 0.137

Familiarization index, defined as time spent at left object minus time spent at right object divided by time spent at both objects, calculated for familiarization of the novel object recognition and object location tasks in 16-month-old (MO) male Sprague–Dawley rats before treatment (Tx) and after Tx with either losartan (s.c. 3 mg/kg/day, 21 days) or hydrochlorothiazide (s.c. 4 mg/kg/day, 14 days). Data is presented as group mean ± standard deviation, *N* = 5–6/group

**Fig. 6:**
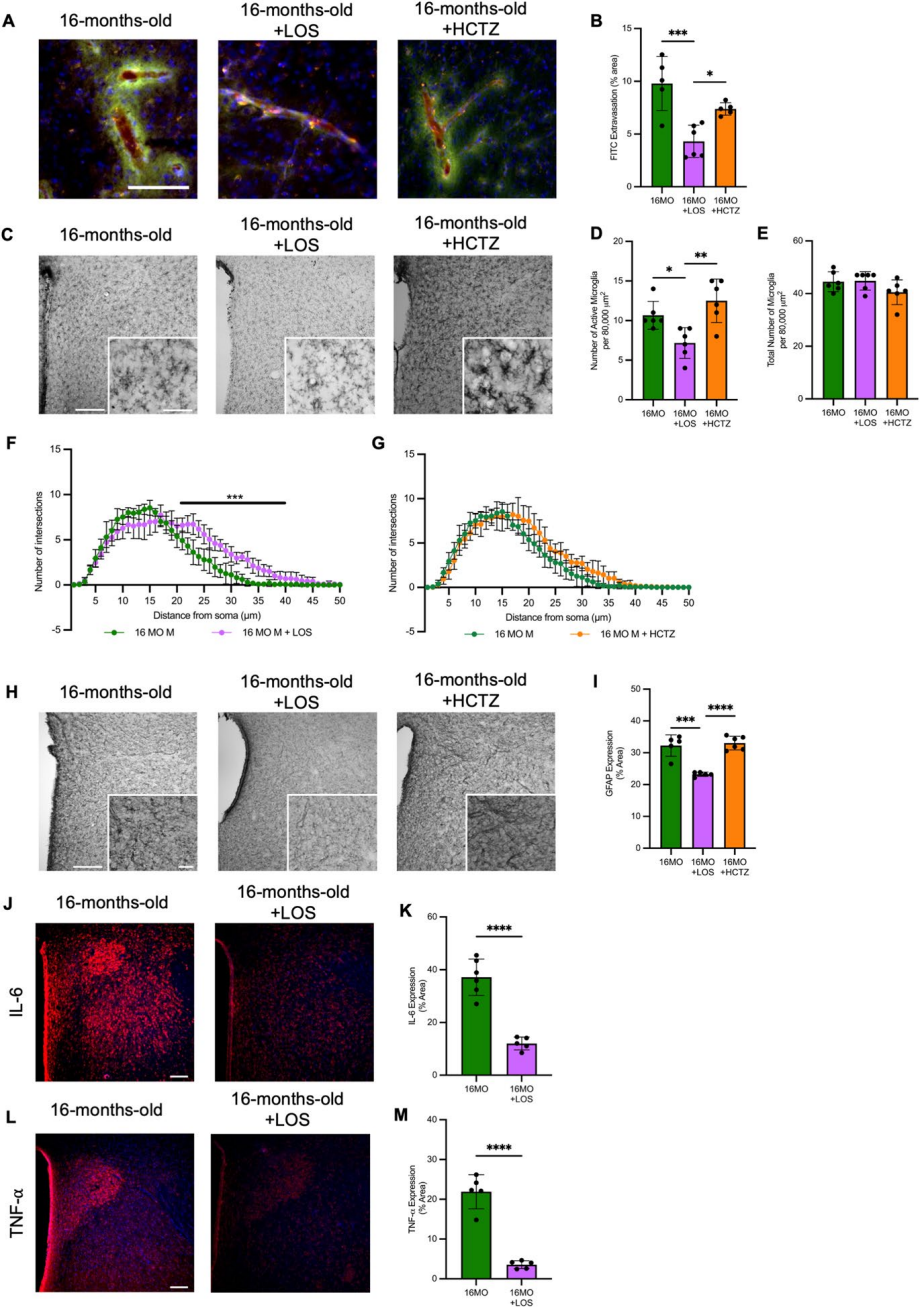
Losartan improves BBB disruption and neuroinflammation in the PVN of 16 month old male Sprague–Dawley rats with established hypertension. **A** Representative photomicrographs (captured at 10X magnification) from level 2 of the PVN (bregma − 1.72 mm to − 2.04 mm) of FITC extravasation as a measure of BBB disruption in male rats at 16-months-old (MO) with no treatment, with losartan (LOS; 3 mg/kg/day s.c., 21 days), or with hydrochlorothiazide (HCTZ; 4 mg/kg/day s.c., 14 days); Scale bar = 200 μm. Representative image for 16 month old male is also used in Fig. 2A. **B** FITC extravasation in level 2 of the PVN in male rats at 16 months old with no treatment, with losartan, or with hydrochlorothiazide (*n* = 5–6/group). Data used for 16 month old male is also used in Fig. 2B. **C** Representative photomicrographs (captured at 10X magnification with 40X magnification inset) from level 2 of the PVN (bregma − 1.72 to − 2.04 mm) of microglia (stained using OX-42/CD11b/c) in male rats at 16 months old with no treatment, with losartan, or with hydrochlorothiazide; scale bar (10X) = 200 μm; scale bar (40X) = 50 μm. Representative image for 16 month old male is also used in Fig. 2E. **D** Number of active microglia per 80,000 μm^2^ in level 2 of the PVN in male rats at 16-months-old with no treatment, with losartan, or with hydrochlorothiazide (*n* = 6/group). Data used for 16 month old male is also used in Fig. 2F. **E** Total number of microglia per 80,000 μm.^2^ in level 2 of the PVN in male rats at 16-months-old with no treatment, with losartan, or with hydrochlorothiazide (*n* = 6/group); Data used for 16 month old male is also used in Fig. 2G. **F** Sholl analysis expressed as number of intersections as a function of distance from the soma (μm) in 8 microglia per rat from level 2 of the PVN in 16- vs. 16 month old + losartan male (M) rats (*n* = 6/group); Data used for 16 month old male is also used in Fig. 2L, M. **G** Sholl analysis expressed as number of intersections as a function of distance from the soma (μm) in 8 microglia per rat from level 2 of the PVN in 16- vs. 16 month old + hydrochlorothiazide male (M) rats (*n* = 6/group); Data used for 16 month old male is also used in Fig. 2L, M. **H** Representative photomicrographs (captured at 10X magnification with 20X magnification inset) from level 2 of the PVN (bregma − 1.72 to − 2.04 mm) of astrocytes (stained using glial fibrillary acidic protein (GFAP)) in male rats at 16 months old with no treatment, with losartan, or with hydrochlorothiazide; scale bar (10X) = 200 μm; scale bar (20X) = 50 μm. Representative image for 16 month old male is also used in Fig. 2N. **I** GFAP expression presented as percent area of level 2 of the PVN in male rats at 16-months-old with no treatment, with losartan, or with hydrochlorothiazide (*n* = 6/group); data used for 16 month old male is also used in Fig. 2O. **J** Representative photomicrographs of interleukin-6 (IL-6) expression in level 2 of the PVN (bregma − 1.72 to − 2.04 mm) of male rats at 16 months old with no treatment or with losartan (*N* = 5–6/group). Scale bar (10X) = 200 μm; representative image for 16 month old male is also used in Fig. 3A. **K** IL-6 expression as percent area of the PVN in male Sprague–Dawley rats at 16-months-old with no treatment or with losartan (*N* = 5–6/group), Data used for 16 month old male is also used in Fig. 3B. **L** Representative photomicrographs of tumor necrosis factor-⍺ (TNF-⍺) expression in level 2 of the PVN (bregma − 1.72 to − 2.04 mm) of male rats at 16 months old with no treatment or with losartan (*N* = 5–6/group). Scale bar (10X) = 200 μm; representative image for 16 month old male is also used in Fig. 3E. **M** TNF-⍺ expression as percent area of the PVN in male Sprague–Dawley rats at 16-months-old with no treatment or with losartan (*N* = 5–6/group). Data used for 16 month old male is also used in Fig. 3F. Statistical significance was determined using one-way ANOVA with a post-hoc Tukey test (B, D, E, I), *p < 0.05, **p < 0.01, ***p < 0.005, and ****p < 0.0001. For Sholl analysis and cytokine expression (F, G, K, M), Student’s *t*-test was used to determine statistical significance, ***p < 0.005 and ****p < 0.0001

Losartan attenuates BBB disruption and neuroinflammation in aging male SD rats. It should be noted that data and representative images for 16MO male rats are the same as those used in Fig. 2 and 3. LOS treatment decreased BBB permeability (Fig. 6A, B, Supplemental Fig. 13), attenuated the number of active microglia in the PVN, without changing the total number of microglia (Fig. 6C–E, Supplemental Fig. 14), increased branching complexity (Fig. 6F), and decreased GFAP expression (Fig. 6H, I, Supplemental Fig. 15). HCTZ showed no changes in any of these parameters compared to untreated 16-month-old male rats (Fig. 6A–E, G, H, I, Supplemental Figs. 13–15). Because no changes were detected in HCTZ treated rats, cytokine expression was only measured in LOS treated rats. Here, LOS was found to attenuate both IL-6 and TNF-⍺ expression compared to untreated rats (Fig. 6J–M, Supplemental Figs. 16&17).

## Discussion

These studies were designed to investigate the hypothesis that (1) BBB disruption and neuroinflammation in the PVN contributes to age-dependent hypertension in SD rats, (2) age-dependent hypertension in SD rats is associated with cognitive impairment, and (3) the use of antihypertensive therapeutics to lower blood pressure in aged SD rats with established hypertension improves cognitive function. These studies reveal that normal aging evokes hypertension and enhanced sympathetic outflow that is associated with PVN BBB disruption and neuroinflammation in male but not female SD rats. Aged hypertensive male rats have impairments in recognition and spatial memory. The AT1R antagonist LOS proved to have pleiotropic benefits, improving recognition memory while attenuating PVN neuroinflammation and BBB disruption and lowering blood pressure in aged hypertensive male rats. This same effect was not seen following HCTZ treatment, which only lowered blood pressure. These findings add to the growing body of literature implicating hypertension in the development of cognitive impairment and support AT1R blockers as a treatment for cognitive impairment in age-dependent hypertension.

In this study, we observed male but not female SD rats develop age-dependent hypertension with enhanced sympathetic tone [5] modeling human hypertension in aging populations [6]. While our prior findings reported an increase in blood pressure with age in male rats, in the groups of rats utilized in these studies, we observed a greater spread in blood pressure values. Additionally, while our prior study identified a difference in global sympathetic tone between 3 and 16MO male rats, we found a progressive increase in global sympathetic tone between all age groups. These findings suggest, as observed in human populations, there is variability in the development of hypertension in the aging SD rat. Taken together, along with data indicating an increase in sympathetic outflow to the vasculature with age, these findings suggest hypertension is driven in part by alterations in sympathetic nervous system activity in aged male SD rats.

In aging male rats, we found an increase in FITC extravasation in the PVN, indicative of BBB disruption. This occurred along with neuroinflammation indicated by an increase in active microglia, reactive astrocytes, and increased expression of proinflammatory cytokines IL-6 and TNF-⍺. Prior studies report an increase in BBB disruption in Spontaneously Hypertensive Rats [8, 26], with one study suggesting this allows circulating factors such as ANG II to gain access to the PVN from the circulation and induce neuroinflammation mediated by microglia [8]. Moreover, prior studies in hypertensive animal models (i.e., G⍺i_2_ salt sensitive rats and ANG II infusion rats) found microglial activation and proinflammatory cytokine production in the PVN was associated with enhanced sympathetic tone and elevated blood pressure [10, 11]. Further, minocycline, an anti-inflammatory antibiotic, reduced microglial activation and neuroinflammation in the PVN, modulated sympathetic tone and decreased blood pressure in multiple rodent models of hypertension that exhibit neuroinflammation [9–11]. Interestingly, direct microinjections of proinflammatory cytokines TNF-⍺ or IL-1β into the PVN increased blood pressure, renal sympathetic nerve activity, and enhanced the cardiac sympathetic afferent reflex [27]. Further, pretreatment with TNF-⍺ and IL-1β in the PVN sensitized rats to developing sympathoexcitation and hypertension in response to a subpressor dose of ANG II [27]. This suggests a relationship between proinflammatory cytokines and elevated blood pressure and that proinflammatory cytokines in the PVN can predispose a rat to developing hypertension. While we did find an increase in both IL-6 and TNF-⍺, we did not determine if they are gaining access to the PVN due to the disrupted BBB or if they are being produced within the PVN by microglia or other inflammatory cells. While studies of obesity identified astrocyte reactivity in multiple brain regions [28], including the PVN, our study appears to be the first to find an increase in GFAP, indicative of astrocyte reactivity, in the PVN of hypertensive rats [11]. We speculate that astrocyte reactivity may contribute to enhanced sympathetic outflow seen in our model as a prior study found an inhibition in glutamate transport by astrocytes in the PVN leads to activation of glutamate receptors on presympathetic neurons increasing activity and driving sympathoexcitation [29]. Collectively our findings suggest PVN BBB disruption and neuroinflammation is contributing significantly to increased sympathetic outflow and blood pressure in aging male rats.

Our data reveal impairments in performance on the NOR and OLT, indicative of impairments in recognition and spatial memory in aged hypertensive male rats. This suggests an association between increased blood pressure and cognitive impairment with age, which is also seen in human studies such as SEARCH-Health and MEMENTO [12, 13]. Our studies continue to support an association between hypertension and cognitive impairment which is also seen in young hypertensive rodents (i.e., ANG II infused mice and Dahl Salt Sensitive rats) [17, 18]. Those studies further revealed hypertension-related cognitive impairment is associated with neurovascular dysfunction, including BBB disruption, microbleeds, and neuroinflammation in the hippocampus and cortex [17, 18]. Thus, we speculate these may be mechanisms mediating impairments in cognitive function in aging SD rats that can be further investigated in future studies.

In opposition to human hypertension, our female rats showed no age-related increase in blood pressure and sympathetic tone, which is consistent with our previous findings [5], and no evidence of BBB disruption, neuroinflammation, or cognitive impairment. While humans experience a significant loss of female sex steroids during menopause, female SD rats enter persistent estrous following cycle cessation between 8 and 12 MO [30]. This is supported by our findings that female rats maintained plasma estradiol and progesterone concentrations with age. The difference in plasma estrogen at 8MO may be the result of changes in cycles. As we did not complete vaginal cytology, we cannot confirm this. Our studies emphasize persistent sex differences in hypertension that are consistent with our prior findings and other rodent models of hypertension [5, 31, 32]. Estrogen augments tight junction function limiting BBB disruption [33] and activation of estrogen receptors on microglia and astrocytes is known to prevent neuroinflammation [34, 35]. Further, loss of estrogen impairs cognition [36]. Accordingly, the maintenance of estrogen throughout the lifespan may mediate neuroprotection in our aging female rats and supports the future exploration of female sex steroid contributions in age-dependent hypertension.

We next tested the hypothesis that lowering blood pressure can improve cognitive function in rats with established hypertension. Both LOS, an AT1R antagonist, and HCTZ, a thiazide diuretic, lowered blood pressure to the same magnitude. Our results revealed losartan improved recognition memory to the performance observed in 3 and 8MO male rats. LOS, however, did not improve spatial memory and hydrochlorothiazide had no impact on cognitive function. This lack of improvement in spatial memory was surprising given that other studies in rodents with hypertension (young Dahl Salt Sensitive rats) [18] and with Alzheimer’s disease [37] found spatial memory improves following AT1R blockade at doses that do not alter blood pressure. The use of different behavioral tasks to assess spatial memory in those studies compared to the presented study may be responsible for the discrepancies between findings. Our findings suggest the cognitive benefits of an AT1R blockade may be independent of blood pressure lowering mechanisms. In human studies, including SPRINT-MIND, HYVET, and PROGRESS, blood pressure reduction, regardless of drug class, significantly decreased risk of developing cognitive impairment and dementia [38–40]. This is in contrast to other studies, like CALIBREX, which showed favorable cognitive benefits in the use of AT1R antagonists over thiazide diuretics and ACE inhibitors [41, 42]. In combination with our findings and others in rodent models and human subjects, we speculate drug class, specifically AT1R blockers, may be important for the treatment of hypertension-related cognitive dysfunction.

Our studies also revealed LOS attenuated BBB disruption and diminished neuroinflammation in the PVN of aged male rats to levels seen in young normotensive male rats. Prior studies indicate AT1R blockade in the Spontaneously Hypertensive Rat improved PVN BBB dysfunction and neuroinflammation leading to a reduction in sympathetic outflow and lower blood pressure [8, 24], suggesting AT1R activation contributes to BBB disruption and neuroinflammation. This is further supported by improvements in blood brain barrier disruption and neuroinflammation by AT1R blockade in regions outside of the PVN, like the hippocampus and cortex in hypertensive models [18, 43]. A potential mechanism that we speculate underlies these improvements in BBB function and the anti-inflammatory impact of LOS may be a result of the blockade of AT1R on endothelial cells, astrocytes, microglia, and neurons [29, 43]. Supporting this is the reported anti-inflammatory effect of AT1R antagonists in Alzheimer’s Disease and traumatic brain injury [37, 44]. Although we did not address this in this study, we speculate there may be neuroinflammation in regions associated with learning and memory, such as the hippocampus, which could modulate cognition and be improved following LOS treatment. Taken together, we propose the aging SD rat as a model of hypertension-associated cognitive impairment and these findings suggest LOS provides unique neurocognitive and neuroimmune benefits compared to HCTZ, particularly in established hypertension.

Our hypothesis was that lowering blood pressure would improve cognitive function in aged male SD rats with established hypertension. Drug doses and durations of administration were selected as they have previously been shown to effectively lower blood pressure [5, 19]. Both compounds are first-line antihypertensive therapeutics. LOS is one of many AT1R blockers, a class which includes Telmisartan and Olmesartan, used to treat blood pressure. Multiple studies have revealed beneficial off-target effects of AT1R blockers including anti-inflammatory effects [8, 24]. Further, telmisartan has been shown to cross the BBB while LOS may not cross the BBB as readily [45]. However, we elected to use LOS in these studies as it was previously found to effectively improve BBB permeability and neuroinflammation in the PVN in Spontaneously Hypertensive rats [8]. Given that aging male SD rats have increased BBB permeability, we speculate that losartan may gain access to the brain parenchyma as a result, contributing to a reduction in neuroinflammation and improvements in cognitive function seen in these studies.

### Study limitations

First, blood pressure was measured using acute instrumentation rather than radiotelemetry. While radiotelemetry is recognized as the gold standard of blood pressure measurements in rodents, our previous findings in aging SD rats identified the same trend and magnitude of blood pressure increases with age when measured using telemetry vs. acute instrumentation [5]. We acknowledge there are other regions in the brain that may also modulate sympathetic outflow and they may contribute to age-related changes in the regulation of blood pressure. However, we limited our studies to investigating the PVN’s contributions to age-dependent hypertension due to its central role in sympathetic outflow and blood pressure regulation. The current studies only used two cognitive assessments and therefore only assessed recognition and spatial memory. These tasks were utilized as they involved minimal training for the animal and do not evoke profound changes in blood pressure due to stress or movement in order to complete the task. In the future, more behavioral assessments including the Barnes Maze will be utilized to further assess different cognitive domains. Further studies are necessary to better understand the underlying contributions of the hypertension-related changes in the hippocampus and its impact on cognition. It should be noted that anesthesia can increase the permeability of the BBB, which may explain a small amount of FITC extravasation in normotensive male and female rats. Ketamine/xylazine anesthesia was used in these studies as it has previously been reported to limit BBB permeability compared to other anesthetics.

## Perspectives

**Fig. 7:**
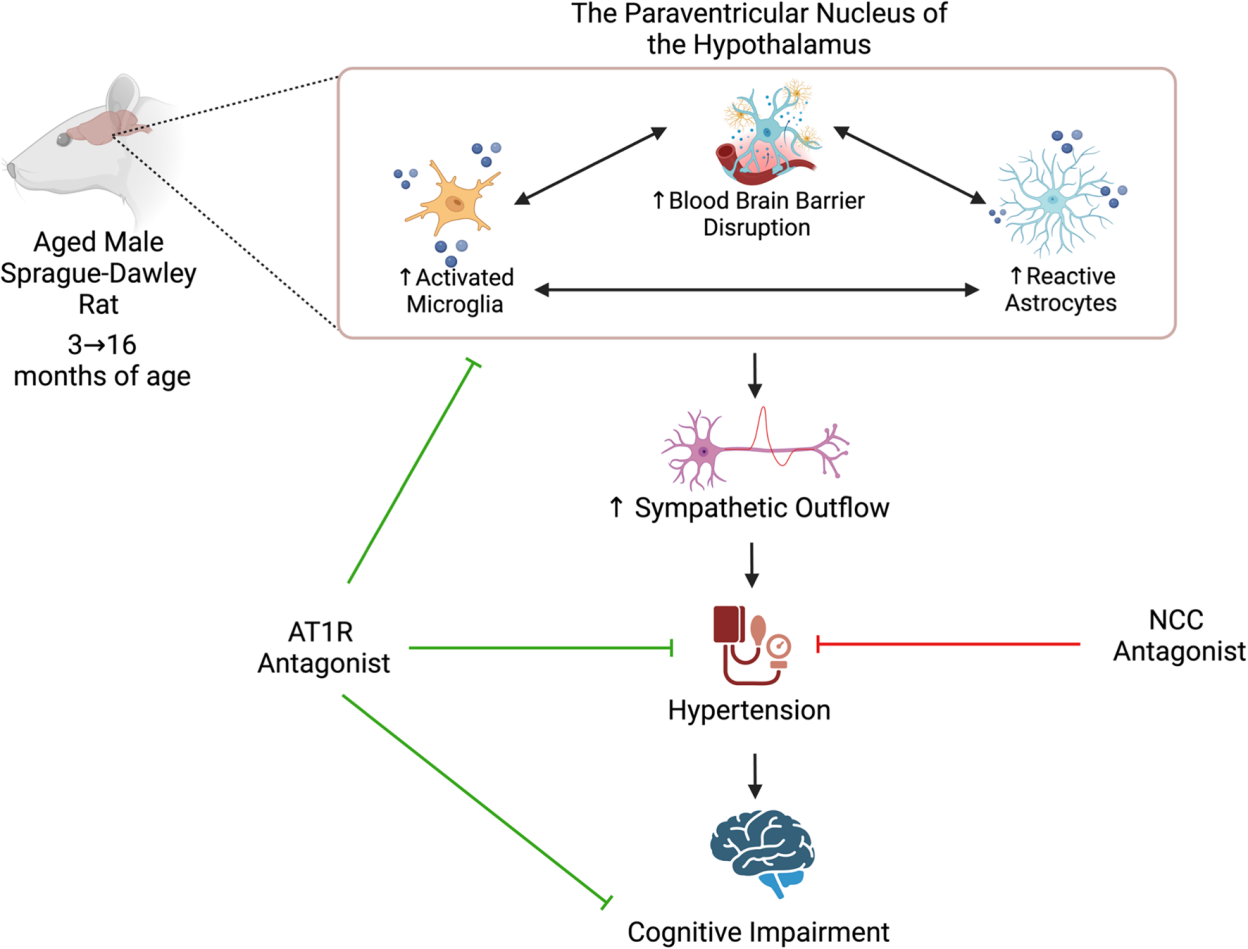
Schematic of proposed hypothesis. BBB disruption and neuroinflammation in the paraventricular nucleus (PVN) contributes to increased sympathetic outflow and hypertension in aged male Sprague–Dawley rats. Age-dependent hypertension is associated with increased cognitive impairment. While hydrochlorothiazide, an NCC antagonist, only lowers blood pressure, losartan, an AT1R lowers blood pressure, attenuates PVN neuroinflammation and improves cognitive function in aged male rats

This study is the first to establish that BBB disruption and neuroinflammation in the PVN contributes to age-dependent hypertension in a sex-dependent manner and that age-dependent hypertension is associated with cognitive impairment in the SD rat. However, while lifelong elevations in blood pressure contribute to cognitive impairment, attenuating hypertension-associated cognitive impairment is more nuanced that just lowering blood pressure as LOS provides pleiotropic benefits independent of its blood pressure lowering abilities. Our findings suggest not all antihypertensive medications are equal in improving cognitive performance or attenuating neuroinflammation, even when blood pressure is effectively lowered (Fig. 7). While our results support the use of AT1R antagonists to improve cognitive function in hypertensive individuals, we speculate based on our findings and others that the use of an AT1R blockade at a dose that does not impact blood pressure in normotensive individuals or individuals with elevated blood pressure may enhance cognitive outcomes, particularly in the aging population.

## Supplementary Information

Below is the link to the electronic supplementary material.

Supplementary file1 (PDF 10907 KB)

Supplementary file2 (DOCX 131 KB)

## Data Availability

All data is available upon reasonable request.

## Abbreviations

ANG II: Angiotensin II
AT1R: Angiotensin II type 1 receptor
BBB: Blood brain barrier
FITC: Fluorescein isothiocyanate-Dextran
GFAP: Glial fibrillary acidic protein
HCTZ: Hydrochlorothiazide
IL-6: Interleukin 6
i.p.: Intraperitoneal
i.v.: Intravenous
LOS: Losartan
MAP: Mean arterial pressure
MO: Months old
NE: Norepinephrine
NOR: Novel object recognition test
OLT: Object location test
PBS: Phosphate-buffered saline
PFA: Paraformaldehyde
PVN: Paraventricular nucleus of the hypothalamus
SD: Sprague-Dawley
TNF-⍺: Tumor necrosis factor-⍺
