# Supplementary material for: Losartan attenuates sex-dependent hypertension, neuroinflammation, and cognitive impairment in the aging male sprague–dawley rat
Source: GeroScience. 2024 Dec 3;47(3):3007–26. doi: 10.1007/s11357-024-01409-4 (PMC12181475; doi:10.1007/s11357-024-01409-4)
Supplement: Supplementary file 1 — Supplementary file1 (PDF 10907 KB) [file 11357_2024_1409_MOESM1_ESM.pdf]

ONLINE SUPPLEMENTAL MATERIALS

Title: Losartan attenuates sex-dependent hypertension, neuroinflammation, and cognitive impairment in the aging male Sprague-Dawley rat

Authors: Kayla M. Nist, M.S.[1,2], Hannah Bard, B.S.[2,3], Brannon McBride, B.S.[2,3], Angela L. Capriglione, B.S.[1,2], Jesse D. Moreira, Ph.D., M.S.[2,4], David H. Farb, Ph.D.[3,5], Richard D. Wainford, Ph.D.[2,3,6]

Affiliations:

1. Department of Anatomy and Neurobiology, Boston University Chobanian and Avedisian School of Medicine, Boston, MA, USA
2. Whitaker Cardiovascular Institute, Department of Medicine, Boston University Chobanian and Avedisian School of Medicine, Boston, MA, USA
3. Department of Pharmacology & Experimental Therapeutics, Boston University Chobanian and Avedisian School of Medicine, Boston, MA, USA
4. Department of Health Sciences, Sargent College, Boston University, Boston, MA
5. Center for Systems Neuroscience, Boston University, Boston, MA
6. Department of Medicine, Division of Cardiology, Emory University School of Medicine, Atlanta, GA, USA

Short Title: Aging, Hypertension, and Cognitive Impairment

Corresponding Author: Richard D. Wainford, BSc, Ph.D., F.A.H.A., Professor, Emory University School of Medicine, Division of Cardiology, 1750 Haygood Drive, N220, Atlanta, Georgia 30322. Phone: 404-727-3754 Fax: 404-713-0070; E-mail: rwainfo@emory.edu

Key words: aging, hypertension, neuroinflammation, cognitive impairment, angiotensin II type 1 receptor

Supplemental Table

Supplemental Table 1: Primary antibody information for immunohistochemistry and immunofluorescence.

Antibody	Catalog Number	Manufacturer	Species	Concentration
CD11 b/c (OX-42)	554859	BD Biosciences	mouse	1:100
GFAP	MAB360	Millipore-Sigma	mouse	1:5000
IL-6 (C12-1-hIL-6)	SC-32296	Santa Cruz Biotechnologies	mouse	1:250
TNF-α (52B83)	SC-52746	Santa Cruz Biotechnologies	mouse	1:100

Antibody information including catalog number, manufacturer, species, and concentrations used.

Supplemental Figures

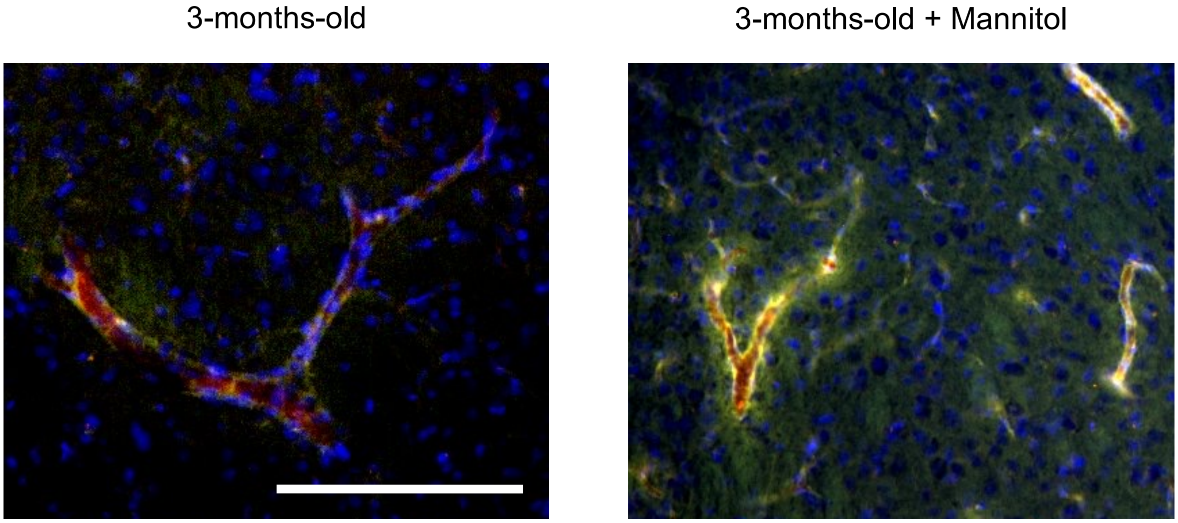

Supplemental Figure 1: Hyperosmotic mannitol disrupts the blood brain barrier. Representative photomicrographs from male rats at 3-months-old with no treatment or treated with an intracarotid bolus of mannitol (1.4M) prior to receiving a co-infusion of FITC (10 kDa, green) and RHO (70 kDa, red). This procedure was performed to show proof of concept that FITC extravasates into the brain parenchyma when the blood brain barrier is disrupted. Tissue was counter stained with DAPI (nuclei, blue). Images were enlarged from 10X magnification photomicrographs; Scale bar: 100 µm.

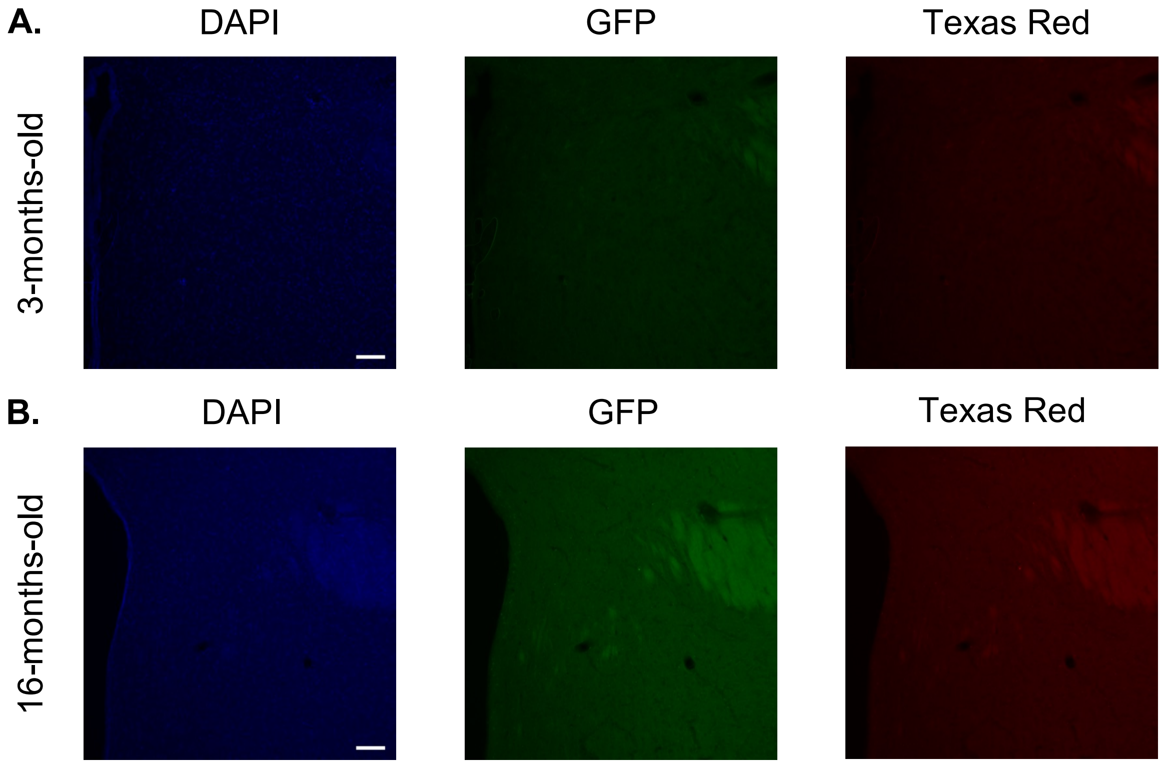

Supplemental Figure 2: Representative fluorescence control images from untreated tissue. (A) Unprocessed PVN tissue from a male rat at 3-months-old that was counterstained with DAPI in Prolong Diamond Mountant and imaged with DAPI, GFP, and Texas Red filters on a Keyence BZ-9000 to show a lack of autofluorescence in unprocessed PVN tissue. **(B)** Unprocessed PVN tissue sampled from a male rat at 16-months-old that was counterstained with DAPI in Prolong Diamond Mountant and imaged with DAPI, GFP, and Texas Red filters to show a lack of autofluorescence in PVN tissue. All images were taken at 10X magnification. Scale bar = 100 µm.

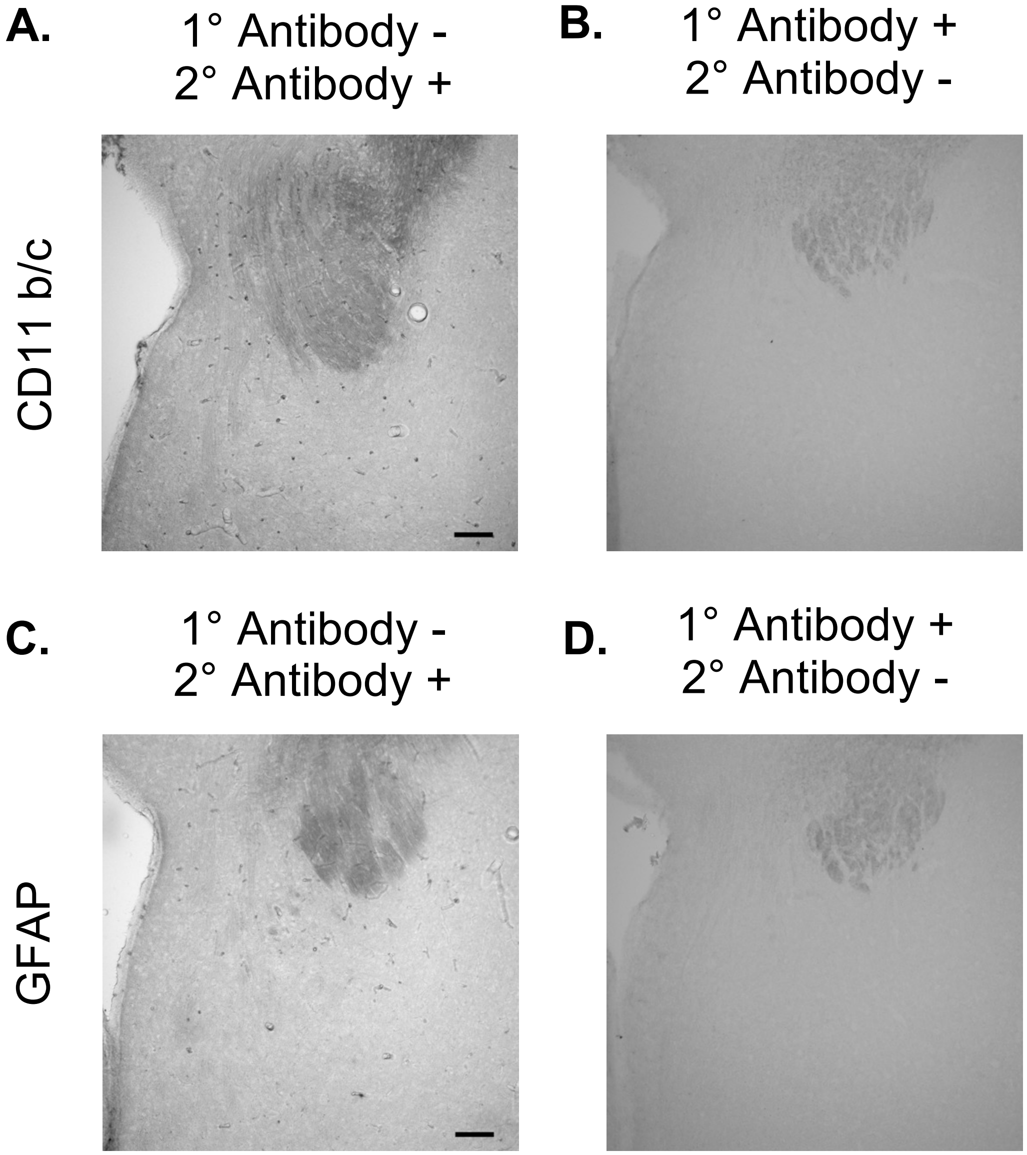

Supplemental Figure 3: Representative images from negative controls for CD11 b/c and GFAP immunohistochemistry. (A) Negative antibody control image for CD11 b/c- primary antibody excluded, secondary antibody included in sample. **(B)** Negative antibody control image for CD11 b/c- primary antibody included, secondary antibody excluded in sample. **(C)** Negative antibody control image for GFAP- primary antibody excluded, secondary antibody included in sample. **(D)** Negative antibody control image for GFAP- primary antibody included, secondary antibody excluded in sample. All images were taken in the PVN at 10X magnification. Scale bar = 100 μm.

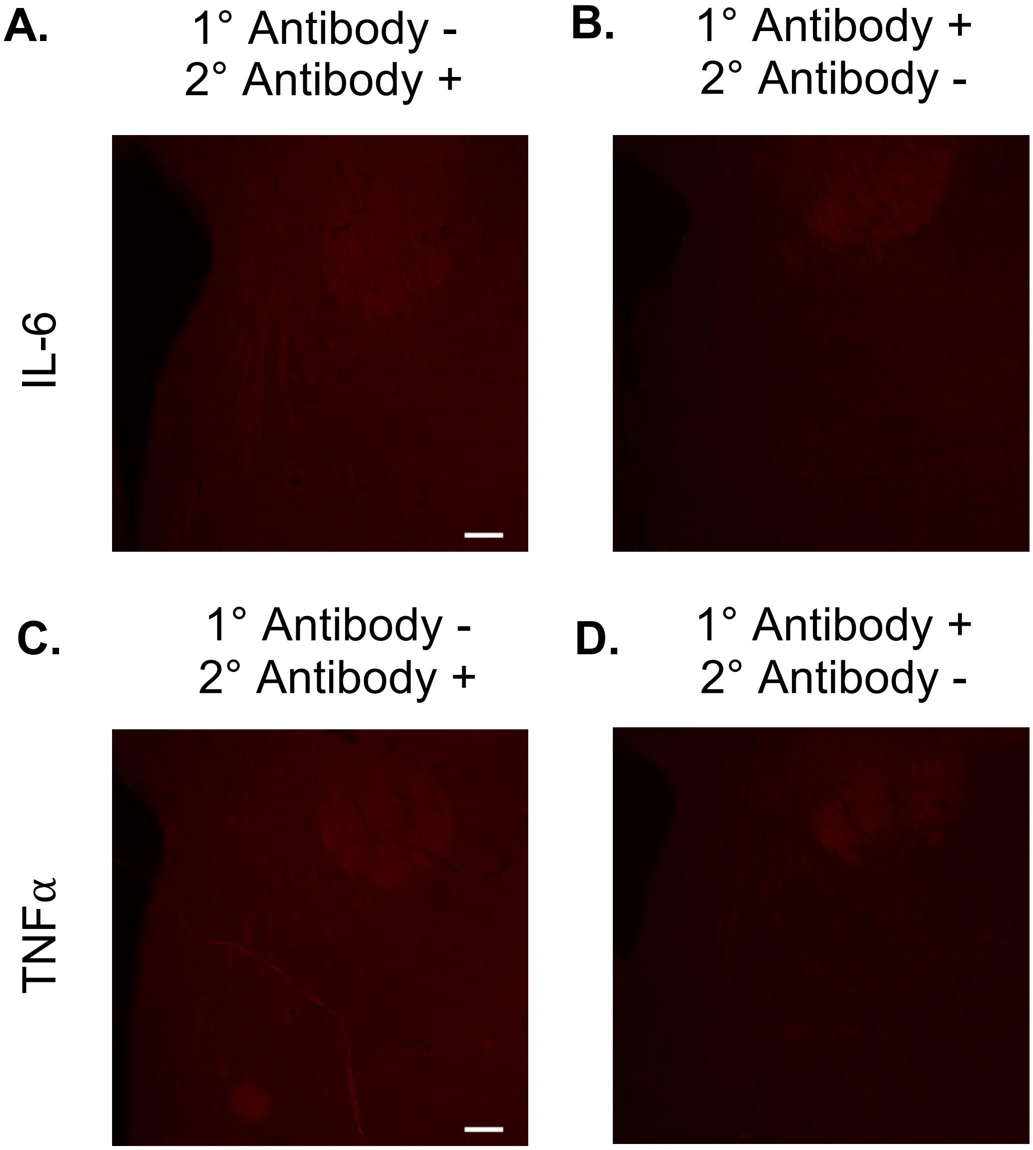

Supplemental Figure 4: Representative images from negative controls for IL-6 and TNFα immunofluorescence. (A) Negative antibody control image for IL-6- primary antibody excluded, secondary antibody included in sample. **(B)** Negative antibody control image for IL-6- primary antibody included, secondary antibody excluded in sample. **(C)** Negative antibody control image for TNFα- primary antibody excluded, secondary antibody included in sample. **(D)** Negative antibody control image for TNFα- primary antibody included, secondary antibody excluded in sample. All images were taken in the PVN at 10X magnification. Scale bar = 100 μm.

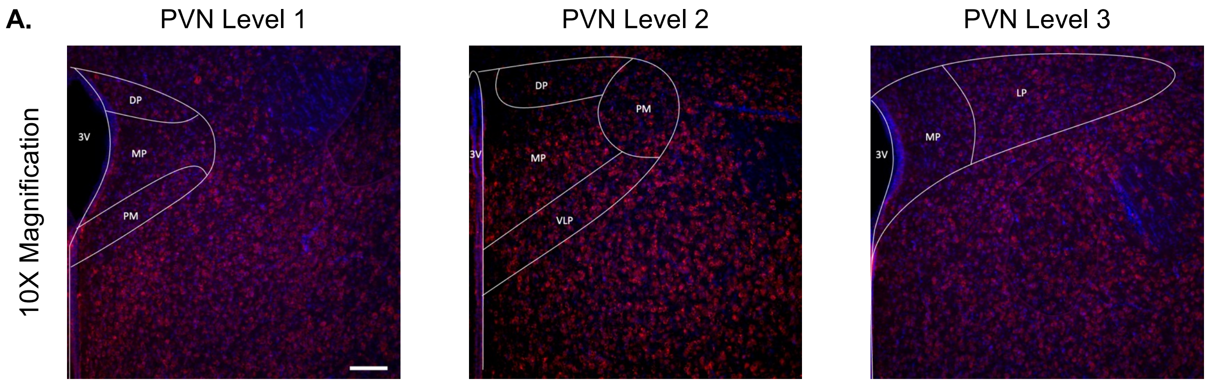

Supplemental Figure 5: Representative outline of PVN anatomy at 10X and 20X magnification. (A) Representative photomicrographs at 10X magnification with PVN neuroanatomy for level 1 (Bregma -1.44 mm to -1.72 mm), level 2 (Bregma -1.72 mm to -2.04 mm), and level 3 (Bregma -2.04 mm to -2.28 mm); Anatomy overlayed on photomicrographs of IL-6 in 3-month-old male rats; Scale bar = 200 µm. 3V = third ventricle, DP = dorsal parvocellular, MP = medial parvocellular, PM = posterior magnocellular, VLP = ventrolateral parvocellular, LP = lateral parvocellular.

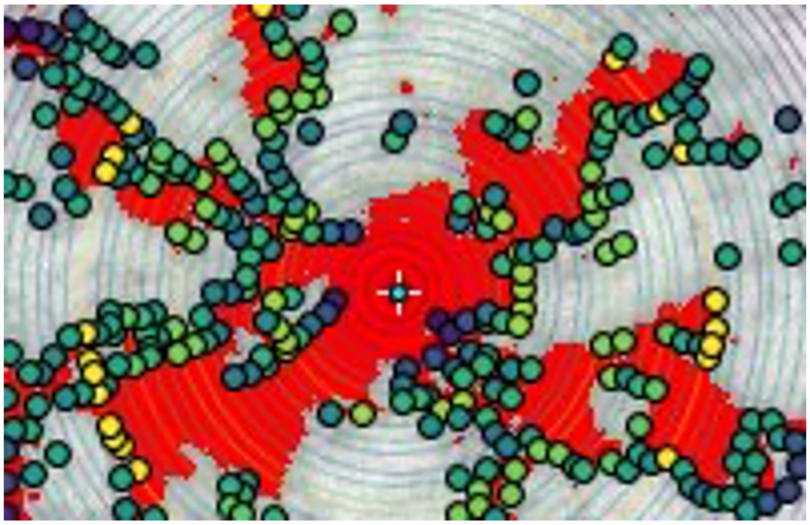

Supplemental Figure 6: Representative image of Sholl analysis of a microglia. Representative image of a microglia following completion of Sholl analysis performed using FIJI/ImageJ. Concentric rings are 1 µm apart. Dots represent an intersection with the concentric ring and was counted by FIJI/ImageJ.

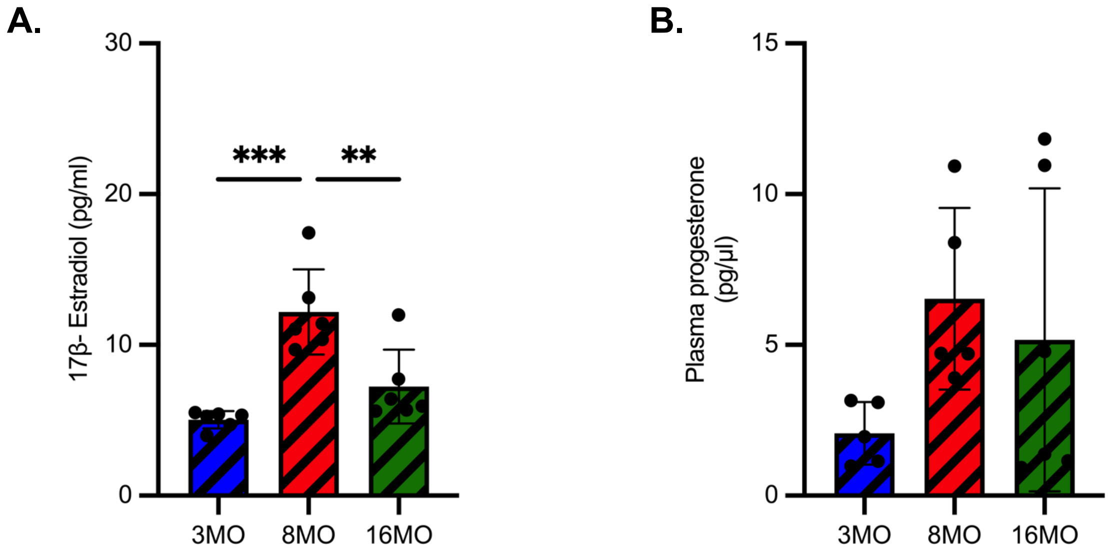

Supplemental Figure 7: Plasma estradiol and progesterone in aging female Sprague-Dawley rats. (A) Plasma 17β-estradiol (pg/ml) measured via ELISA in female rats at 3-, 8-, and 16-months-old (MO) (N=6/group). **(B)** Plasma progesterone (pg/µl) measured via liquid chromatography-mass spectrometry in female rats at 3, 8-, and 16-months-old (N=5-6/group). Statistical significance determined using one-way ANOVA with a post-hoc Tukey's test; $^{**}p<0.01$, $^{***}p<0.005$.

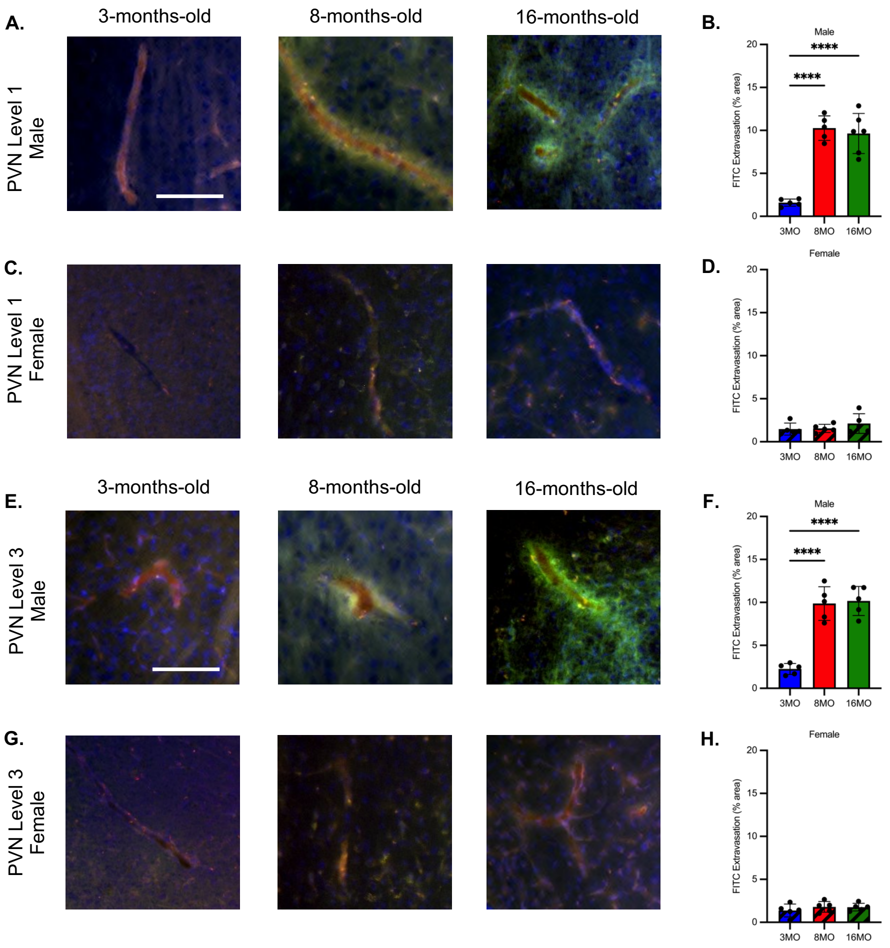

Supplemental Figure 8: Blood brain barrier disruption in level 1 and level 3 of the PVN in aging male and female Sprague-Dawley rats. (A) Representative photomicrographs (captured at 10X magnification) from level 1 of the PVN (Bregma -1.44 mm to -1.72 mm) of FITC extravasation in the PVN as a measure of blood brain barrier permeability in male rats at 3-, 8-, and 16-months-old (MO); Scale bar (10X) = 100 µm. **(B)** FITC extravasation presented as percent area of level 1 of the PVN in aging male rats (n=5-6/group). **(C)** Representative photomicrographs (captured at 10X magnification) from level 1 of the PVN (Bregma -1.44 to -1.72) of FITC extravasation in the PVN as a measure of blood brain barrier permeability in female rats at 3-, 8-, and 16-months-old; Scale bars shown in (A). **(D)** FITC extravasation presented as percent area of level 1 of the PVN in aging female rats (n=5-6/group). **(E)** Representative photomicrographs (captured at 10X magnification) from level 3 of the PVN (Bregma -2.04 mm to -2.28 mm) of FITC extravasation in the PVN as a measure of blood brain barrier permeability in male rats at 3-, 8-, and 16-months-old; Scale bar (10X) = 100 µm. **(F)** FITC extravasation presented as percent area of level 3 of the PVN in aging male rats (n=5-6/group). **(G)** Representative photomicrographs (captured at 10X magnification) from level 3 of the PVN (Bregma -2.04 to -2.28) of FITC extravasation in the PVN as a measure of blood brain barrier permeability in female rats at 3-, 8-, and 16-months-old; Scale bars shown in (**E**). **(H)** FITC extravasation presented as percent area of level 3 of the PVN in aging female rats (n=5-6/group). Statistical significance was determined using one-way ANOVA with a post-hoc Tukey test, ****p<0.0001.

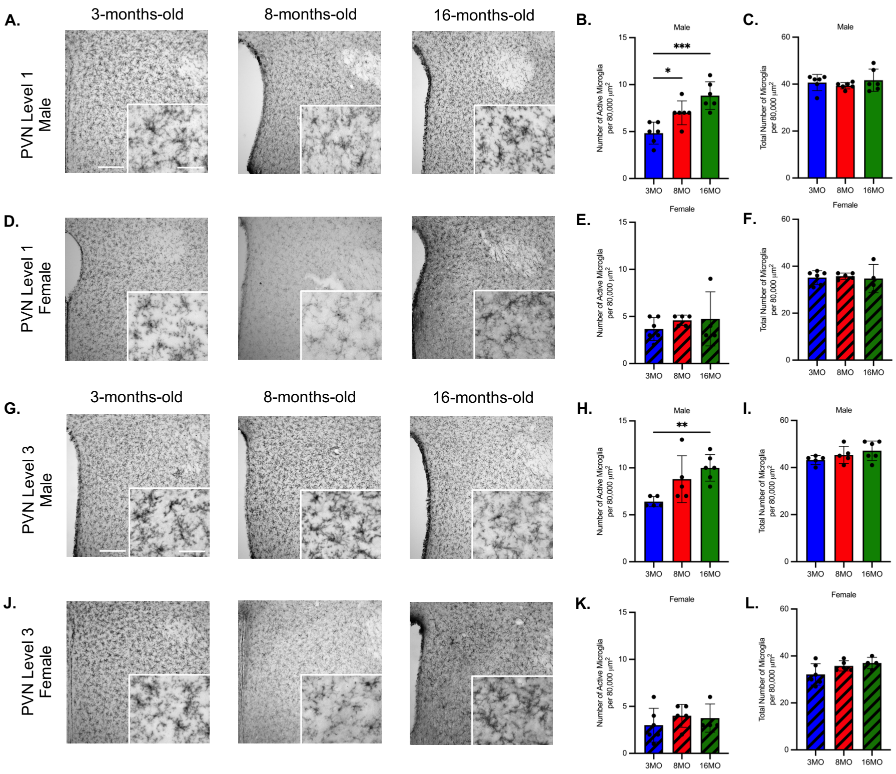

Supplemental Figure 9: Microglia expression in level 1 and level 3 of the PVN in aging male and female Sprague-Dawley rats. (A) Representative photomicrographs (captured at 10X magnification with 40X magnification inset) from level 1 of the PVN (Bregma -1.44 mm to -1.72 mm) of microglia (stained using CD11b/c (OX-42)) in male rats at 3-, 8-, and 16-months-old (MO); Scale bar (10X) = 200 µm, Scale bar (40X) = 50 µm. **(B)** Number of active microglia per 80,000 μm^2 in level 1 of the PVN in aging male rats (n=6/group). **(C)** Total number of microglia per 80,000 μm^2 in level 1 of the PVN in aging male rats; (n=6/group). **(D)** Representative photomicrographs (captured at 10X magnification with 40X magnification inset) from level 1 of the PVN (Bregma -1.44 mm to -1.72 mm) of microglia (stained using CD11b/c (OX-42)) in female rats at 3-, 8-, and 16-months-old; Scale bar shown in (A). **(E)** Number of active microglia per 80,000 μm^2 in level 1 of the PVN in aging female rats (n=4-6/group). **(F)** Total number of microglia per 80,000 μm^2 in level 1 of the PVN in aging female rats (n=4-6/group). **(G)** Representative photomicrographs (captured at 10X magnification with 40X magnification inset) from level 3 of the PVN (Bregma -2.04 mm to -2.28 mm) of microglia (stained using CD11b/c (OX-42)) in male rats at 3-, 8-, and 16-months-old; Scale bar (10X) = 200 µm, Scale bar (40X) = 50 µm. **(H)** Number of active microglia per 80,000 μm^2 in level 3 of the PVN in aging male rats (n=5-6/group). **(I)** Total number of microglia per 80,000 μm^2 in level 3 of the PVN in in aging male rats (n=6/group). **(J)** Representative photomicrographs (captured at 10X magnification with 40X magnification inset) from level 3 of the PVN (Bregma -2.04 mm to -2.28 mm) of microglia (stained using CD11b/c (OX-42)) in female rats at 3-, 8-, and 16-months-old; Scale bar shown in (G). **(K)** Number of active microglia per 80,000 μm^2 in level 3 of the PVN in aging female rats (n=4-6/group). **(L)** Total number of microglia per 80,000 μm^2 in level 3 of the PVN in aging female rats (n=4-6/group). Statistical significance was determined using one-way ANOVA with a post-hoc Tukey test, $^*p<0.05$, $^{**}p<0.01$, $^{***}p<0.005$.

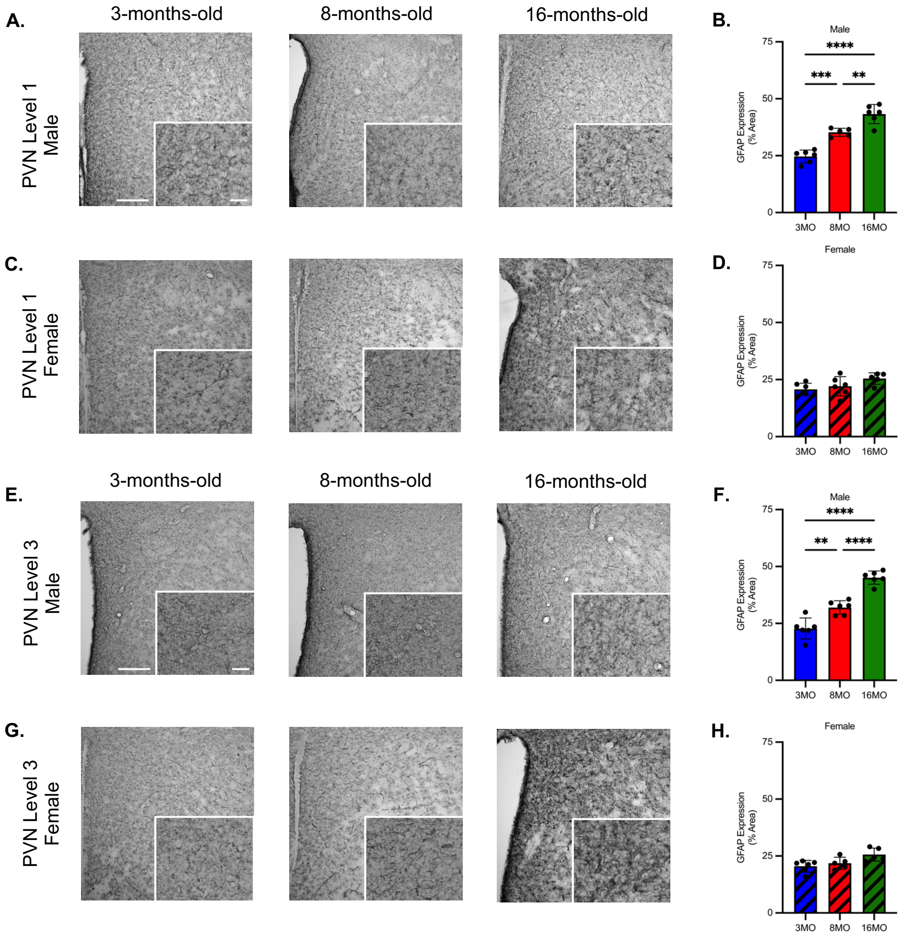

Supplemental Figure 10: Astrocyte expression in level 1 and level 3 of the PVN in aging male and female Sprague-Dawley rats. (A) Representative photomicrographs (captured at 10X magnification with 20X magnification inset) from level 1 of the PVN (Bregma -1.44 mm to -1.72 mm) of astrocytes (stained using glial fibrillary acidic protein (GFAP)) in male rats at 3-, 8-, and 16-months-old (MO); Scale bar (10X) = 200 µm, Scale bar (20X) = 50 µm. **(B)** GFAP expression presented as percent area of level 1 of the PVN in aging male rats (n=5-6/group). **(C)** Representative photomicrographs (captured at 10X magnification with 20X magnification inset) from level 1 of the PVN (Bregma -1.44 to -1.72) of astrocytes (stained using GFAP) in female rats at 3-, 8-, and 16-months-old; Scale bars shown in (A). **(D)** GFAP expression presented as percent area of level 1 of the PVN in aging female rats (n=5-6/group). **(E)** Representative photomicrographs (captured at 10X magnification with 20X magnification inset) from level 3 of the PVN (Bregma -2.04 mm to -2.28 mm) of astrocytes (stained using glial fibrillary acidic protein (GFAP)) in male rats at 3-, 8-, and 16-months-old; Scale bar (10X) = 200 µm, Scale bar (20X) = 50 µm. **(F)** GFAP expression presented as percent area of level 3 of the PVN in aging male rats (n=5-6/group). **(G)** Representative photomicrographs (captured at 10X magnification with 20X magnification inset) from level 3 of the PVN (Bregma -2.04 to -2.28) of astrocytes (stained using GFAP) in female rats at 3-, 8-, and 16-months-old; Scale bars shown in **(E)**. **(H)** GFAP expression presented as percent area of level 3 of the PVN in aging female rats (n=5-6/group). Statistical significance was determined using one-way ANOVA with a post-hoc Tukey test, $^{**}p<0.01$, $^{***}p<0.005$, $^{****}p<0.0001$.

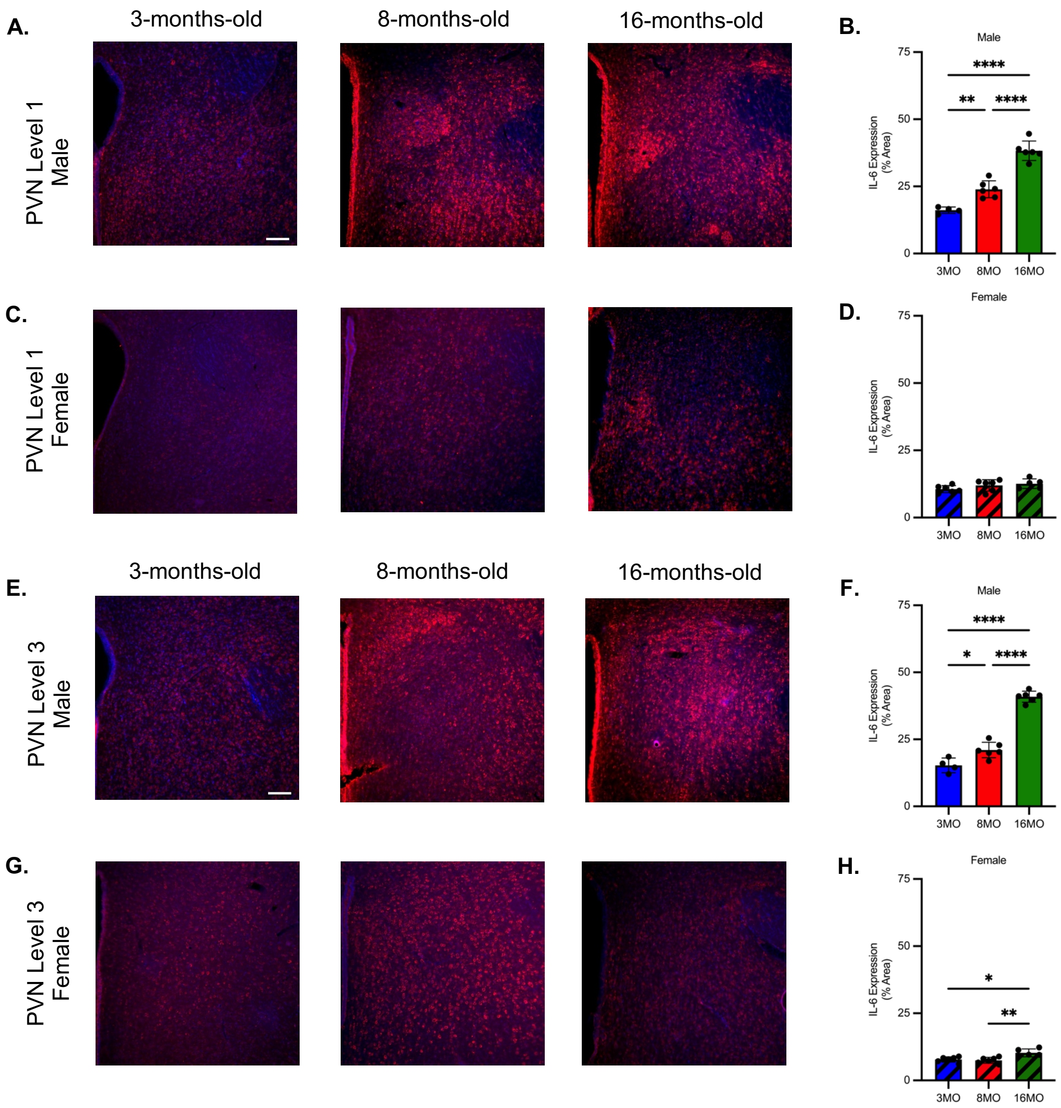

Supplemental Figure 11: IL-6 expression in level 1 and level 3 of the PVN in aging male and female Sprague-Dawley rats. (A) Representative photomicrographs (captured at 10X magnification) from level 1 of the PVN (Bregma -1.44 mm to -1.72 mm) of interlukin-6 (IL-6) in male rats at 3-, 8-, and 16-months-old (MO); Scale bar (10X) = 200 µm. **(B)** IL-6 expression presented as percent area of level 1 of the PVN in aging male rats (n=4-6/group). **(C)** Representative photomicrographs (captured at 10X magnification) from level 1 of the PVN (Bregma -1.44 to -1.72) of IL-6 in female rats at 3-, 8-, and 16-months-old; Scale bar shown in (A). **(D)** IL-6 expression presented as percent area of level 1 of the PVN in aging female rats (n=5-6/group). © Representative photomicrographs (captured at 10X magnification) from level 3 of the PVN (Bregma -2.04 mm to -2.28 mm) of IL- 6 in male rats at 3-, 8-, and 16-months-old; Scale bar (10X) = 200 µm. **(F)** IL-6 expression presented as percent area of level 3 of the PVN in aging male rats (n=4-6/group). **(G)** Representative photomicrographs (captured at 10X magnification) from level 3 of the PVN (Bregma -2.04 to -2.28) of IL-6 in female rats at 3-, 8-, and 16-months-old; Scale bars shown in **(E)**. **(H)** IL-6 expression presented as percent area of level 3 of the PVN in aging female rats (n=5-6/group). Statistical significance was determined using one-way ANOVA with a post-hoc Tukey test, *p<0.05, **p<0.01, ****p<0.0001.

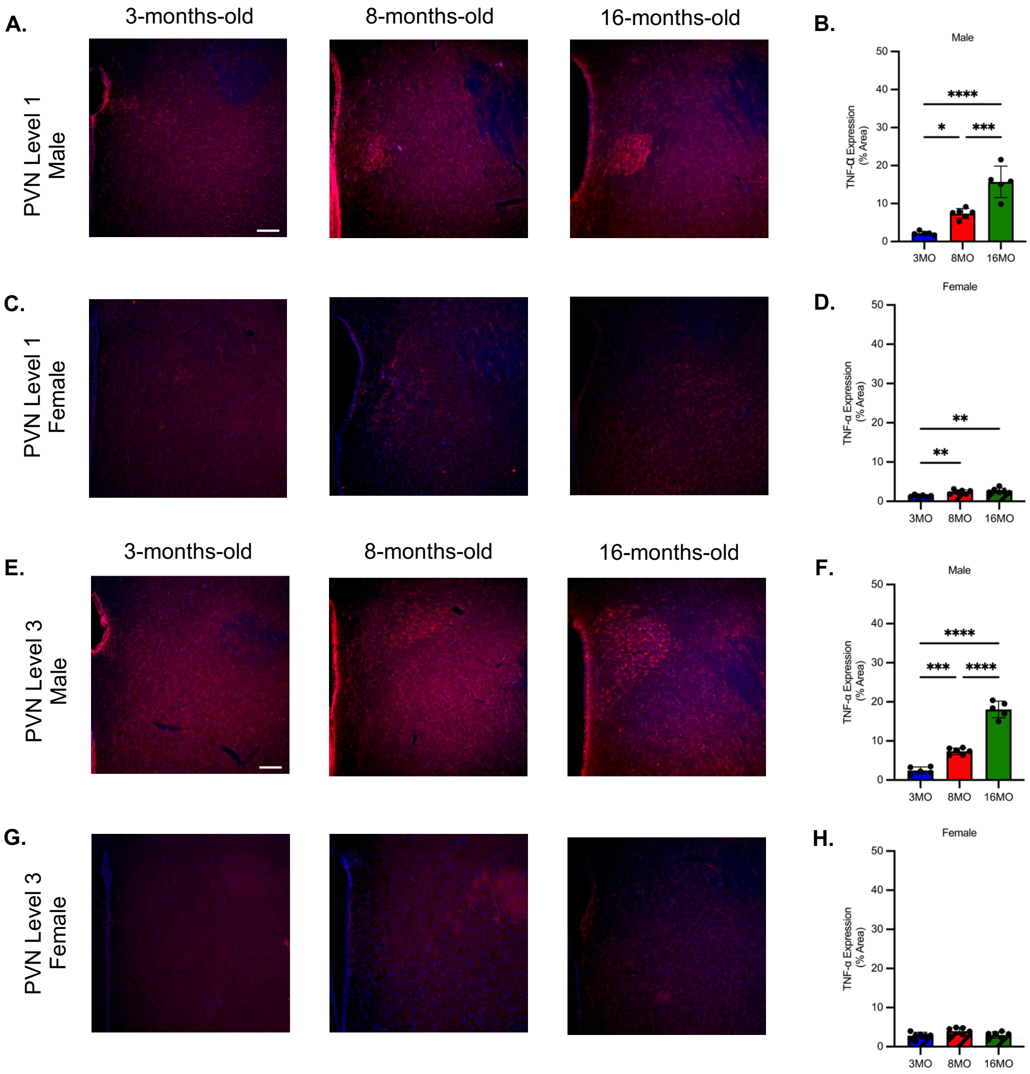

Supplemental Figure 12: TNF-α expression in level 1 and level 3 of the PVN in aging male and female Sprague-Dawley rats. (A) Representative photomicrographs (captured at 10X magnification) from level 1 of the PVN (Bregma -1.44 mm to -1.72 mm) of tumor necrosis factor-alpha (TNF-α) in male rats at 3-, 8-, and 16-months-old (MO); Scale bar (10X) = 200 μm. **(B)** TNF-α expression presented as percent area of level 1 of the PVN in aging male rats (n=5-6/group). **(C)** Representative photomicrographs (captured at 10X magnification) from level 1 of the PVN (Bregma -1.44 to -1.72) of TNF-α in female rats at 3-, 8-, and 16-months-old, Scale bar shown in (A). **(D)** TNF-α expression presented as percent area of level 1 of the PVN in aging female rats (n=6/group). **(E)** Representative photomicrographs (captured at 10X magnification) from level 3 of the PVN (Bregma -2.04 mm to -2.28 mm) of TNF-α in male rats at 3-, 8-, and 16-months-old; Scale bar (10X) = 200 μm. **(F)** TNF-α expression presented as percent area of level 3 of the PVN in aging male rats (n=4-6/group). **(G)** Representative photomicrographs (captured at 10X magnification) from level 3 of the PVN (Bregma -2.04 to -2.28) of TNF-α in female rats at 3-, 8-, and 16-months-old; Scale bars shown in **(E)**. **(H)** TNF-α expression presented as percent area of level 3 of the PVN in aging female rats (n=6/group). Statistical significance was determined using one-way ANOVA with a post-hoc Tukey test, *p<0.05, **p<0.01, ***p<0.005, ****p<0.0001.

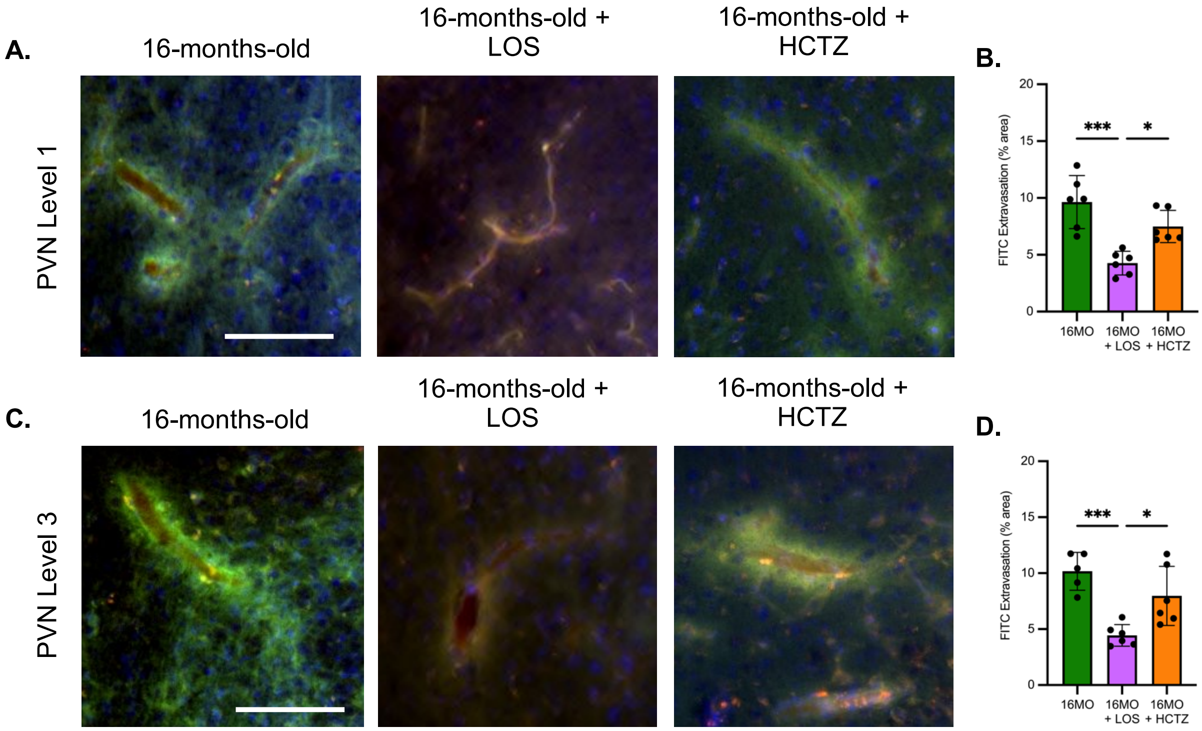

Supplemental Figure 13: Blood brain barrier disruption in PVN level 1 and 3 in aging male Sprague-Dawley rats treated with losartan or hydrochlorothiazide. (A) Representative photomicrographs (captured at 10X magnification) from level 1 of the PVN (Bregma -1.44 mm to -1.72 mm) of FITC extravasation in the PVN as a measure of blood brain barrier permeability in male rats at 16-months-old (MO) with no treatment, with losartan (LOS; 3 mg/kg/day s.c., 21-days), or with hydrochlorothiazide (HCTZ; 4 mg/kg/day s.c., 14-days); Scale bar (10X) = 100 µm. Representative image for 16-month-old male is also used in Supplemental Figure 4A. **(B)** FITC extravasation presented as percent area in level 1 of the PVN in male rats at 16-months-old with no treatment, with losartan, or with hydrochlorothiazide (n=6/group), Data used for 16-month-old male is also used in Supplemental Figure 4B. **(C)** Representative photomicrographs (captured at 10X magnification) from level 3 of the PVN (Bregma -2.04 mm to -2.28 mm) of FITC extravasation in the PVN as a measure of blood brain barrier permeability in male rats at 16-months-old with no treatment, with losartan, or with hydrochlorothiazide; Scale bar (10X) = 100 µm. Representative image for 16-month-old male is also used in Supplemental Figure 4E. **(D)** FITC extravasation presented as percent area in level 3 of the PVN in male rats at 16-months-old with no treatment, with losartan, or with hydrochlorothiazide (n=5-6/group), Data used for 16-month-old male is also used in Supplemental Figure 7F. Statistical significance was determined using one-way ANOVA with a post-hoc Tukey test, $^{*}p<0.05$, $^{***}p<0.005$.

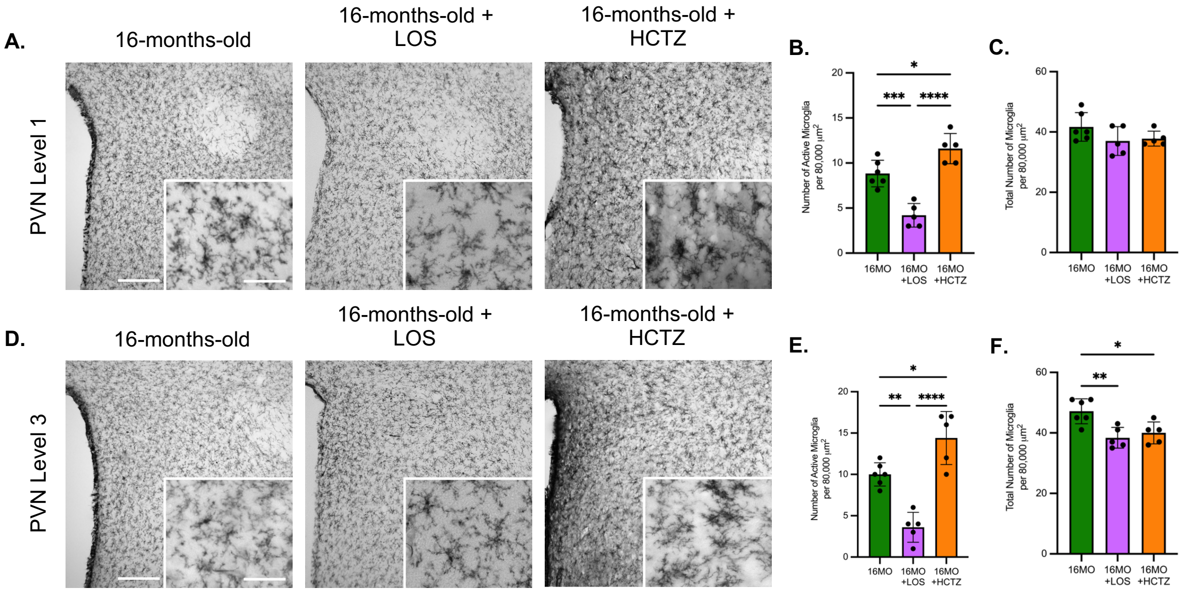

Supplemental Figure 14: Microglia in PVN level 1 and 3 in aging male Sprague-Dawley rats treated with losartan or hydrochlorothiazide. (A) Representative photomicrographs (captured at 10X magnification with a 40x insert) from level 1 of the PVN (Bregma -1.44 mm to -1.72 mm) of microglia (stained using CD11b/c (OX-42)) in male rats at 16-months-old (MO) with no treatment, with losartan (LOS; 3 mg/kg/day s.c., 21-days), or with hydrochlorothiazide (HCTZ; 4 mg/kg/day s.c., 14-days); Scale bar (10X) = 200 µm, Scale bar (40X) = 50 µm. Representative image for 16-month-old male is also used in Supplemental Figure 6A. **(B)** Number of active microglia per 80,000 μm^2 in level 1 of the PVN in male rats at 16-months-old with no treatment, with losartan, or with hydrochlorothiazide; (n=5-6/group), Data used for 16-month-old male is also used in Supplemental Figure 6B. **(C)** Total number of microglia per 80,000 μm^2 in level 1 of the PVN in male rats at 16-months-old with no treatment, with losartan, or with hydrochlorothiazide; (N=5-6/group), Data used for 16-month-old male is also used in Supplemental Figure 6C. **(D)** Representative photomicrographs (captured at 10X magnification with a 40x insert) from level 3 of the PVN (Bregma -2.04 mm to -2.28 mm) of microglia (stained using CD11b/c (OX-42)) in male rats at 16-months-old with no treatment, with losartan, or with hydrochlorothiazide; Scale bar (10X) = 200 µm, Scale bar (40X) = 50 µm. Representative image for 16-month-old male is also used in Supplemental Figure 6G. © Number of active microglia per 80,000 μm^2 in level 3 of the PVN in male rats at 16-months-old with no treatment, with losartan, or with hydrochlorothiazide; (n=5-6/group), Data used for 16-month-old male is also used in Supplemental Figure 6H. **(F)** Total number of microglia per 80,000 μm^2 in level 3 of the PVN in male rats at 16-months-old with no treatment, with losartan, or with hydrochlorothiazide; (n=5-6/group) Data used for 16-month-old male is also used in Supplemental Figure 6I. Statistical significance was determined using one-way ANOVA with a post-hoc Tukey test, *p<0.05, **p<0.01, ***p<0.005, ****p<0.0001.

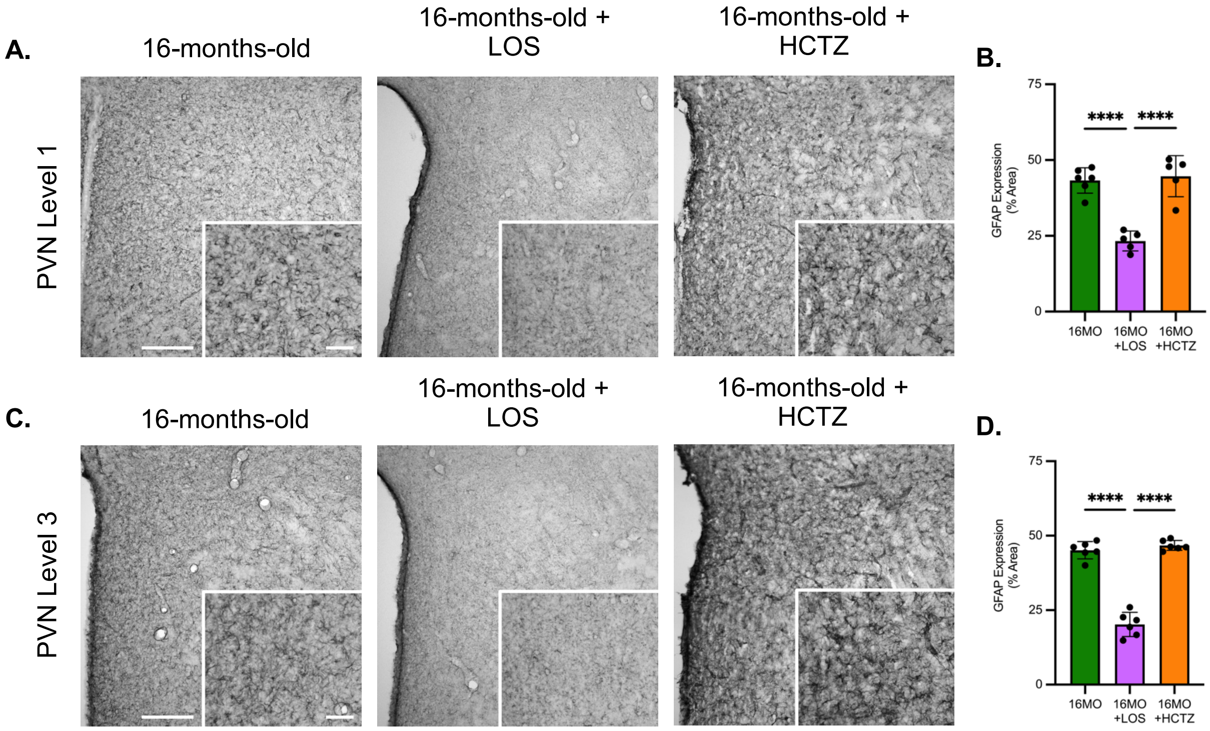

Supplemental Figure 15: Astrocytes in PVN level 1 and 3 in aging male Sprague-Dawley rats treated with losartan or hydrochlorothiazide. (A) Representative photomicrographs (captured at 10X magnification with a 20x insert) from level 1 of the PVN (Bregma -1.44 mm to -1.72 mm) of astrocytes (stained using glial fibrillary acidic protein (GFAP)) in male rats at 16-months-old (MO) with no treatment, with losartan (LOS; 3 mg/kg/day s.c., 21-days), or with hydrochlorothiazide (HCTZ; 4 mg/kg/day s.c., 14-days); Scale bar (10X) = 200 µm, Scale bar (20X) = 50 µm. Representative image for 16-month-old male is also used in Supplemental Figure 7A. **(B)** GFAP expression presented as percent area in level 1 of the PVN in male rats at 16-months-old with no treatment, with losartan, or with hydrochlorothiazide; (n=5-6/group), Data used for 16-month-old male is also used in Supplemental Figure 7B. **(C)** Representative photomicrographs (captured at 10X magnification with a 20x insert) from level 3 of the PVN (Bregma -2.04 mm to -2.28 mm) of astrocytes (stained using glial fibrillary acidic protein (GFAP in male rats at 16-months-old with no treatment, with losartan, or with hydrochlorothiazide; Scale bar (10X) = 200 µm, Scale bar (20X) = 50 µm. Representative image for 16-month-old male is also used in Supplemental Figure 7E. **(D)** GFAP expression presented as percent area in level 3 of the PVN in male rats at 16-months-old with no treatment, with losartan, or with hydrochlorothiazide; (n=6/group), Data used for 16-month-old male is also used in Supplemental Figure 7F. Statistical significance was determined using one-way ANOVA with a post-hoc Tukey test, ****$p<0.0001$.

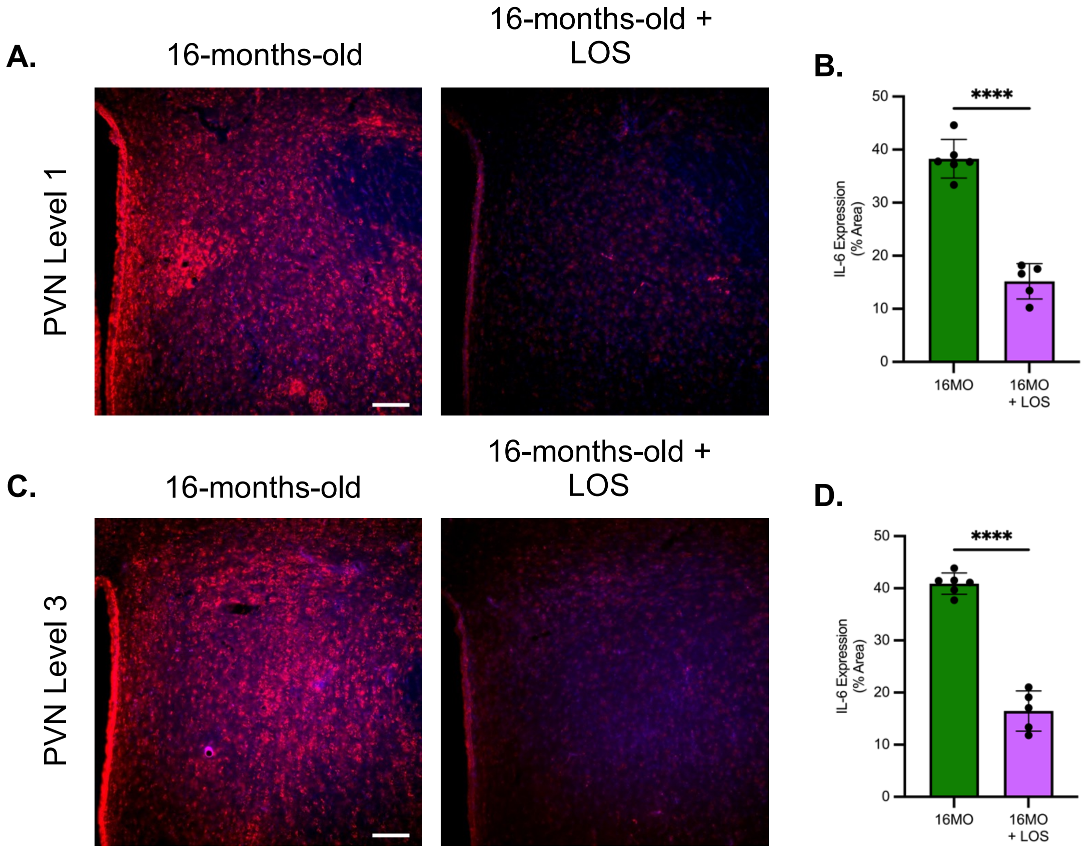

Supplemental Figure 16: IL-6 expression in PVN level 1 and 3 in aging male Sprague-Dawley rats treated with losartan or hydrochlorothiazide. (A) Representative photomicrographs (captured at 10X magnification) from level 1 of the PVN (Bregma -1.44 mm to -1.72 mm) of interlukin-6 (IL-6) in male rats at 16-months-old (MO) with no treatment, with losartan (LOS; 3 mg/kg/day s.c., 21-days); Scale bar (10X) = 200 µm. Representative image for 16-month-old male is also used ©n Supplemental Figure 8A. **(B)** IL-6 expression presented as percent area in level 1 of the PVN in male rats at 16-months-old with no treatment or with losartan; (n=5-6/group) Data used for 16-month-old male is also used in Supplemental Figure 8B. **(C)** Representative photomicrographs (captured at 10X magnification) from level 3 of the PVN (Bregma -2.04 mm to -2.28 mm) of IL-6 in male rats at 16-months-old with no treatment or with losartan; Scale bar (10X) = 200 µm. Representative image for 16-month-old male is also used in Supplemental Figure 8E. **(D)** IL-6 expression presented as percent area in level 3 of the PVN in male rats at 16-months-old with no treatment or with losartan (n=5-6/group) Data used for 16-month-old male is also used in Supplemental Figure 8F. Statistical significance was determined using student's T-test, ****p<0.0001.

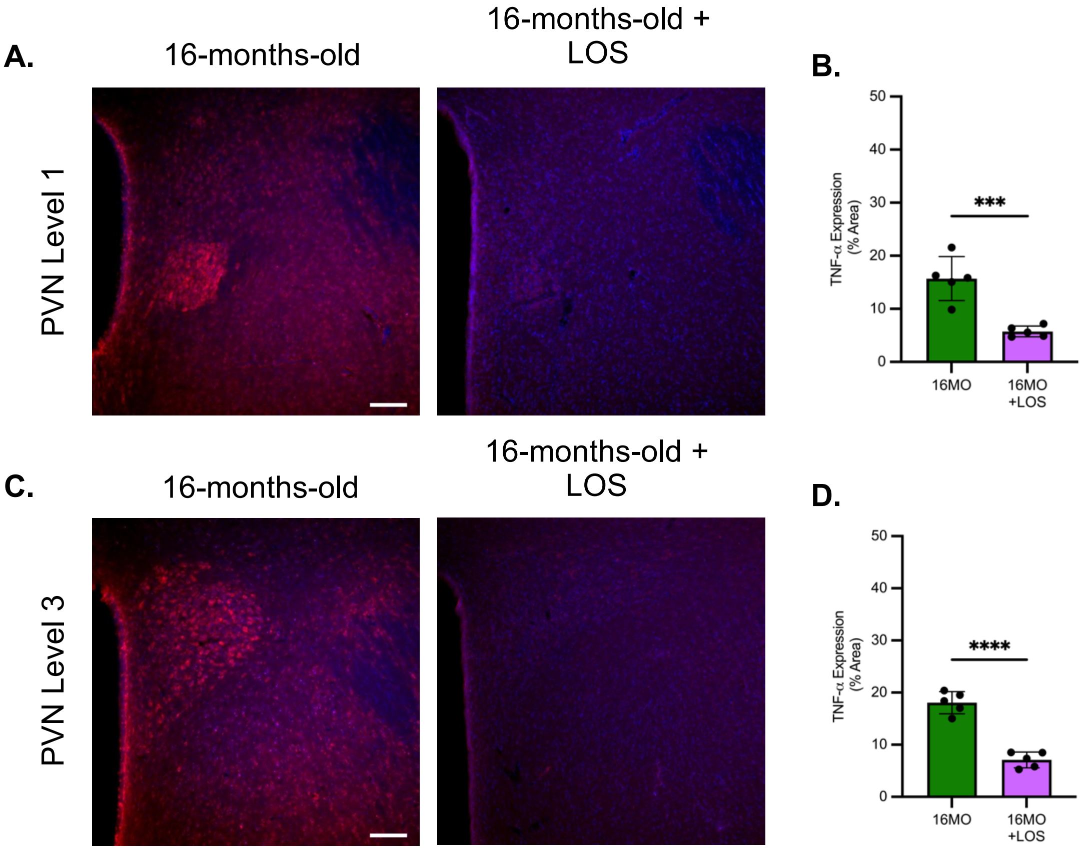

Supplemental Figure 17: TNF-α expression in PVN level 1 and 3 in aging male Sprague-Dawley rats treated with losartan or hydrochlorothiazide. (A) Representative photomicrographs (captured at 10X magnification) from level 1 of the PVN (Bregma -1.44 mm to -1.72 mm) of tumor necrosis factor-alpha (TNF-α) in male rats at 16-month-old (MO) with no treatment or with losartan (LOS; 3 mg/kg/day s.c., 21-days); Scale bar (10X) = 200 µm. Representative image for 16-month-old male is also used in Supplemental Figure 9A. **(B)** TNF-α expression presented as percent area in level 1 of the PVN in male rats at 16-months-old with no treatment or with losartan (n=5/group), Data used for 16-month-old male is also used in Supplemental Figure 9B. **(C)** Representative photomicrographs (captured at 10X magnification) from level 3 of the PVN (Bregma -2.04 mm to -2.28 mm) of TNF-α in male rats at 16-months-old with no treatment or with losartan; Scale bar (10X) = 200 µm. Representative image for 16-month-old male is also used in Supplemental Figure 9E. **(D)** TNF-α expression presented as percent area in level 3 of the PVN in male rats at 16-months-old with no treatment or with losartan (n=5/group), Data used for 16-month-old male is also used in Supplemental Figure 9F. Statistical significance was determined using student's T-test, ***$p<0.005$, ****$p<0.0001$.
