# Supplementary material for: Losartan attenuates sex-dependent hypertension, neuroinflammation, and cognitive impairment in the aging male sprague–dawley rat
Source: GeroScience. 2024 Dec 3;47(3):3007–26. doi: 10.1007/s11357-024-01409-4 (PMC12181475; doi:10.1007/s11357-024-01409-4)
Supplement: Supplementary file 2 — Supplementary file2 (DOCX 131 KB) [file 11357_2024_1409_MOESM2_ESM.docx]

**Title:** Losartan attenuates sex-dependent hypertension, neuroinflammation, and cognitive impairment in the aging male Sprague-Dawley rat

**Authors:** Kayla M. Nist, M.S.^1,2^, Hannah Bard, B.A.^2,3^, Brannon McBride, B.S.^2,3^, Angela L. Capriglione, B.S.^1,2^, Jesse D. Moreira, Ph.D., M.S.^2,4^, David H. Farb, Ph.D.^3,5^, Richard D. Wainford, BSc, Ph.D.^2,3,6^

**Affiliations:**

1. Department of Anatomy and Neurobiology, Boston University Chobanian and Avedisian School of Medicine, Boston, MA, USA
2. Whitaker Cardiovascular Institute, Department of Medicine, Boston University Chobanian and Avedisian School of Medicine, Boston, MA, USA
3. Department of Pharmacology & Experimental Therapeutics, Boston University Chobanian and Avedisian School of Medicine, Boston, MA, USA
4. Department of Health Sciences, Sargent College, Boston University, Boston, MA
5. Center for Systems Neuroscience, Boston University, Boston, MA
6. Department of Medicine, Division of Cardiology, Emory University School of Medicine, Atlanta, GA, USA

**Short Title:** Aging, Hypertension, and Cognitive Impairment

**Corresponding Author:** Richard D. Wainford, BSc, Ph.D., F.A.H.A., Professor, Emory University School of Medicine, Division of Cardiology, 1750 Haygood Drive, N220, Atlanta, Georgia 30322. Phone: 404-727-3754 Fax: 404-713-0070; E-mail: [rwainfo@emory.edu](mailto:rwainfo@emory.edu)

**Key words:** aging, hypertension, neuroinflammation, paraventricular nucleus of the hypothalamus, cognitive impairment, angiotensin II type 1 receptor

**List of Abbreviations**

ANG II: angiotensin II

AT1R: angiotensin II type 1 receptor

BBB: blood brain barrier

FITC: fluorescein isothiocyanate-Dextran

GFAP: glial fibrillary acidic protein

HCTZ: hydrochlorothiazide

IL-6: interleukin 6

i.p.: intraperitoneal

i.v.: intravenous

LOS: losartan

MAP: mean arterial pressure

MO: months old

NE: norepinephrine

NOR: novel object recognition test

OLT: object location test

PBS: phosphate buffered saline

PFA: paraformaldehyde

PVN: paraventricular nucleus of the hypothalamus

SD: Sprague-Dawley

TNF-⍺: tumor necrosis factor-⍺

**Abstract**

The prevalence of hypertension increases with age and is the leading modifiable risk factor for cognitive impairment and dementia. At present, the neural mechanisms promoting hypertension across the lifespan are incompletely understood. Using the Sprague-Dawley (SD) rat as a model of normal aging, we hypothesized 1) blood brain barrier (BBB) disruption and neuroinflammation in the paraventricular nucleus (PVN) of the hypothalamus enhances sympathetic tone and contributes to age-dependent hypertension, 2) age-dependent hypertension is associated with cognitive impairment, and 3) lowering blood pressure in aged rats with established hypertension improves cognitive function. We found male, but not female, rats develop age-dependent hypertension with enhanced sympathetic tone, BBB disruption and neuroinflammation in the PVN. Aged hypertensive male rats also showed impairments in recognition and spatial memory. Utilizing pharmacological interventions, blood pressure was lowered in male rats with established hypertension using either losartan (LOS) or hydrochlorothiazide. However, only losartan improved recognition memory. Further, LOS reduced BBB disruption, microglial activation, astrocyte reactivity, and proinflammatory cytokine expression in the PVN which we speculate contributes to a decrease in blood pressure. These data show SD rats develop age-dependent hypertension and cognitive impairment in a sex-dependent manner. However, not all antihypertensive agents improve cognitive function equally as only losartan, an angiotensin II type 1 receptor antagonist (AT1R) improved recognition memory. Thus, AT1R antagonists represent a potential therapeutic approach for treating cognitive decline in the aging population.

**Introduction**

Hypertension is the leading modifiable risk factor for stroke, myocardial infarction, and chronic kidney disease^1^. Further, hypertension is of particular importance in the aging population as it impacts three in four U.S. adults above the age of 65^1^. While hypertension rates are lower in premenopausal women compared to age-matched men, there is a dramatic increase in the incidence of hypertension in postmenopausal women^2^. Hypertension has been identified as a key contributor to cognitive impairment and dementia- a major health issue in the aging population^3^. Moreover, it is suggested that nearly 40% of dementia cases can be prevented or delayed by treating underlying risk factors, such as hypertension^4^. Thus, improving our understanding of the relationship between hypertension and cognitive impairment is critical to potentially treat and prevent cognitive decline, particularly in the context of aging.

Male Sprague-Dawley (SD) rats develop age-dependent hypertension with enhanced sympathetic outflow^5^, suggesting a similar pathology to human hypertension^6^, however, the neural mechanisms underlying age-dependent hypertension remain unknown. There are multiple central nervous system mechanisms that may be modulating sympathetic outflow leading to elevations in blood pressure, particularly those involving the paraventricular nucleus of the hypothalamus (PVN) - a cardio-regulatory brain nucleus central to the sympathetic control of blood pressure^7^. Recent data suggests when the PVN blood brain barrier (BBB), which provides protection to the brain from the peripheral environment, is disrupted in rats with hypertension, circulating factors can gain access to the parenchyma and cause neuroinflammation contributing to excess sympathetic outflow and hypertension^8^. Moreover, PVN neuroinflammation mediated by microglia and proinflammatory cytokines can drive elevations in sympathetic tone and the development of hypertension in young rodents^9–11^. However, this remains unaddressed as a potential mechanism underlying the development and maintenance of age-dependent hypertension.

Multiple human studies have reported a significant relationship between hypertension and cognitive impairment,^12,13^ with the magnitude of hypertension positively correlating with impairments in cognition. Critically, human studies identified midlife hypertension as a risk factor for the development of late life cognitive impairment^14–16^. While studies investigating a relationship between hypertension and cognitive function in rodents have been conducted, they have been limited to young animals. Studies in young angiotensin II infused (ANG II) mice found blood brain barrier disruption drives impairments in cognition^17^. Similarly, studies in young Dahl Salt Sensitive rats suggest neuroinflammation contributes to cognitive impairment which was prevented by angiotensin II type 1 receptor antagonist (AT1R) treatment^18^. However, the potential impact of blood pressure lowering treatments on the cognitive consequences of hypertension in aged rodents has yet to be addressed.

We hypothesized 1) blood brain barrier disruption and neuroinflammation in PVN enhances sympathetic tone and contributes to age-dependent hypertension, 2) age-dependent hypertension is associated with cognitive impairment, and 3) lowering blood pressure in aged rats with established hypertension improves cognitive function. To test this, we used male and female SD rats at 3-, 8-, and 16-months-old to model normal aging. We assessed blood pressure, sympathetic tone, BBB permeability, neuroinflammation, and recognition and spatial memory. We then lowered blood pressure in aged male rats with established hypertension and cognitive impairment using either losartan (LOS), an AT1R antagonist, or hydrochlorothiazide (HCTZ), a sodium chloride co-transporter antagonist, and reassessed these parameters. Collectively, these studies provide new mechanistic insight into the sex-dependent development of age-dependent hypertension in SD rats and suggest a potential therapeutic approach for the treatment of cognitive impairment in the aging population.

**METHODS**

Animals

Male and female SD rats aged 3-, 8-, and 16-months-old (MO) (equivalent to ~20 (young adult), 45 (midlife), and 60 (aged) human years of age, respectively) were acquired from Envigo (Indianapolis, IN, USA) as previously published by our laboratory^5^. Rats were pair housed until surgical intervention or body weight requirements were exceeded in a 12:12-hour light-dark cycle, temperature (20-26˚C) and humidity-controlled (30-70%) environment. Rats were allotted tap water and standard irradiated rodent diet (Envigo Teklab, WI, Teklab Global Diet #2918, 18% protein, 5% crude fat, 5% fiber, total NaCl content 0.6% [174 mEq Na+/kg]) ad libitum. All animal protocols used were approved by the Institutional Animal Care and Use Committee at Boston University Chobanian and Avedisian School of Medicine in agreement with guidelines set by the university and the National Institutes of Health *Guide for the Care and Use of Laboratory Animals*. Every possible step was taken to minimize pain and suffering. Euthanasia was performed as per the above guidelines, in accordance with the approved IACUC protocol number PROTO201800201 at Boston University.

## Surgical Procedures

### *Acute Femoral Artery and Femoral Vein Cannulation*

Male and female SD rats (N=5-6/group) were anesthetized using sodium methohexital (20 mg/kg intraperitoneally (i.p.), with 10 mg/kg administered intravenously (i.v.) as needed). Cannulation of the femoral artery and vein was performed as previously described^5,19^. An incision was made in the left femoral triangle and the femoral artery and vein were dissected from the adjacent tissue. Using a cannula made from PE-50 tubing, the cannula was inserted into the femoral vein to allow for administration of i.v. anesthesia, isotonic saline during recovery periods, as well as hexamethonium administration. A PE-50 cannula was placed in the left femoral artery to record heart rate and mean arterial pressure (MAP). Cannulas were tied in place using sutures and the incision was closed. Rats were positioned in a Plexiglas rat holder and the cannula for the femoral artery was attached to an external pressure transducer, while the cannula for the femoral vein was attached to an infusion pump. A two-hour recovery period was allowed, during which an i.v. infusion of isotonic saline (20 μL/min) was performed and rats returned to full consciousness, with stable cardiovascular and renal function. After the two-hour recovery period, baseline MAP was continuously recorded over a one-hour period in conscious rats through the femoral artery cannula using the computer-driven data acquisition software, MP150 and AcqKnowledge 3.8.2 (BIOPAC, CA).

### *Intravascular Infusion of Dextrans*

Male and female SD rats (N=6/group) were anesthetized (30 mg/kg ketamine i.p., 3 mg/kg xylazine i.p.)^20^ and an incision was made into the left carotid triangle. Muscles were mobilized to expose the carotid sheath. The carotid artery was dissected from surrounding tissues and isolated. A PE-50 cannula was placed into the exposed left common carotid artery and secured^8^. Rats were infused with fluorescein isothiocyanate-Dextran (FITC) at 10 kDa (10 mg/mL, FD10S, Sigma-Aldrich) and rhodamine B isothiocyanate-Dextran at 70 kDa (10 mg/mL, R9379, Sigma-Aldrich) over 60 seconds (~10 μL/1 s). Fluorescent dextrans were allowed to circulate for 20 minutes before animals were sacrificed via decapitation. An additional group of 3MO male rats (N=6) were infused with hypertonic mannitol (1.4 mol/L, 2 mL per 200-250 g) 5 minutes before the dextran infusion as a positive control (**Supplemental Figure 1**)^8^. After sacrifice, brain tissue was extracted and post-fixed in 4% paraformaldehyde (PFA) for 48-72 hours, then immersed in 30% sucrose solution until sectioning.

*Osmotic Mini Pump Insertion*

Male rats aged 16MO (N=5-6/group) were treated with either losartan potassium (LOS; 3 mg/kg/day in sterile saline for 21 days; L0232, TCI)^19^ or hydrochlorothiazide (HCTZ; 4 mg/kg/day in 50:50 DMSO:Saline for 14 days; H4759, Sigma)^5^ using 2ML4 pumps (Alzet). In brief, rats were anesthetized using a ketamine/xylazine cocktail (30 mg/kg i.p. ketamine; 3 mg/kg i.p. xylazine), an incision was made in the subscapular region and a subcutaneous pocket was made. An osmotic mini pump was implanted and the incision was closed with suture.

*Transcardiac Perfusion*

Male and female SD rats were anesthetized with a ketamine/xylazine cocktail (30 mg/kg ketamine i.p., 3 mg/kg xylazine i.p). Once anesthetic depth was reached and there was an absence of toe pinch response, the thoracic cavity was opened and a needle was placed in the heart attached to a perfusion pump. Rats were perfused with ice-cold 1X phosphate-buffered saline before perfusion with 4% PFA. Once fixation was achieved, perfusion was stopped and brains was harvested. Brains were post-fixed in PFA for 48-72 hours before being moved to 30% sucrose for sectioning.

## In Vivo Studies

## *Assessment of Vascular Sympathetic Tone*

In animals that underwent acute femoral artery and vein cannulation, peak depressor response to ganglionic blockade (hexamethonium, 30 mg/kg, i.v., H2138, Sigma-Aldrich) was used to estimate sympathetic tone to the vasculature as previously described^5,19^. Baseline blood pressure was defined as the average blood pressure that occurred during the ten minutes before the bolus of hexamethonium was administered. The lowest blood pressure measurement that occurred within five minutes of the administration of hexamethonium was used to calculate the difference compared with baseline blood pressure.

*Novel Object Recognition Task*

For novel object recognition (NOR) testing^21^, rats were placed in a 60 by 60 cm open topped behavioral testing arena with a video camera fixed above to record testing for manual analysis. Two different objects were placed in separate quadrants of the arena placed 6 inches from adjacent corners. For familiarization, rats were placed in the center of the arena and allowed to explore the environment and objects for 10 minutes. Following familiarization, rats were returned to their home cages for 2 hours. Following the 2-hour latency, rats were returned to the testing arena, this time with a novel object replacing one of the familiar objects. Rats were allowed to explore the environment and objects for 10 minutes to complete the test portion of the assessment. After testing rats were returned to their home cages. The testing arena and objects were thoroughly cleaned between each test with 10% ethanol.

*Object Location Task*

Object location (OLT) testing^22^ was completed in the same open topped behavioral testing arena as described in the *Novel Object Recognition Task*. Familiarization was carried out in the same manner. Following the 2-hour latency, rats were returned to the testing arena, this time with one of the familiar objects placed in the opposite quadrant from its original location. Following 10 minutes of exploration, testing was complete and rats were returned to their home cages and the arena was cleaned with 10% ethanol.

In 16MO male rats treated with losartan or hydrochlorothiazide, novel object recognition task and object location task were completed at baseline prior to the initiation of treatment and at the end of the treatment period in order to assess the impact of treatment on cognitive performance in a within subjects design.

The time a rat spent investigating each object separately was measured in order to calculate time investigating the novel object or location and the total time spent investigating both objects. These measures were used to calculate the familiarization index, discrimination index, and the percent of time exploring the novel object/location.

Familiarization score was calculated as the absolute value of the time spent at one object subtracted by the time spent at the other object divided by the total time spent investigating the objects. This score was used to assess any preference a rat may have for one object over the other.

$$familiarization=\frac{\left( time spent at right object \right)-\left( time spent at left object \right)}{time spent at both}$$

Discrimination index is a measure of sensitivity and is assessed as the time spent investigating the novel object/location minus the time spent investigating the familiar object/location divided by the total time spent investigating both objects/locations. Percent of time exploring the novel object/location was assessed time spent investigating the novel object/location divided by the total amount of time spent investigating.

$$discrimination index=\frac{\left( time spent at novel object or location \right)-\left( time spent at familiar object or location \right)}{total time spent at both}$$

##

## Molecular Techniques

## *Assessment of Plasma Norepinephrine Content and Plasma Estradiol Content*

Plasma taken from male and female SD rats following conscious decapitation was used to measure Plasma norepinephrine (NE) via ELISA (IB89552, IBL America) according to manufacturer’s directions as previously performed by our laboratory^5,11^. To measure plasma estradiol, plasma taken from female rats following conscious decapitation was used to measure 17β estradiol via ELISA (IB79329, IBL America) according to manufacturer’s directions as previously performed by our laboratory^5^.

*Assessment of Plasma Progesterone Content*

Plasma progesterone was measured at the University of Mississippi Medical Center Mass Spectrometry Core using LC-MS/MS analysis. All reagents used were high throughput liquid chromatography grade and purchased from Millipore Sigma including water, methanol, acetonitrile, progesterone materials and isotope-labeled internal standard for progesterone (Progesterone-D9). Cold-induced phase separation was used to extract progesterone from plasma. Briefly, 100µL of plasma and 10µl of Internal standard solution (100ng/ml isotype-labeled internal standards) were added to 200µl cold acetonitrile and vortexed before an additional 100µl HPLC water was added. The samples were centrifuged (10,000 g, 2min, 4℃) and stored at -20℃ for 30 minutes to induce phase separation. After the first incubation, the upper layer (100µl) was transferred to a separate tube containing 100µl NaHCO3 and then incubated 10 minutes at 65℃. The samples were then placed back into -20℃ for 45 minutes for phase separation. After 45 minutes, the upper phase (50µl) was added to 50µl HPLC water and used for LC-MS/MS analysis. Equipment for detection consisted of an Eksigent M5 microLC with autosampler coupled to a Sciex Qtrap 7500 mass spectrometer. For LC separation, Formic acid in water (0.1% v/v) and formic acid in 75% acetonitrile (0.1% v/v) were used as mobile phases A and B, respectively. The gradient was set as 0-1 min 20-40% B, 1-4.5 min 40-95% B, 4.5-5 min 95% B, 5-5.5min 95-2%B, and 5.5-8 min 2% B. A Phenomenex Luna 5µm C18 100Å (150 x 0.3mm) LC column was used. The flow rate and column temperature are set at 10µl/min and 30℃ with an injection volume of 5µL. The electrospray ionization parameters are as follows: curtain gas (40.0 psi), CAD gas (medium), ionspray voltage (4000 V), source temperature (350°C), ion source gas 1 (30.0 psi), and ion source gas 2 (70.0 psi), Q0 dissociation (40V). The multiple reaction monitoring transitions used for quantification were as follows: Progesterone, 315.2→97.0, Progesterone-D9, 324.2→99.9. All peaks were automatically integrated via SCIEX OS AutoPeak integration algorithm and analyzed in Sciex OS v.3.3.

### *Vascular Integrity of the BBB*

Following intravascular infusions of dextrans, post-fixed tissue was sectioned at 40µm and cryoprotected. PVN tissue was selected between bregma -1.6 mm to -2.16 mm, washed in phosphate-buffered saline (PBS) and mounted on gelatin-subbed slides. Coverslips were secured using Prolong Diamond Antifade Mountant with DAPI (P36971, Invitrogen). Analysis is described below.

###

### *Immunohistochemistry*

Immunohistochemistry was performed as previously described by our laboratory^5,11^. Brain tissue from perfused rats was sectioned at 40 μm on a cryostat and cryoprotected. Multiple sections sampling all levels of the PVN were selected using Paxinos & Watson’s *The Rat Brain* atlas, between Bregma -1.6 mm and Bregma -2.16. For immunohistochemistry, sections were washed in 0.1M PBS 3 times for 10 minutes each and then incubated in 0.3% hydrogen peroxide for 30 minutes. Sections were then blocked and permeabilized in PBS-diluent (0.01M PBS with 3% normal horse serum and 0.25% Triton X-100) for 2 hours before incubation in primary antibodies mouse OX-42/CD11b/c for microglia^11^ or mouse glial fibrillary acidic protein (GFAP) for astrocytes^23^ for 2 hours at room temperature and then incubated overnight at 4℃. All antibody information can be found in **Supplemental Table 1.** Following incubation in primary antibodies, sections were washed in PBS then incubated in biotinylated goat anti-mouse IgG (1:100, BA-9200, Vector Laboratories). Sections were washed again in PBS and incubated in avidin and biotin. Sections were developed in 0.04% 3,3’-diaminobenzidene and 0.04% nickel ammonium sulfate in 0.1M PBS, mounted on gelatin-subbed slides and dehydrated overnight. Coverslips were secured using Permount mounting medium. Negative controls were performed. One control was performed with the omission of the primary antibody and the inclusion of the secondary antibody, while another control was performed with the inclusion of the primary antibody and the omission of the secondary antibody (**Supplemental Figures 2-4**).

*Immunofluorescence*

For immunofluorescence, PVN sections were selected as described above. Tissue was washed in PBS 3 times for 10 minutes each and blocked in PBS-diluent for 2 hours (0.01M PBS with 3% normal goat serum and 0.25% Triton X-100). Sections were then incubated in primary antibodies for mouse interleukin 6 (IL-6)^24^ or mouse tumor necrosis factor-⍺ (TNF-⍺)^25^ at room temperature for 2 hours and then incubated overnight at 4℃. All antibody information can be found in **Supplemental Table 1**. Tissue was brought to room temperature and washed 3 times for 10 minutes each and incubated in appropriate secondary antibodies for goat anti-mouse AlexaFluor 594 (1:500, A-11005, Invitrogen) for 2 hours. At the end of the incubation, sections were washed 3 times for 10 minutes each and mounted on gelatin-subbed slides and allowed to dry. Coverslips were secured using Prolong Diamond Antifade Mountant with DAPI counterstain. Negative controls were performed. One control was performed with the omission of the primary antibody and the inclusion of the secondary antibody, while another control was performed with the inclusion of the primary antibody and the omission of the secondary antibody (**Supplemental Figure 2-4**).

###

### *Microscopy and Image analysis*

Sections were imaged on a Keyence BZ-9000 Fluorescence Microscope with brightfield capabilities. Images were captured at 10X, 20X, and 40X magnifications. Dichroic filters for Texas Red at 585nm, GFP at 495nm, and DAPI at 400nm were used. A representative schematic of PVN neuroanatomy at 10X is included in **Supplemental Figure 5**.

For assessment of BBB integrity, 20X images of FITC and Rhodamine were obtained and opened in FIJI/ImageJ. The images were converted to 16-bit and the colocalization threshold tool was used to isolate matching signal in both images. Images were converted to binary and the FITC image was subtracted from the colocalization image in order to expose FITC only signal. The percent area of FITC extravasation was calculated by using the threshold tool to select all areas positive for signal within the PVN.

For microglia analysis, images of the left and right PVN were taken at 40X magnification in order to assess morphology and perform Sholl analysis as previously described by our laboratory^11^. A 200 µm by 200 µm box was randomly selected and used for analysis. Microglia were deemed active if a majority of the process lengths were less than that of the diameter of the soma. The number of active microglia and the total number of active microglia were measured. For Sholl analysis, 8 individual microglia from each 40X image were selected at random and cropped for isolation using FIJI/ImageJ. The area of the individual microglia was selected using the threshold tool and the center of the specific randomly selected microglia was identified. Concentric rings 1µm apart were placed on the image and the number of branching intersections for each randomly selected microglia were calculated (**Supplemental Figure 6**).

For astrocytes (GFAP), IL-6, and TNF-⍺ analysis, 20X images of the left and right PVN were used to assess protein expression as total percent area. Images were converted to 8-bit and positive areas were selected using the threshold tool and particle analysis was performed.

## *Statistical Analysis*

All data are presented as mean ± standard deviation with p<0.05 as significance. A Pearson’s r correlation was performed to assess correlation with age and blood pressure as variables. A one-way ANOVA was used to assess group differences for all analyses with age as a set variable. Post-hoc analysis was performed using a Tukey test. For behavioral comparison between 16 MO male rats and LOS or HCTZ treatment, a repeated measures t-test was used. Prism 9 (GraphPad Software, CA) was used to carry out statistical analysis.

**Results**

**Male, but not female, SD rats develop age-dependent hypertension and sympathoexcitation.** There was a progressive increase in MAP in aging male SD rats (**Figure 1A**). Sympathetic tone to the vasculature increased to a similar magnitude in both 8 and 16MO male rats (**Figure 1B**) and global sympathetic tone increased with age in male SD rats (**Figure 1C**). Female rats showed no change in blood pressure or sympathetic tone with age (**Figure 1D-F**). Elevations in blood pressure and global sympathetic tone positively correlate with increased age in male (Pearson’s r correlation; MAP: r=0.9502, p<0.0001; Plasma NE: r=0.09282, p<0.0001), but not female rats (Pearson’s r correlation; MAP: r=0.1739, p=0.4902; Plasma NE: r=-0.0402, p=0.8741). In female rats, plasma 17β-estradiol increased at 8MO, however there was no change between 3 and 16MO (**Supplemental Figure 7**). Plasma progesterone did not change with age (**Supplemental Figure 7**).

**Male, but not female, SD rats have a disrupted BBB in the PVN.** BBB disruption, as evidenced by significant FITC-dextrans (10 kDa) extravasation into the parenchyma of the PVN was present to the same extent in male rats at 8 and 16MO (**Figure 2A&B, Supplemental Figure 8**). In contrast, female rats did not exhibit FITC extravasation in the PVN with age (**Figure 2C&D, Supplemental Figure 8**). To validate dextrans extravasation as an assessment of BBB permeability, a separate group of 3MO male rats was infused with hyperosmolar mannitol, prior to the co-infusion of dextrans as a positive control. While untreated 3-MO male rats maintained an intact PVN BBB, 3MO male rats treated with mannitol had increased FITC extravasation indicative of increased BBB permeability (**Supplemental Figure 1**).

**Neuroinflammation increases in the PVN of male, but not female, SD rats.** Morphological assessment of microglia was performed to quantify the number of active and the total number of microglia in the PVN. The number of active (hypertrophic or ameboid) microglia in the PVN increased with age in male rats (**Figure 2E&F, Supplemental Figure 9**) while the total number of microglia had no change with age (**Figure 2E&G, Supplemental Figure 9**). Sholl analysis for branching complexity revealed that 16MO rats had a significant decrease in branching complexity compared to 3-MO and 8MO rats, indicative of increased microglial activation (**Figure 2L&M**). No difference detected between 3MO and 8MO rats (**Figure 2K**). Female rats had no change in the number of active or total number of microglia in the PVN (**Figure 2H-J, Supplemental Figure 9**). Astrocyte reactivity was assessed by tissue density of GFAP expression. Consistent with the increase in active microglia, GFAP expression increased in male rats (**Figure 2N&O, Supplemental Figure 10**), but not female rats with age (**Figure 2P&Q, Supplemental Figure 10**). Cytokine expression was analyzed as percent area of the PVN positive for IL-6 or TNF-⍺ staining. Male rats had a significant increase in both IL-6 and TNF-⍺ expression with age (**Figure 3A,B,E,F, Supplemental Figures 11&12**) while female rats showed no change in expression of either cytokine (**Figure 3C,D,G,H, Supplemental Figures 11&12**).

**Recognition and spatial memory are impaired in hypertensive male, but not normotensive female, SD rats with age.** On familiarization, male and female rats showed no preference in either task (**Table 1**). Aged male rats had decreased discrimination index on both tasks and spent less time at the novel object or location, indicative of impairments in recognition and spatial memory with age (**Figure 4C,E,G,I**). Female rats showed no change in discrimination index or time spent at novel object or location with age, indicative of no age-related impairments in recognition or spatial memory (**Figure 4D,F,H,J**).

**Losartan and hydrochlorothiazide lower blood pressure in aging male SD rats.** To achieve blood pressure reduction, we administered 21-days of losartan or 14-days of hydrochlorothiazide. MAP was lowered to similar levels in both treatment groups (**Figure 5A**). It should be noted that MAP data from 16-MO untreated male rats are the same values used in Figure 1A.

**Losartan improves recognition memory in aging male SD rats.** Rats were tested on NOR task and OLT both prior to initiation of treatment and at the end of the treatment as a within subjects’ study design. On familiarization for both pre- and post-treatment, rats showed no preference toward either object on either the NOR or the OLT (**Table 2**). In rats treated with losartan, recognition memory significantly improved compared to pretreatment (**Figure 5B&C**). However, losartan treatment did not improve spatial memory compared to pretreatment (**Figure 5F&G**). Treatment with hydrochlorothiazide did not improve recognition memory or spatial memory compared to pretreatment (**Figure 5D,E,H,I**).

**Losartan attenuates BBB disruption and neuroinflammation in aging male SD rats.** It should be noted that data and representative images for 16MO male rats are the same as those used in Figure 2 and 3. LOS treatment decreased BBB permeability (**Figure 6A&B, Supplemental Figure 13**), attenuated the number of active microglia in the PVN, without changing the total number of microglia (**Figure 6C-E, Supplemental Figure 14**), increased branching complexity (**Figure 6F**), and decreased GFAP expression (**Figure 6H-I, Supplemental Figure 15**). HCTZ showed no changes in any of these parameters compared to untreated 16-month-old male rats (**Figure 6A-E,G,H,I, Supplemental Figures 13-15**). Because no changes were detected in HCTZ treated rats, cytokine expression was only measured in LOS treated rats. Here, LOS was found to attenuate both IL-6 and TNF-⍺ expression compared to untreated rats (**Figure 6J-M, Supplemental Figures 16&17**).

**Discussion**

These studies were designed to investigate the hypothesis that 1) BBB disruption and neuroinflammation in the PVN contributes to age-dependent hypertension in SD rats, 2) age-dependent hypertension in SD rats is associated with cognitive impairment, and 3) the use of antihypertensive therapeutics to lower blood pressure in aged SD rats with established hypertension improves cognitive function. These studies reveal that normal aging evokes hypertension and enhanced sympathetic outflow that is associated with PVN BBB disruption and neuroinflammation in male but not female SD rats. Aged hypertensive male rats have impairments in recognition and spatial memory. The AT1R antagonist LOS proved to have pleiotropic benefits, improving recognition memory while attenuating PVN neuroinflammation and BBB disruption and lowering blood pressure in aged hypertensive male rats. This same effect was not seen following HCTZ treatment, which only lowered blood pressure. These findings add to the growing body of literature implicating hypertension in the development of cognitive impairment and support AT1R blockers as a treatment for cognitive impairment in age-dependent hypertension.

In this study we observed male but not female SD rats develop age-dependent hypertension with enhanced sympathetic tone^5^ modeling human hypertension in aging populations^6^. While our prior findings reported an increase in blood pressure with age in male rats, in the groups of rats utilized in these studies we observed a greater spread in blood pressure values. Additionally, while our prior study identified a difference in global sympathetic tone between 3 and 16MO male rats, we found a progressive increase in global sympathetic tone between all age groups. These findings suggest, as observed in human populations, there is variability in the development of hypertension in the aging SD rat. Taken together, along with data indicating an increase in sympathetic outflow to the vasculature with age, these findings suggest hypertension is driven in part by alterations in sympathetic nervous system activity in aged male SD rats.

In aging male rats, we found an increase in FITC extravasation in the PVN, indicative of BBB disruption. This occurred along with neuroinflammation indicated by an increase in active microglia, reactive astrocytes, and increased expression of proinflammatory cytokines IL-6 and TNF-⍺. Prior studies report an increase in BBB disruption in Spontaneously Hypertensive Rats^8,26^, with one study suggesting this allows circulating factors such as ANG II to gain access to the PVN from the circulation and induce neuroinflammation mediated by microglia^8^. Moreover, prior studies in hypertensive animal models (i.e., G⍺i_2_ salt sensitive rats, ANG II infusion rats) found microglial activation and proinflammatory cytokine production in the PVN was associated with enhanced sympathetic tone and elevated blood pressure^10,11^. Further, minocycline, an anti-inflammatory antibiotic, reduced microglial activation and neuroinflammation in the PVN, modulated sympathetic tone and decreased blood pressure in multiple rodent models of hypertension that exhibit neuroinflammation^9–11^. Interestingly, direct microinjections of proinflammatory cytokines TNF-⍺ or IL-1β into the PVN increased blood pressure, renal sympathetic nerve activity, and enhanced the cardiac sympathetic afferent reflex^27^. Further, pretreatment with TNF-⍺ and IL-1β in the PVN sensitized rats to developing sympathoexcitation and hypertension in response to a subpressor dose of ANG II^27^. This suggests a relationship between proinflammatory cytokines and elevated blood pressure and that proinflammatory cytokines in the PVN can predispose a rat to developing hypertension. While we did find an increase in both IL-6 and TNF-⍺, we did not determine if they are gaining access to the PVN due to the disrupted BBB or if they are being produced within the PVN by microglia or other inflammatory cells. While studies of obesity identified astrocyte reactivity in multiple brain regions^28^, including the PVN, our study appears to be the first to find an increase in GFAP, indicative of astrocyte reactivity, in the PVN of hypertensive rats^11^. We speculate that astrocyte reactivity may contribute to enhanced sympathetic outflow seen in our model as a prior study found an inhibition in glutamate transport by astrocytes in the PVN leads to activation of glutamate receptors on presympathetic neurons increasing activity and driving sympathoexcitation^29^. Collectively our findings suggest PVN BBB disruption and neuroinflammation is contributing significantly to increased sympathetic outflow and blood pressure in aging male rats.

Our data reveal impairments in performance on the NOR and OLT, indicative of impairments in recognition and spatial memory in aged hypertensive male rats. This suggests an association between increased blood pressure and cognitive impairment with age, which is also seen in human studies such as SEARCH-Health and MEMENTO^12,13^. Our studies continue to support an association between hypertension and cognitive impairment which is also seen in young hypertensive rodents (i.e., ANG II infused mice, Dahl Salt Sensitive rats)^17,18^. Those studies further revealed hypertension-related cognitive impairment is associated with neurovascular dysfunction, including BBB disruption, microbleeds, and neuroinflammation in the hippocampus and cortex.^17,18^ Thus, we speculate these may be mechanisms mediating impairments in cognitive function in aging SD rats that can be further investigated in future studies.

In opposition to human hypertension, our female rats showed no age-related increase in blood pressure and sympathetic tone, which is consistent with our previous findings^5^, and no evidence of BBB disruption, neuroinflammation, or cognitive impairment. While humans experience a significant loss of female sex steroids during menopause, female SD rats enter persistent estrous following cycle cessation between 8-12 MO^30^. This is supported by our findings that female rats maintained plasma estradiol and progesterone concentrations with age. The difference in plasma estrogen at 8MO may be the result of changes in cycles. As we did not complete vaginal cytology, we cannot confirm this. Our studies emphasize persistent sex differences in hypertension that are consistent with our prior findings and other rodent models of hypertension^5,31,32^. Estrogen augments tight junction function limiting BBB disruption^33^ and activation of estrogen receptors on microglia and astrocytes is known to prevent neuroinflammation^34,35^. Further, loss of estrogen impairs cognition^36^. Accordingly, the maintenance of estrogen throughout the lifespan may mediate neuroprotection in our aging female rats and supports the future exploration of female sex steroid contributions in age-dependent hypertension.

We next tested the hypothesis that lowering blood pressure can improve cognitive function in rats with established hypertension. Both LOS, an AT1R antagonist, and HCTZ, a thiazide diuretic, lowered blood pressure to the same magnitude. Our results revealed losartan improved recognition memory to the performance observed in 3 and 8MO male rats. LOS, however, did not improve spatial memory and hydrochlorothiazide had no impact on cognitive function. This lack of improvement in spatial memory was surprising given that other studies in rodents with hypertension (young Dahl Salt Sensitive rats)^18^ and with Alzheimer’s Disease^37^ found spatial memory improves following AT1R blockade at doses that do not alter blood pressure. The use of different behavioral tasks to assess spatial memory in those studies compared to the presented study may be responsible for the discrepancies between findings. Our findings suggest the cognitive benefits of an AT1R blockade may be independent of blood pressure lowering mechanisms. In human studies, including SPRINT-MIND, HYVET, and PROGRESS, blood pressure reduction, regardless of drug class, significantly decreased risk of developing cognitive impairment and dementia^38–40^. This is in contrast to other studies, like CALIBREX, which showed favorable cognitive benefits in the use of AT1R antagonists over thiazide diuretics and ACE inhibitors^41,42^. In combination with our findings and others in rodent models and human subjects, we speculate drug class, specifically AT1R blockers, may be important for the treatment of hypertension-related cognitive dysfunction.

Our studies also revealed LOS attenuated BBB disruption and diminished neuroinflammation in the PVN of aged male rats to levels seen in young normotensive male rats. Prior studies indicate AT1R blockade in the Spontaneously Hypertensive Rat improved PVN BBB dysfunction and neuroinflammation leading to a reduction in sympathetic outflow and lower blood pressure^8,24^, suggesting AT1R activation contributes to BBB disruption and neuroinflammation. This is further supported by improvements in blood brain barrier disruption and neuroinflammation by AT1R blockade in regions outside of the PVN, like the hippocampus and cortex in hypertensive models^18,43^. A potential mechanism that we speculate underlies these improvements in BBB function and the anti-inflammatory impact of LOS may be a result of the blockade of AT1R on endothelial cells, astrocytes, microglia, and neurons^29,43^. Supporting this is the reported anti-inflammatory effect of AT1R antagonists in Alzheimer’s Disease and traumatic brain injury^37,44^. Although we did not address this in this study, we speculate there may be neuroinflammation in regions associated with learning and memory, such as the hippocampus, which could modulate cognition and be improved following LOS treatment. Taken together, we propose the aging SD rat as a model of hypertension-associated cognitive impairment and these findings suggest LOS provides unique neurocognitive and neuroimmune benefits compared to HCTZ, particularly in established hypertension.

Our hypothesis was that lowering blood pressure would improve cognitive function in aged male SD rats with established hypertension. Drug doses and durations of administration were selected as they have previously been shown to effectively lower blood pressure^5,19^. Both compounds are first-line antihypertensive therapeutics. LOS is one of many AT1R blockers, a class which includes Telmisartan and Olmesartan, used to treat blood pressure. Multiple studies have revealed beneficial off-target effects of AT1R blockers including anti-inflammatory effects^8,24^. Further, telmisartan has been shown to cross the BBB while LOS may not cross the BBB as readily^45^. However, we elected to use LOS in these studies as it was previously found to effectively improve BBB permeability and neuroinflammation in the PVN in Spontaneously Hypertensive rats^8^. Given that aging male SD rats have increased BBB permeability, we speculate that losartan may gain access to the brain parenchyma as a result, contributing to a reduction in neuroinflammation and improvements in cognitive function seen in these studies.

*Study Limitations*

First, blood pressure was measured using acute instrumentation rather than radiotelemetry. While radiotelemetry is recognized as the gold standard of blood pressure measurements in rodents, our previous findings in aging SD rats identified the same trend and magnitude of blood pressure increases with age when measured using telemetry vs. acute instrumentation^5^. We acknowledge there are other regions in the brain that may also modulate sympathetic outflow and they may contribute to age-related changes in the regulation of blood pressure. However, we limited our studies to investigating the PVN’s contributions to age-dependent hypertension due to its central role in sympathetic outflow and blood pressure regulation. The current studies only used two cognitive assessments and therefore only assessed recognition and spatial memory. These tasks were utilized as they involved minimal training for the animal and do not evoke profound changes in blood pressure due to stress or movement in order to complete the task. In the future, more behavioral assessments including the Barnes Maze will be utilized to further assess different cognitive domains. Further studies are necessary to better understand the underlying contributions of the hypertension-related changes in the hippocampus and its impact on cognition. It should be noted that anesthesia can increase the permeability of the BBB, which may explain a small amount of FITC extravasation in normotensive male and female rats. Ketamine/xylazine anesthesia was used in these studies as it has previously been reported to limit BBB permeability compared to other anesthetics.

**Perspectives**

This study is the first to establish that BBB disruption and neuroinflammation in the PVN contributes to age-dependent hypertension in a sex-dependent manner and that age-dependent hypertension is associated with cognitive impairment in the SD rat. However, while lifelong elevations in blood pressure contribute to cognitive impairment, attenuating hypertension-associated cognitive impairment is more nuanced that just lowering blood pressure as LOS provides pleiotropic benefits independent of its blood pressure lowering abilities. Our findings suggest not all antihypertensive medications are equal in improving cognitive performance or attenuating neuroinflammation, even when blood pressure is effectively lowered (**Figure 7**). While our results support the use of AT1R antagonists to improve cognitive function in hypertensive individuals, we speculate based on our findings and others that the use of an AT1R blockade at a dose that does not impact blood pressure in normotensive individuals or individuals with elevated blood pressure may enhance cognitive outcomes, particularly in the aging population.

**Author Contributions:** KMN designed studies, conducted experiments, analyzed data, and wrote the manuscript. HB, BM and AC conducted experiments and analyzed data. JDM conducted experiments. DHF contributed to manuscript writing and editing. RDW designed studies, conducted experiments, and wrote the manuscript.

**Sources of Funding:** This work was supported by the National Institutes of Health (NIH) grants R01 HL139867, R01 HL141406, R01 AG062515, and R01 AG075963 and Hevolution Foundation grant HF-GRO-23–1199246-43 to R.D.W., and R01 AG075963 to DHF.

**Disclosures:** The authors have declared that no conflict of interest exists.

**References**

1. Whelton PK, Carey RM, Aronow WS, Casey DE, Collins KJ, Dennison Himmelfarb C, DePalma SM, Gidding S, Jamerson KA, Jones DW, MacLaughlin EJ, Muntner P, Ovbiagele B, Smith SC, Spencer CC, et al. 2017 ACC/AHA/AAPA/ABC/ACPM/AGS/APhA/ASH/ASPC/NMA/PCNA Guideline for the Prevention, Detection, Evaluation, and Management of High Blood Pressure in Adults: A Report of the American College of Cardiology/American Heart Association Task Force on Clinical Practice Guidelines. *Hypertension*. 2018;71(6):e13–e115.

2. Wassertheil-Smoller S, Anderson G, Psaty BM, Black HR, Manson J, Wong N, Francis J, Grimm R, Kotchen T, Langer R, Lasser N. Hypertension and Its Treatment in Postmenopausal Women. *Hypertension*. 2000;36(5):780–789.

3. Gorelick PB, Scuteri A, Black SE, DeCarli C, Greenberg SM, Iadecola C, Launer LJ, Laurent S, Lopez OL, Nyenhuis D, Petersen RC, Schneider JA, Tzourio C, Arnett DK, Bennett DA, et al. Vascular Contributions to Cognitive Impairment and Dementia. *Stroke*. 2011;42(9):2672–2713.

4. Livingston G, Huntley J, Sommerlad A, Ames D, Ballard C, Banerjee S, Brayne C, Burns A, Cohen-Mansfield J, Cooper C, Costafreda SG, Dias A, Fox N, Gitlin LN, Howard R, et al. Dementia prevention, intervention, and care: 2020 report of the Lancet Commission. *The Lancet*. 2020;396(10248):413–446.

5. Frame AA, Nist KM, Kim K, Puleo F, Moreira JD, Swaldi H, McKenna J, Wainford RD. Integrated renal and sympathetic mechanisms underlying the development of sex- and age-dependent hypertension and the salt sensitivity of blood pressure. *GeroScience*. 2024. 10.1007/s11357-024-01266-1.

6. Esler M, Hastings J, Lambert G, Kaye D, Jennings G, Seals DR. The influence of aging on the human sympathetic nervous system and brain norepinephrine turnover. *American Journal of Physiology. Regulatory, Integrative and Comparative Physiology*. 2002;282(3):R909-916.

7. Dampney RA, Michelini LC, Li D-P, Pan H-L. Regulation of sympathetic vasomotor activity by the hypothalamic paraventricular nucleus in normotensive and hypertensive states. *American Journal of Physiology - Heart and Circulatory Physiology*. 2018;315(5):H1200–H1214.

8. Biancardi VC, Son SJ, Ahmadi S, Filosa JA, Stern JE. Circulating Angiotensin II Gains Access to the Hypothalamus and Brain Stem During Hypertension via Breakdown of the Blood–Brain Barrier. *Hypertension*. 2014;63(3):572–579.

9. Santisteban MM, Ahmari N, Carvajal JM, Zingler MB, Qi Y, Kim S, Joseph J, Garcia-Pereira F, Johnson RD, Shenoy V, Raizada MK, Zubcevic J. Involvement of Bone Marrow Cells and Neuroinflammation in Hypertension. *Circulation Research*. 2015;117(2):178–191.

10. Shi P, Diez-Freire C, Jun JY, Qi Y, Katovich MJ, Li Q, Sriramula S, Francis J, Sumners C, Raizada MK. Brain Microglial Cytokines in Neurogenic Hypertension. *Hypertension*. 2010;56(2):297–303.

11. Moreira JD, Chaudhary P, Frame AA, Puleo F, Nist KM, Abkin EA, Moore TL, George JC, Wainford RD. Inhibition of microglial activation in rats attenuates paraventricular nucleus inflammation in Gαi2 protein-dependent, salt-sensitive hypertension. *Experimental Physiology*. 2019;104(12):1892–1910.

12. Knecht S, Wersching H, Lohmann H, Bruchmann M, Duning T, Dziewas R, Berger K, Ringelstein EB. High-Normal Blood Pressure Is Associated With Poor Cognitive Performance. *Hypertension*. 2008;51(3):663–668.

13. Lespinasse J, Chêne G, Mangin J-F, Dubois B, Blanc F, Paquet C, Hanon O, Planche V, Gabelle A, Ceccaldi M, Annweiler C, Krolak-Salmon P, Godefroy O, Wallon D, Sauvée M, et al. Associations among hypertension, dementia biomarkers, and cognition: The MEMENTO cohort. *Alzheimer’s & Dementia: The Journal of the Alzheimer’s Association*. 2023;19(6):2332–2342.

14. Gottesman RF, Schneider ALC, Albert M, Alonso A, Bandeen-Roche K, Coker L, Coresh J, Knopman D, Power MC, Rawlings A, Sharrett AR, Wruck LM, Mosley TH. Midlife hypertension and 20-year cognitive change: the atherosclerosis risk in communities neurocognitive study. *JAMA neurology*. 2014;71(10):1218–1227.

15. Suvila K, Lima JAC, Yano Y, Tan ZS, Cheng S, Niiranen TJ. Early-but Not Late-Onset Hypertension Is Related to Midlife Cognitive Function. *Hypertension*. 2021;77(3):972–979.

16. Yaffe K, Bahorik AL, Hoang TD, Forrester S, Jacobs DR, Lewis CE, Lloyd-Jones DM, Sidney S, Reis JP. Cardiovascular risk factors and accelerated cognitive decline in midlife. *Neurology*. 2020;95(7):e839–e846.

17. Faraco G, Sugiyama Y, Lane D, Garcia-Bonilla L, Chang H, Santisteban MM, Racchumi G, Murphy M, Rooijen NV, Anrather J, Iadecola C. Perivascular macrophages mediate the neurovascular and cognitive dysfunction associated with hypertension. *The Journal of Clinical Investigation*. 2016;126(12):4674–4689.

18. Pelisch N, Hosomi N, Ueno M, Nakano D, Hitomi H, Mogi M, Shimada K, Kobori H, Horiuchi M, Sakamoto H, Matsumoto M, Kohno M, Nishiyama A. Blockade of AT1 Receptors Protects the Blood–Brain Barrier and Improves Cognition in Dahl Salt-Sensitive Hypertensive Rats. *American Journal of Hypertension*. 2011;24(3):362–368.

19. Walsh KR, Kuwabara JT, Shim JW, Wainford RD. Norepinephrine-evoked salt-sensitive hypertension requires impaired renal sodium chloride cotransporter activity in Sprague-Dawley rats. *American Journal of Physiology. Regulatory, Integrative and Comparative Physiology*. 2016;310(2):R115-124.

20. Gumerlock MK, Neuwelt EA. The effects of anesthesia on osmotic blood-brain barrier disruption. *Neurosurgery*. 1990;26(2):268–277.

21. Antunes M, Biala G. The novel object recognition memory: neurobiology, test procedure, and its modifications. *Cognitive Processing*. 2012;13(2):93–110.

22. Vogel-Ciernia A, Wood MA. Examining Object Location and Object Recognition Memory in Mice. *Current Protocols in Neuroscience*. 2014;69(1):8.31.1-8.31.17.

23. Fuentes-Santamaría V, Alvarado JC, López-Muñoz DF, Melgar-Rojas P, Gabaldón-Ull MC, Juiz JM. Glia-related mechanisms in the anteroventral cochlear nucleus of the adult rat in response to unilateral conductive hearing loss. *Frontiers in Neuroscience*. 2014;8:319.

24. Mowry FE, Peaden SC, Stern JE, Biancardi VC. TLR4 and AT1R mediate blood-brain barrier disruption, neuroinflammation, and autonomic dysfunction in spontaneously hypertensive rats. *Pharmacological Research*. 2021;174:105877.

25. Jiang E, Chapp AD, Fan Y, Larson RA, Hahka T, Huber MJ, Yan J, Chen Q-H, Shan Z. Expression of Proinflammatory Cytokines Is Upregulated in the Hypothalamic Paraventricular Nucleus of Dahl Salt-Sensitive Hypertensive Rats. *Frontiers in Physiology*. 2018;9:104.

26. Ueno M, Sakamoto H, Liao Y-J, Onodera M, Huang C-L, Miyanaka H, Nakagawa T. Blood-brain barrier disruption in the hypothalamus of young adult spontaneously hypertensive rats. *Histochemistry and Cell Biology*. 2004;122(2):131–137.

27. Shi Z, Gan X-B, Fan Z-D, Zhang F, Zhou Y-B, Gao X-Y, De W, Zhu G-Q. Inflammatory cytokines in paraventricular nucleus modulate sympathetic activity and cardiac sympathetic afferent reflex in rats. *Acta Physiologica*. 2011;203(2):289–297.

28. de Kloet AD, Pioquinto DJ, Nguyen D, Wang L, Smith JA, Hiller H, Sumners C. Obesity induces neuroinflammation mediated by altered expression of the renin–angiotensin system in mouse forebrain nuclei. *Physiology & Behavior*. 2014;136:31–38.

29. Stern JE, Son S, Biancardi VC, Zheng H, Sharma N, Patel KP. Astrocytes Contribute to Angiotensin II Stimulation of Hypothalamic Neuronal Activity and Sympathetic Outflow. *Hypertension (Dallas, Tex.: 1979)*. 2016;68(6):1483–1493.

30. Shults CL, Pinceti E, Rao YS, Pak TR. Aging and Loss of Circulating 17β-Estradiol Alters the Alternative Splicing of ERβ in the Female Rat Brain. *Endocrinology*. 2015;156(11):4187–4199.

31. Kim K, Nist KM, Puleo F, McKenna J, Wainford RD. Sex Differences in Dietary Sodium evoked NCC Regulation and Blood Pressure in Male and Female Sprague Dawley, Dahl Salt-Resistant and Dahl Salt-Sensitive Rats. *American Journal of Physiology. Renal Physiology*. 2024;327(2):F277–F289.

32. Olivera S, Graham D. Sex differences in preclinical models of hypertension. *Journal of Human Hypertension*. 2023;37(8):619–625.

33. Sandoval KE, Witt KA. Age and 17β-estradiol effects on blood-brain barrier tight junction and estrogen receptor proteins in ovariectomized rats. *Microvascular Research*. 2011;81(2):198–205.

34. Acosta-Martínez M. Shaping Microglial Phenotypes Through Estrogen Receptors: Relevance to Sex-Specific Neuroinflammatory Responses to Brain Injury and Disease. *Journal of Pharmacology and Experimental Therapeutics*. 2020;375(1):223–236.

35. Loiola RA, Wickstead ES, Solito E, McArthur S. Estrogen Promotes Pro-resolving Microglial Behavior and Phagocytic Cell Clearance Through the Actions of Annexin A1. *Frontiers in Endocrinology*. 2019;10:420.

36. Haring B, Leng X, Robinson J, Johnson KC, Jackson RD, Beyth R, Wactawski-Wende J, von Ballmoos MW, Goveas JS, Kuller LH, Wassertheil-Smoller S. Cardiovascular disease and cognitive decline in postmenopausal women: results from the Women’s Health Initiative Memory Study. *Journal of the American Heart Association*. 2013;2(6):e000369.

37. Drews HJ, Klein R, Lourhmati A, Buadze M, Schaeffeler E, Lang T, Seferyan T, Hanson LR, Frey II WH, de Vries TCGM, Thijssen-van Loosdregt IAEW, Gleiter CH, Schwab M, Danielyan L. Losartan Improves Memory, Neurogenesis and Cell Motility in Transgenic Alzheimer’s Mice. *Pharmaceuticals*. 2021;14(2):166.

38. The SPRINT MIND Investigators for the SPRINT Research Group. Effect of Intensive vs Standard Blood Pressure Control on Probable Dementia: A Randomized Clinical Trial. *JAMA*. 2019;321(6):553–561.

39. Peters R, Xu Y, Fitzgerald O, Aung HL, Beckett N, Bulpitt C, Chalmers J, Forette F, Gong J, Harris K, Humburg P, Matthews FE, Staessen JA, Thijs L, Tzourio C, et al. Blood pressure lowering and prevention of dementia: an individual patient data meta-analysis. *European Heart Journal*. 2022;43(48):4980–4990.

40. Ding J, Davis-Plourde KL, Sedaghat S, Tully PJ, Wang W, Phillips C, Pase MP, Himali JJ, Windham BG, Griswold M, Gottesman R, Mosley TH, White L, Guðnason V, Debette S, et al. Antihypertensive medications and risk for incident dementia and Alzheimer’s disease: a meta-analysis of individual participant data from prospective cohort studies. *The Lancet Neurology*. 2020;19(1):61–70.

41. Tedesco MA, Ratti G, Mennella S, Manzo G, Grieco M, Rainone AC, Iarussi D, Iacono A. Comparison of losartan and hydrochlorothiazide on cognitive function and quality of life in hypertensive patients. *American Journal of Hypertension*. 1999;12(11 Pt 1):1130–1134.

42. Hajjar I, Okafor MO, McDaniel D, Obideen M, Dee E, Shokouhi M, Quyyumi A, Levey AI, Goldstein FC. Effects of candesartan vs. lisinopril on neurocognitive functioning in executive mild cognitive impairment: The CALIBREX trial. *Alzheimer’s & Dementia*. 2020;16(S9):e043313.

43. Santisteban MM, Ahn SJ, Lane D, Faraco G, Garcia-Bonilla L, Racchumi G, Poon C, Schaeffer S, Segarra SG, Körbelin J, Anrather J, Iadecola C. Endothelium-Macrophage Crosstalk Mediates Blood-Brain Barrier Dysfunction in Hypertension. *Hypertension (Dallas, Tex.: 1979)*. 2020;76(3):795–807.

44. Villapol S, Balarezo MG, Affram K, Saavedra JM, Symes AJ. Neurorestoration after traumatic brain injury through angiotensin II receptor blockage. *Brain*. 2015;138(11):3299–3315.

45. Villapol S, Saavedra JM. Neuroprotective Effects of Angiotensin Receptor Blockers. *American Journal of Hypertension*. 2015;28(3):289–299.

**Figure Legends**

**Figure 1: Male, but not female, Sprague-Dawley rats develop age-dependent hypertension and sympathoexcitation. (A)** Mean arterial pressure (MAP; mmHg) measured via acute instrumentation in conscious 3-, 8-, and 16-month-old (MO) male rats (n=5-6/group). **(B)** MAP measured via acute instrumentation in conscious 3-, 8-, 16-month-old female rats (n=6/group). **(C)** Peak change in MAP to ganglionic blockade with hexamethonium (30 mg/kg I.V.) in male rats (n=6/group). **(D)** Peak change in MAP to ganglionic blockade with hexamethonium in female rats (n=6/group). **(E)** Plasma norepinephrine (NE) content (nmol/L) in male rats (n=6/group). **(F)** Plasma NE content (nmol/L) in female rats (n=6/group). Statistical significance was determined using one-way ANOVA with a post-hoc Tukey test, **p<0.01, ***p<0.005, ****p<0.0001.

**Figure 2: Male, but not female, Sprague-Dawley rats develop BBB disruption and neuroinflammation in the PVN with age. (A)** Representative photomicrographs (captured at 10X magnification) from level 2 of the PVN (Bregma -1.72 mm to -2.04 mm) of FITC extravasation as a measure of BBB disruption in male rats at 3-, 8-, and 16-months-old; Scale bar = 200 μm. **(B)** FITC extravasation in level 2 of the PVN in aging male rats (n=6/group). **(C)** Representative photomicrographs (captured at 10X magnification) from level 2 of the PVN (Bregma -1.72 to -2.04) of FITC extravasation as a measure of blood brain barrier disruption in female rats at 3-, 8-, and 16-months-old; Scale bar shown in (A). **(D)** FITC extravasation in level 2 of the PVN in aging female rats (n=6/group). **(E)** Representative photomicrographs (captured at 10X magnification with 40X magnification inset) from level 2 of the PVN (Bregma -1.72 mm to -2.04 mm) of microglia (stained using OX-42/CD11b/c) in male rats at 3-, 8-, and 16-months-old; Scale bar (10X) = 200 μm, Scale bar (40X) = 50 μm. **(F)** Number of active microglia per 80,000 μm^2^ in level 2 of the PVN in aging male rats (n=6/group). **(G)** Total number of microglia per 80,000 μm^2^ in level 2 of the PVN in aging male rats (n=6/group). **(H)** Representative photomicrographs (captured at 10X magnification with 40X magnification inset) from level 2 of the PVN (Bregma -1.72 to -2.04) of microglia (stained using OX-42/CD11b/c) in female rats at 3-, 8-, and 16-months-old; Scale bars shown in (E). **(I)** Number of active microglia per 80,000 μm^2^ in level 2 of the PVN in aging female rats (n=6/group). **(J)** Total number of microglia per 80,000 μm^2^ in level 2 of the PVN in aging female rats (n=6/group). **(K)** Sholl analysis expressed as number of intersections as a function of distance from the soma (μm) in 8 microglia per rat from level 2 of the PVN in 3- vs. 8-month-old male (M) rats (n=6/group). **(L)** Sholl analysis expressed as number of intersections as a function of distance from the soma (μm) in 8 microglia per rat from level 2 of the PVN in 3- vs. 16-month-old male (M) rats (n=6/group). **(M)** Sholl analysis expressed as number of intersections as a function of distance from the soma (μm) in 8 microglia per rat from level 2 of the PVN in 8- vs. 16-month-old male (M) rats (n=6/group). **(N)** Representative photomicrographs (captured at 10X magnification with 20X magnification inset) from level 2 of the PVN (Bregma -1.72 mm to -2.04 mm) of astrocytes (stained using glial fibrillary acidic protein (GFAP)) in male rats at 3-, 8-, and 16-months-old; Scale bar (10X) = 200 μm, Scale bar (20X) = 50 μm. **(O)** GFAP expression presented as percent area of level 2 of the PVN in aging male rats (n=6/group). **(P)** Representative photomicrographs (captured at 10X magnification with 20X magnification inset) from level 2 of the PVN (Bregma -1.72 to -2.04) of astrocytes (stained using GFAP) in female rats at 3-, 8-, and 16-months-old; Scale bars shown in (N). **(Q)** GFAP expression presented as percent area of level 2 of the PVN in aging female rats (n=6/group). Statistical significance was determined using one-way ANOVA with a post-hoc Tukey test, *p<0.05, ***p<0.005, ****p<0.0001. For Sholl analysis (K-M), student’s t-test was used to determine statistical significance, *p<0.05, **p<0.01, ***p<0.005.

**Figure 3: Male, but not female, Sprague-Dawley rats exhibit increased proinflammatory cytokine expression in the PVN with age.** **(A)** Representative photomicrographs of interleukin-6 (IL-6) expression in level 2 of the PVN (Bregma -1.72mm to -2.04mm) of male rats at 3-, 8-, and 16-months-old. **(B)** IL-6 expression as percent area of the PVN in male Sprague Dawley rats (N=5-6/group). **(C)** Representative photomicrographs of IL-6 expression in level 2 of the PVN (Bregma -1.72mm to -2.04mm) of female rats at 3-, 8-, and 16-months-old. **(D)** IL-6 expression as percent area of the PVN in female Sprague Dawley rats (N=5-6/group). **(E)** Representative photomicrographs of tumor necrosis factor-⍺ (TNF-⍺) expression in level 2 of the PVN (Bregma -1.72mm to -2.04mm) of male rats at 3-, 8-, and 16-months-old. **(F)** TNF-⍺ expression as percent area of the PVN in male Sprague Dawley rats (N=5-6/group). **(G)** Representative photomicrographs of TNF-⍺ expression in level 2 of the PVN (Bregma -1.72mm to -2.04mm) of female rats at 3-, 8-, and 16-months-old. **(H)** TNF-⍺ expression as percent area of the PVN in female Sprague Dawley rats (N=5-6/group). Scale bar = 200μm. Statistical significance was determined using one-way ANOVA with a post-hoc Tukey test of significance, *p<0.05, ***p<0.005, ****p<0.0001.

**Figure 4: Male, but not female, Sprague-Dawley rats develop cognitive impairment. (A)** Representative schematic of the novel object recognition task- a measure of recognition memory. **(B)** Representative schematic of the object location task- a measure of spatial memory. **(C)** Discrimination index of novel object in 3-, 8-, and 16-month-old male rats (N=5-6/group). **(D)** Discrimination index of novel object in 3-, 8-, and 16-month-old female rats (N=5-6/group). **(E)** Discrimination index of novel location in 3-, 8-, and 16-month-old male rats (N=5-6/group). **(F)** Discrimination index of novel location in 3-, 8-, and 16-month-old female rats (N=5-6/group). **(G)** Time spent at novel object (expressed as percent of total time) in 3-, 8-, and 16-month-old male rats (N=5-6/group). **(H)** Time spent at novel object (expressed as percent of total time) in 3-, 8-, and 16-month-old female rats (N=5-6/group). **(I)** Time spent at novel location (expressed as percent of total time) in 3-, 8-, and 16-month-old male rats (N=5-6/group). **(J)** Time spent at novel location (expressed as percent of total time) in 3-, 8-, and 16-month-old female rats(N=5-6/group). Statistical significance was determined using one-way ANOVA with a post-hoc Tukey test of significance, *p<0.05, **p<0.01, ***p<0.005.

**Figure 5: Losartan improves recognition memory in 16-month-old male Sprague-Dawley rats with established hypertension. (A)** Mean arterial pressure (MAP; mmHg) measured via acute instrumentation in male rats at 16-months-old (MO) with no treatment, with losartan (LOS; 3 mg/kg/day s.c., 21-days), or with hydrochlorothiazide (HCTZ; 4 mg/kg/day s.c., 14-days); (n=5-6/group), Data used for 16-month-old male is also used in Figure 1A. **(B)** Discrimination index of novel object recognition task performance in losartan treated rats before (PreTx) and after treatment (n=6/group). **(C)** Percent of time spent at novel object in losartan treated rats before (PreTx) and after treatment (n=6/group). **(D)** Discrimination index of novel object recognition task performance in hydrochlorothiazide treated rats before (PreTx) and after treatment (n=6/group). **(E)** Percent of time spent at novel object in hydrochlorothiazide treated rats before (PreTx) and after treatment (n=6/group). **(F)** Discrimination index of object location task performance in losartan treated rats before (PreTx) and after treatment (n=6/group). **(G)** Percent of time spent at novel location in losartan treated rats before (PreTx) and after treatment (n=6/group). **(H)** Discrimination index of object location task performance in hydrochlorothiazide treated rats before (PreTx) and after treatment (n=6/group). **(I)** Percent of time spent at novel location in hydrochlorothiazide treated rats before (PreTx) and after treatment (n=6/group). Statistical significance for (A) was determined using one-way ANOVA with a post-hoc Tukey test, ****p<0.0001; Statistical significance for (B-I) was determined using paired samples t-test, *p<0.05.

**Figure 6: Losartan improves BBB disruption and neuroinflammation in the PVN of 16-month-old male Sprague-Dawley rats with established hypertension. (A)** Representative photomicrographs (captured at 10X magnification) from level 2 of the PVN (Bregma -1.72 mm to -2.04 mm) of FITC extravasation as a measure of BBB disruption in male rats at 16-months-old (MO) with no treatment, with losartan (LOS; 3 mg/kg/day s.c., 21-days), or with hydrochlorothiazide (HCTZ; 4 mg/kg/day s.c., 14-days); Scale bar = 200 μm. Representative image for 16-month-old male is also used in Figure 2A. **(B)** FITC extravasation in level 2 of the PVN in male rats at 16-months-old with no treatment, with losartan, or with hydrochlorothiazide; (n=5-6/group), Data used for 16-month-old male is also used in Figure 2B. **(C)** Representative photomicrographs (captured at 10X magnification with 40X magnification inset) from level 2 of the PVN (Bregma -1.72 mm to -2.04 mm) of microglia (stained using OX-42/CD11b/c) in male rats at 16-months-old with no treatment, with losartan, or with hydrochlorothiazide; Scale bar (10X) = 200 μm, Scale bar (40X) = 50 μm. Representative image for 16-month-old male is also used in Figure 2E. **(D)** Number of active microglia per 80,000 μm^2^ in level 2 of the PVN in male rats at 16-months-old with no treatment, with losartan, or with hydrochlorothiazide; (n=6/group). Data used for 16-month-old male is also used in Figure 2F. **(E)** Total number of microglia per 80,000 μm^2^ in level 2 of the PVN in male rats at 16-months-old with no treatment, with losartan, or with hydrochlorothiazide; (n=6/group); Data used for 16-month-old male is also used in Figure 2G. **(F)** Sholl analysis expressed as number of intersections as a function of distance from the soma (μm) in 8 microglia per rat from level 2 of the PVN in 16- vs. 16-month-old + losartan male (M) rats (n=6/group); Data used for 16-month-old male is also used in Figure 2L-M. **(G)** Sholl analysis expressed as number of intersections as a function of distance from the soma (μm) in 8 microglia per rat from level 2 of the PVN in 16- vs. 16-month-old + hydrochlorothiazide male (M) rats (n=6/group); Data used for 16-month-old male is also used in Figure 2L-M. **(H)** Representative photomicrographs (captured at 10X magnification with 20X magnification inset) from level 2 of the PVN (Bregma -1.72 mm to -2.04 mm) of astrocytes (stained using glial fibrillary acidic protein (GFAP)) in male rats at 16-months-old with no treatment, with losartan, or with hydrochlorothiazide; Scale bar (10X) = 200 μm, Scale bar (20X) = 50 μm. Representative image for 16-month-old male is also used in Figure 2N. **(I)** GFAP expression presented as percent area of level 2 of the PVN in male rats at 16-months-old with no treatment, with losartan, or with hydrochlorothiazide; (n=6/group); Data used for 16-month-old male is also used in Figure 2O. **(J)** Representative photomicrographs of interleukin-6 (IL-6) expression in level 2 of the PVN (Bregma -1.72mm to -2.04mm) of male rats at 16-months-old with no treatment or with losartan; (N=5-6/group). Scale bar (10X) = 200 μm; Representative image for 16-month-old male is also used in Figure 3A. **(K)** IL-6 expression as percent area of the PVN in male Sprague Dawley rats at 16-months-old with no treatment or with losartan (N=5-6/group), Data used for 16-month-old male is also used in Figure 3B. **(L)** Representative photomicrographs of tumor necrosis factor-⍺ (TNF-⍺) expression in level 2 of the PVN (Bregma -1.72mm to -2.04mm) of male rats at 16-months-old with no treatment or with losartan (N=5-6/group). Scale bar (10X) = 200 μm; Representative image for 16-month-old male is also used in Figure 3E. **(M)** TNF-⍺ expression as percent area of the PVN in male Sprague Dawley rats at 16-months-old with no treatment or with losartan (N=5-6/group); Data used for 16-month-old male is also used in Figure 3F. Statistical significance was determined using one-way ANOVA with a post-hoc Tukey test (B,D-E,I), *p<0.05, **p<0.01, ***p<0.005, ****p<0.0001. For Sholl analysis and cytokine expression (F-G,K,M), student’s t-test was used to determine statistical significance, ***p<0.005, ****p<0.0001.

**Figure 7: Schematic of proposed hypothesis.** BBB disruption and neuroinflammation in the paraventricular nucleus (PVN) contributes to increased sympathetic outflow and hypertension in aged male Sprague-Dawley rats. Age-dependent hypertension is associated with increased cognitive impairment. While hydrochlorothiazide, an NCC antagonist, only lowers blood pressure, losartan, an AT1R lowers blood pressure, attenuates PVN neuroinflammation and improves cognitive function in aged male rats.

**Table 1: Familiarization from novel object recognition task and object location tasks in 3-, 8-, and 16-month-old male and female Sprague-Dawley rats.**

| Age and Sex | Novel Object Recognition Task | Object Location Task |
| --- | --- | --- |
|  | Familiarization Index | Familiarization Index |
| 3 MO Male | 0.038 ± 0.146 | 0.008 ± 0.085 |
| 8 MO Male | 0.092 ± 0.227 | 0.072 ± 0.329 |
| 16 MO Male | 0.033 ± 0.046 | -0.006 ± 0.059 |
| 3 MO Female | 0.013 ± 0.107 | -0.003 ± 0.127 |
| 8 MO Female | 0.300 ± 0.341 | 0.007 ± 0.175 |
| 16 MO Female | 0.035 ± 0.154 | 0.109 ± 0.226 |

Familiarization index, defined as time spent at left object minus time spent at right object divided by time spent at both objects, calculated for familiarization of the novel object recognition and object location tasks in 3-, 8-, and 16-month-old (MO) male and female Sprague-Dawley rats. Data is presented as group mean ± standard deviation, N=5-6/group.

**Table 2: Familiarization from novel object recognition task and object location tasks in 16-month-old male Sprague-Dawley rats pre- and post-treatment with losartan or hydrochlorothiazide.**

| Treatment | | Novel Object Recognition Task | Object Location Task |
| --- | --- | --- | --- |
|  |  | Familiarization Index | Familiarization Index |
| Losartan | PreTx | 0.181 ± 0.441 | -0.073 ± 0.054 |
|  | PostTx | 0.207 ± 0.388 | 0.145 ± 0.176 |
| Hydrochlorothiazide | PreTx | 0.021 ± 0.119 | 0.064 ± 0.251 |
|  | PostTx | -0.029 ± 0.220 | -0.061 ± 0.137 |

Familiarization index, defined as time spent at left object minus time spent at right object divided by time spent at both objects, calculated for familiarization of the novel object recognition and object location tasks in 16-month-old (MO) male Sprague-Dawley rats before treatment (Tx) and after Tx with either losartan (s.c. 3 mg/kg/day, 21 days) or hydrochlorothiazide (s.c. 4 mg/kg/day, 14 days). Data is presented as group mean ± standard deviation, N=5-6/group.
